# An Implantable Phototriggered Prodrug Depot Patch Enables Actively Programmable Drug Release for Post‐Myocardial Infarction Therapy

**DOI:** 10.1002/advs.76161

**Published:** 2026-06-26

**Authors:** Haipeng Lu, Kaicheng Deng, Zhang Zhang, Qirui Wang, Weijing Gao, Liyin Shen, Lei Zhang, Wenting Hu, Yang Zhu, Zhengwei Mao, Tanchen Ren

**Affiliations:** ^1^ Department of Cardiology State Key Laboratory of Transvascular Implantation Devices Heart Regeneration and Repair Key Laboratory of Zhejiang Province The Second Affiliated Hospital School of Medicine Zhejiang University Hangzhou China; ^2^ MOE Key Laboratory of Macromolecular Synthesis and Functionalization Department of Polymer Science and Engineering Zhejiang University Hangzhou China; ^3^ Stomatology Hospital School of Stomatology School of Medicine Zhejiang University Hangzhou China; ^4^ State Key Laboratory of Modern Optical Instrumentation College of Optical Science and Engineering Zhejiang University Hangzhou China

**Keywords:** actively programmable drug release, integrated implantable devices, myocardial infarction (MI), phototriggered prodrugs

## Abstract

The prolonged healing process after myocardial infarction (MI) necessitates extended pharmacotherapy. While microenvironment‐responsive implantable drug delivery systems can achieve various long‐term release patterns, they offer only passive, non‑programmable profiles. Light‐controlled strategies enable precise external regulation but face two critical barriers: the lack of integrated devices combining stable drug reservoirs with implantable light platforms, and the inherent light–release coupling of conventional phototriggered systems that necessitates continuous illumination for sustained dosing. Here, we develop an implantable phototriggered prodrug depot patch (iPDP) enabling actively programmable drug release after implantation. The iPDP fuses a phototriggered sustained‐release prodrug hydrogel (GTel‑hydrogel) and a percutaneous light‑guiding device. The prodrug GTel features a self‐immolative linker, allowing a brief optical trigger to initiate sustained, light‑independent release. The covalently conjugated prodrug ensures minimal leakage, with dose per release precisely controlled by illumination parameters (intensity/duration) as the programming code. In a rat MI model, the iPDP delivered a triple‑dose, actively programmed regimen. Compared with a single release of an equivalent total dose, this fractionated programmed strategy showed superior efficacy in improving cardiac function and suppressing inflammation. The iPDP enables post‑implantation active control from a single device, offering a promising strategy for long‐term dynamic post‐MI therapy.

## Graphical abstract

An implantable phototriggered prodrug depot patch (iPDP) is presented that enables actively programmable drug release post‐implantation. Covalently conjugated prodrug ensures minimal leakage; illumination parameters serve as a code to precisely control dose per release. In a rat myocardial infarction model, fractionated programmed release outperformed a single equivalent bolus, significantly improving cardiac function and suppressing inflammation.

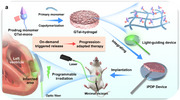

## 1 Introduction

Myocardial infarction (MI) remains one of the leading threats to human health. Post‐MI repair is a chronic pathological process that often requires pharmacological intervention over weeks or even months to suppress excessive inflammatory responses and ventricular remodeling [1, 2]. Microenvironment‐responsive, locally implantable or injectable drug delivery systems can achieve various long‐term release patterns, such as sustained release [3], pulsatile release [4, 5], and sequential release [6, 7], and have made certain progress in the treatment of chronic diseases like post‐infarction repair [8, 9, 10]. These systems are typically based on microenvironment‐responsive materials that, after implantation, release drugs in a predetermined manner in response to endogenous stimuli such as specific enzymes [11], reactive oxygen species [12, 13, 14], or pH [15, 16]. However, their release kinetics are fundamentally passive and non‑programmable—the release profiles are either materially preset or dictated by the volatile internal environment [17, 18]. This makes it difficult for them to meet the demand for dynamic, active control over the timing and dosage of drug release required during the complex healing process of chronic diseases such as MI.

To achieve “active programming” of local drug delivery systems, external stimuli such as magnetic fields [19], ultrasound [20], and light [21, 22] have been explored. Among these, optical control is particularly promising due to its exceptional spatiotemporal precision and the tunability of its parameters (e.g., intensity, duration), which can serve as direct programming inputs [22]. To overcome the limited tissue penetration depth of light, implantable light‑emitting devices have been developed. These include wirelessly powered micro‑LEDs as well as waveguide‑based patches or fibers that can be percutaneously connected, enabling light delivery to deep tissues like the heart [23, 24, 25, 26, 27, 28]. However, two distinct limitations persist in current research paradigms. First, many studies employ implantable optical devices paired with separately injected photoactivatable agents, which fundamentally rely on non‑programmable intravenous administration and lack a long‑term, localized drug reservoir [25, 26, 27]. Second, even in systems where drug carriers (e.g., photothermal release coatings) are physically integrated with light guides, the drug is typically physically encapsulated [24, 28]. This format often results in premature leakage and imprecise control over the release profile, undermining the stability and programmability required for long‑term therapy. Thus, a critical gap remains in developing a deeply integrated implantable device that combines a stable and precisely programmable drug reservoir with an implantable light‑emitting platform.

Phototriggered prodrug chemistry, in which drugs are covalently conjugated via photocleavable linkers, provides an ideal materials solution by design [29, 30, 31, 32, 33]. It ensures minimal baseline leakage and establishes a quantitative relationship between light dose and drug release [33]. In most designs, however, drug release is strictly coupled to illumination: photolysis immediately liberates the active agent in a burst release fashion, and release ceases when light is removed [34, 35, 36]. For systems intended to provide sustained release, each dosing event therefore requires continuous light exposure throughout the entire release period—a substantial practical burden for multi‑cycle therapy in chronic diseases. By contrast, incorporating a self‐immolative spacer between the photocleavable linker and the drug can introduce a rate‑limiting elimination step [37, 38]. Because photolysis is typically fast, this elimination step often becomes slower than the initial cleavage, thereby creating a temporal delay between light absorption and drug liberation. This “trigger‑decoupled” release profile has been noted in the literature, but primarily as an undesirable background leakage to be minimized [39]. For actively programmable, multi‑cycle drug delivery, however, such decoupling enables a brief optical trigger to initiate sustained therapeutic coverage while dramatically reducing light source connection times—without sacrificing the “active” nature of programming, since each release event remains unequivocally commanded by a deliberate light input. This decoupling mechanism thus represents a valuable yet overlooked design opportunity, particularly when engineered into a deeply integrated, implantable device. Consequently, creating a stable, programmable prodrug depot with tailored self‐immolative kinetics, and integrating this depot with an implantable light‑emitting platform into a single device, presents a compelling yet under‑addressed strategy for achieving truly programmable, long‑term therapy in deep tissues.

**SCHEME 1:**
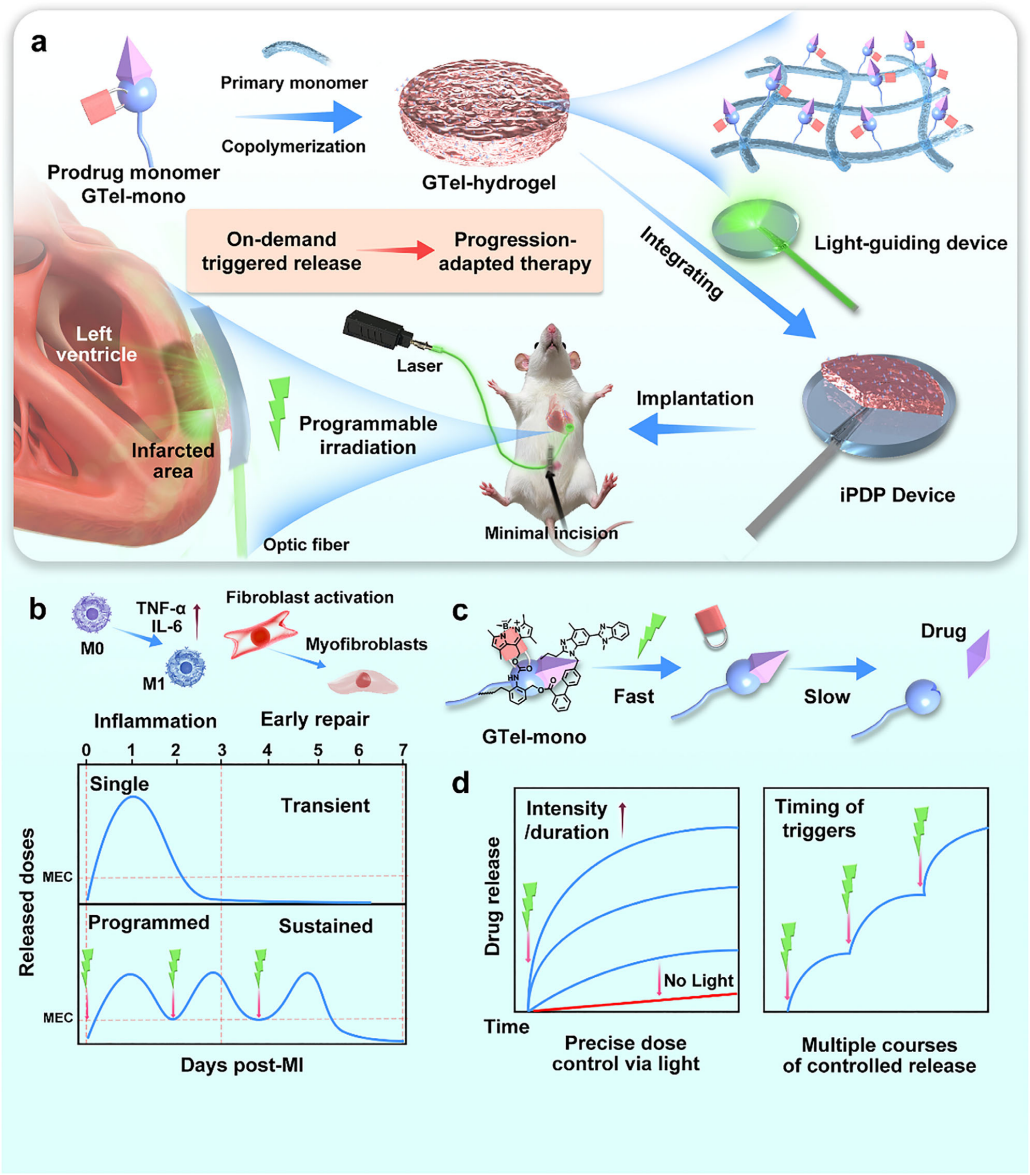
Schematic of the integrated implantable phototriggered prodrug depot patch (iPDP) for active, programmable therapy after myocardial infarction. (a) Workflow of the iPDP platform from fabrication to therapeutic outcome. The phototriggered prodrug monomer (GTel‑mono) is copolymerized to form a prodrug‑loaded hydrogel (GTel‑hydrogel), which is then shaped and integrated with a customized light‑guiding device. The assembled iPDP is implanted onto the infarcted heart, with its optical fiber tunneled subcutaneously. Therapy is delivered via percutaneous connection to an external laser (520 nm), whose illumination parameters (intensity, duration) program the drug dose per trigger. (b) Rationale for active programming and strategy comparison: a single bolus release (Transient intervention) provides only brief coverage above the minimum effective concentration (MEC, dashed line), whereas the actively programmed, fractionated release (Sustained intervention) via iPDP maintains therapeutic levels over an extended period, thereby better matching the dynamic pathological continuum. (c) Molecular activation mechanism. The prodrug is activated via rapid photolysis (in minutes) followed by sustained hydrolysis (in days) to release the active drug, telmisartan. (d) Multidimensional programmability of the iPDP. (Left) Precise release profile control by light: The drug release per trigger is directly governed by illumination parameters (intensity/duration), with minimal baseline leakage without light (red curve). (Right) Temporal programming of release courses: Segmented illumination (e.g., at days 0, 2, 4) produces a corresponding segmented release profile, enabling a temporally programmed regimen from a single implant.

In this study, we developed an integrated implantable phototriggered prodrug depot patch (iPDP) for post‐MI therapy, which was constructed by deeply integrating a unique phototriggered sustained‐release prodrug hydrogel (GTel‐hydrogel) with a customized light‐guiding device to form a percutaneous platform, thereby enabling truly active and programmable drug release in deep tissues (Scheme 1). Within this hydrogel, the prodrug GTel features a self‐immolative 2,6‐bis(hydroxymethyl)aniline linker, allowing the covalently conjugated telmisartan to undergo rapid photoactivation upon triggering, followed by sustained light‑independent hydrolysis and release of the active drug, with negligible baseline leakage. This two‑stage chemical cascade decouples the brief optical “command” (minutes) from the prolonged therapeutic “execution” (days)—each brief illumination triggers a controlled release program that sustains therapeutic levels over days, without requiring frequent light exposure. The integration of the prodrug into the hydrogel network translated its molecular photo‐response into a pulsatile release profile with tunable kinetics. This design enables precise dose control per release event through programmable illumination parameters (e.g., duration and power), and supports multiple on‑demand release cycles from a single implant. The customized light‐guiding component functioned as a percutaneous interface, allowing clinicians to execute multiple on‐demand commands post‐implantation, including precise control over administration timing and dosage, without the need for repeated surgical interventions. Telmisartan exerts its therapeutic effects by targeting both inflammatory and fibrotic processes that evolve dynamically after MI [40, 41, 42], suggesting that a fractionated, actively programmed regimen could provide more sustained coverage across these distinct phases than a single release. In a rat MI model, we demonstrated that this iPDP‐based active fractionated dosing strategy significantly outperformed a single administration of an equivalent total dose in improving cardiac function and suppressing inflammation. By transforming each brief optical input into sustained therapeutic output, the iPDP establishes a new paradigm for post‐implantation active programming—offering a highly promising strategy for the long‐term dynamic post‐MI therapy.

## 2 Results and Discussion

### 2.1 Synthesis and Characterization of the Phototriggered Prodrug (GTel)

The phototriggered prodrug GTel was synthesized via a multistep synthetic route with high yield and purity. The synthesis of the designed GTel started with coupling a triphosgene‐activated, *tert*‐butyldimethylsilyl‐protected 2,6‐bis(hydroxymethyl)aniline linker to DM‐BODIPY‐OH, followed by hydroxyl deprotection and conjugation with telmisartan (Scheme S1). The products were characterized by ^1^H nuclear magnetic resonance spectrometry (^1^H NMR), ^13^C NMR, and high‐resolution mass spectrometry (HRMS) (Figures S1,22). Telmisartan was selected for its established therapeutic benefits in post‑MI repair and for its structural compatibility with our prodrug design. The carboxyl group of telmisartan enables direct ester conjugation to the self‑immolative spacer, a strategy that preserves drug activity and has been successfully employed in other macromolecular telmisartan prodrugs [43].

**FIGURE 1:**
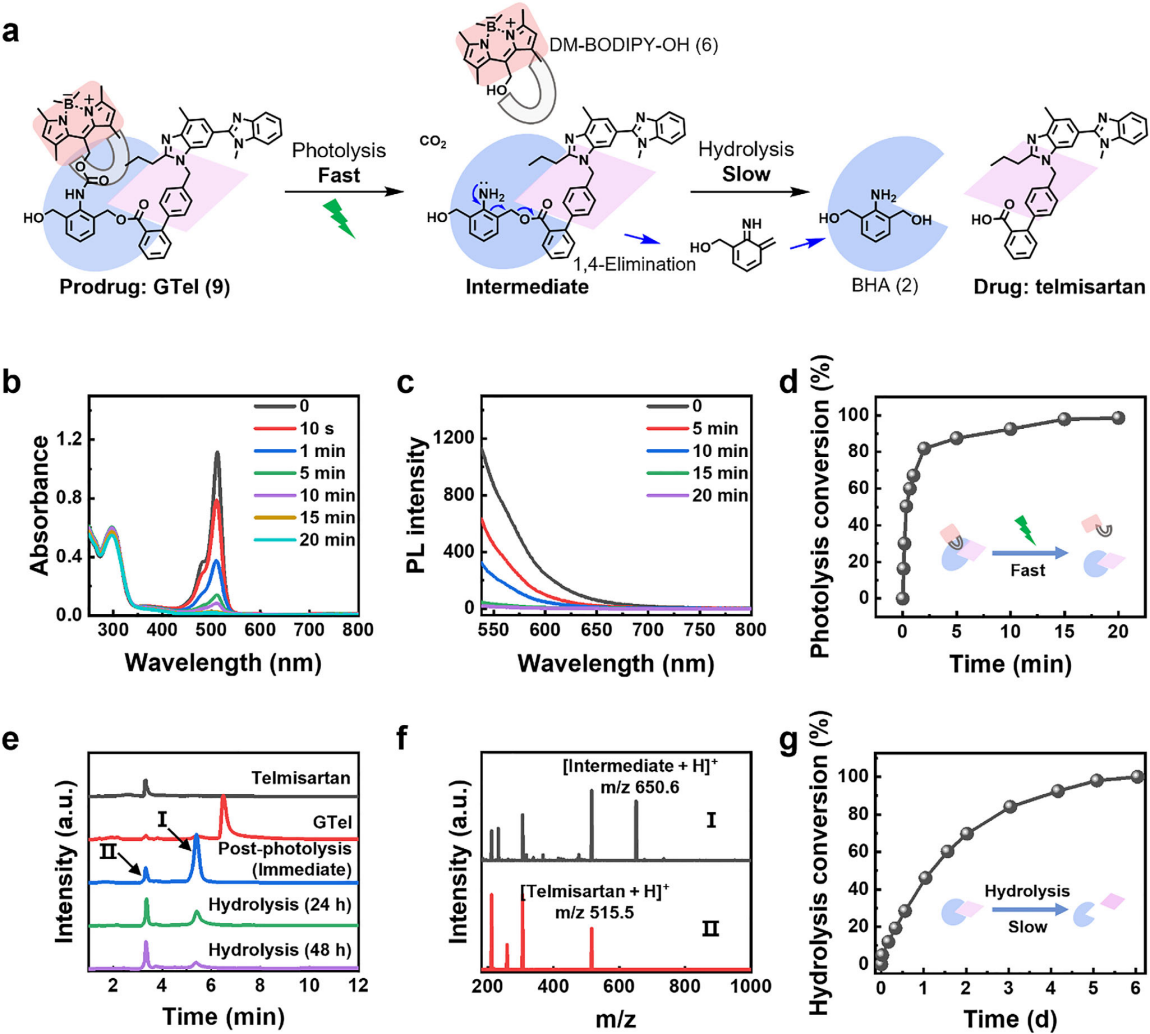
Phototriggered cascade activation kinetics of the GTel prodrug. (a) Schematic illustration of the transient phototriggered cascade activation mechanism. Rapid photolysis of the BODIPY‑carbamate bond initiates a slower conversion via sequential 1,4‑elimination and hydrolysis, ultimately producing telmisartan. (b) UV–vis absorption spectra of GTel under continuous light irradiation. The sample (30 µg mL^−1^ in 60% acetonitrile aqueous solution) was irradiated with a 520 nm LED light source at an intensity of 50 mW cm^−2^. (c) Time‑dependent photoluminescence (PL) emission spectra (*λ*_ex_ = 520 nm) of GTel under the same irradiation conditions. (d) Photolysis conversion profile of GTel calculated from the decay of the characteristic absorbance at 512 nm (*λ*_max_). (e) Liquid chromatography analysis of telmisartan, intact GTel, and GTel following 520 nm LED irradiation (20 min, 50 mW cm^−2^), with detection immediately post‑photolysis and after 24 and 48 h of hydrolysis. (f) Mass spectra of the two major chromatographic peaks (from panel e) at retention times 5.3 min (I) and 3.3 min (II) from GTel analyzed immediately after 520 nm LED irradiation. (g) Time‐dependent conversion profile of GTel to telmisartan in solution at a concentration of 30 µg mL^−1^ following the initial photoirradiation as determined by HPLC.

The GTel was designed to operate via a phototriggered cascade activation mechanism (Figure 1a), wherein illumination at 520 nm first cleaves the DM‐BODIPY‐carbamate bond [44], generating an amine intermediate that spontaneously undergoes 1,4‐elimination via a self‐immolative linker [37, 38], ultimately liberating the active drug telmisartan via ester hydrolysis. The 2,6‐bis(hydroxymethyl)aniline spacer was chosen for its three orthogonal functional groups, enabling modular conjugation to the DM‐BODIPY photocage, to telmisartan, and to the hydrogel network. The DM‐BODIPY photocage was selected for its efficient photolysis at 520 nm (visible light), high conversion yield (>95%), and rapid uncaging kinetics, which together enable precise light‐triggered activation [44, 45, 46]. Importantly, the photocage and spacer are kept as separate modules to simplify synthesis, allow independent optimization of each component, and facilitate covalent integration of the entire construct into the hydrogel via one of the spacer's hydroxyl groups. The activation kinetics of GTel were monitored by ultraviolet–visible (UV–vis) and photoluminescence (PL) spectroscopy (Figure 1b,c). The spectral intensity progressively decreased and stabilized within 20 min under continuous irradiation. The corresponding photolysis conversion increased rapidly and reached a plateau of over 95% within the same period (Figure 1d), confirming a rapid and nearly complete photolytic degradation of GTel. Liquid chromatography/mass spectrometry analysis was conducted to identify the photolysis products (Figure 1e,f). After irradiation, the chromatogram showed complete consumption of GTel and the appearance of two new peaks (I and II). Mass spectrometry identified peak I (m/z 650.6 [M+H]^+^) as the 2,6‐bis(hydroxymethyl)aniline‐telmisartan conjugate, confirming the key intermediate, while peak II (m/z 515.5 [M+H]^+^) matched telmisartan. Over the next 48 h, peak I gradually diminished as peak II increased (Figure 1e), indicating slow hydrolysis of the intermediate to the final drug. The conversion from prodrug to telmisartan followed a sustained time course, with only 46.1% yield achieved within 1 day and 69.7% after 2 days (Figure 1g). This two‐stage cascade release, marked by rapid photolysis followed by rate‐limiting hydrolysis, distinguishes GTel from conventional phototriggered prodrugs that yield the active drug immediately upon irradiation. Such controlled kinetics provide a strong rationale for incorporating GTel into a hydrogel network to construct a phototriggered drug depot.

The distinct hydrolysis kinetics of GTel before and after photoactivation are attributed to the self‑immolative spacer design. Under dark conditions, the ester bond linking telmisartan to the 2,6‑bis(hydroxymethyl)aniline spacer lacks activation from neighboring electron‑donating groups, resulting in slow intrinsic hydrolysis and minimal baseline leakage. Upon 520 nm irradiation, photolysis of the DM‑BODIPY‑carbamate bond releases a free amine on the spacer, triggering a 1,4‑elimination that generates a para‑hydroxybenzyl alcohol intermediate. The para‑hydroxyl group acts as an electron‑donating substituent that stabilizes the tetrahedral intermediate during ester hydrolysis, thereby accelerating the release of telmisartan. This “trigger‑decoupled” mechanism, enabled by the self‑immolative spacer [38, 47], provides the chemical basis for the sustained, light‑independent release observed upon photoactivation.

### 2.2 Synthesis and Characterization of the GTel‐Hydrogel

**FIGURE 2:**
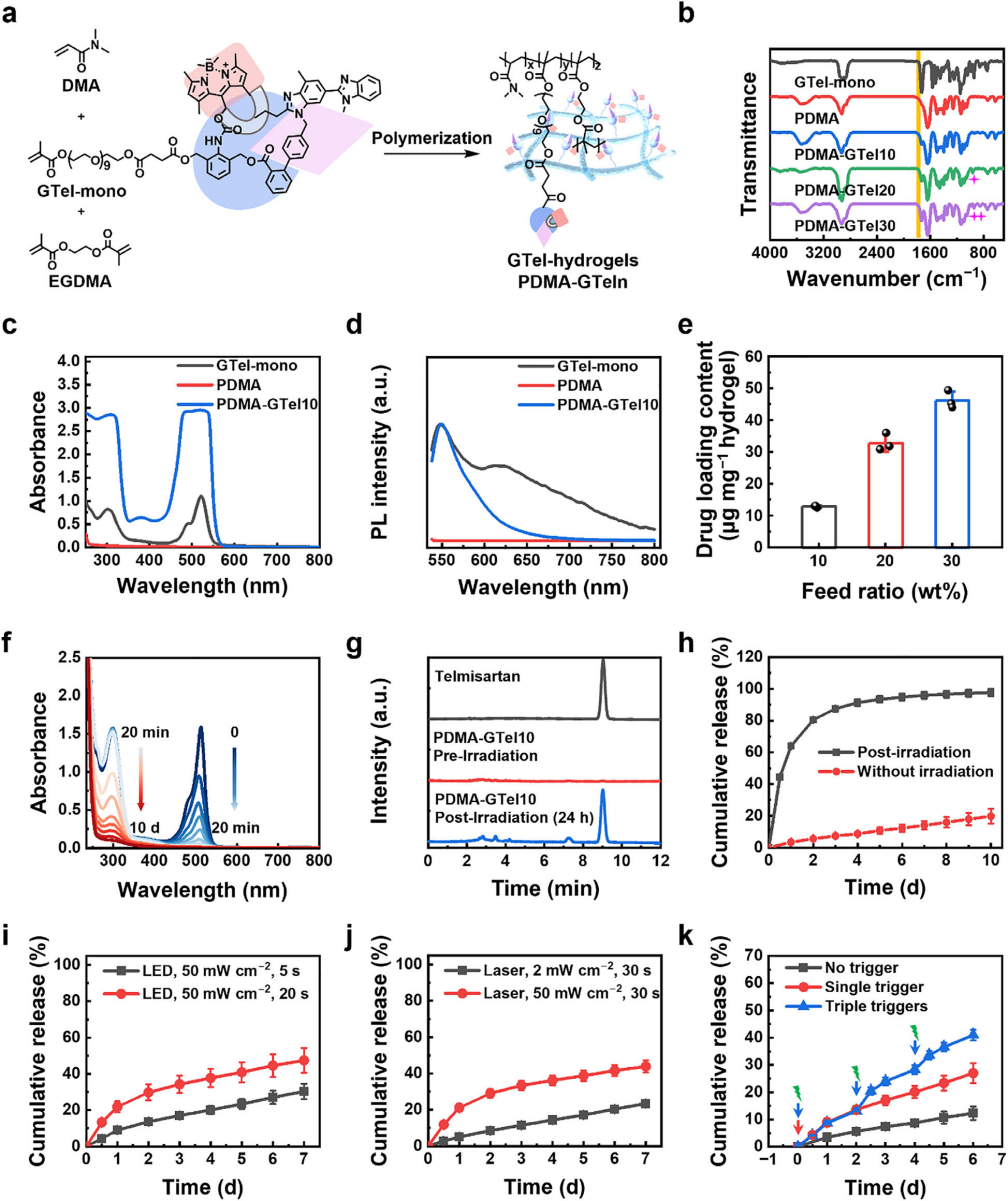
Synthesis, characterization, and programmable phototriggered drug release of GTel‐hydrogels. (a) Schematic illustration of the formation process for prodrug‐containing hydrogels. (b) FT‐IR spectra of the GTel‐mono, the blank hydrogel PDMA, and the PDMA‐GTeln series. The yellow line marks a new peak at 1730 cm^−1^, corresponding to the stretching vibration of the ester carbonyl (C═O) group, and its intensity increases with the GTel‑mono feed ratio. The asterisk indicates an increase in intensity of characteristic peaks at 945 and 856 cm^−1^ with the GTel‑mono feed ratio. (c) UV–vis absorption and (d) PL emission (*λ*_ex_ = 520 nm) spectra of the GTel‐mono, blank PDMA hydrogel, and PDMA‐GTel10 hydrogel. (e) Drug loading capacity of hydrogels with varying GTel monomer feed ratios. (f) Validation of transient phototriggering and sustained hydrolysis: UV–vis absorption spectra of PDMA‐GTel3 hydrogel monitored during and after LED illumination (50 mW cm^−2^). Spectra were tracked during the 20‐min illumination phase (gradient from dark blue at 0 min to white at 20 min) and during the subsequent 10‐day hydrolysis phase in the dark (gradient from white at day 1 to dark red at day 10). Arrows indicate the direction of spectral change over time. (g) HPLC chromatograms of telmisartan standard (retention time = 9 min) and incubation medium from PDMA‐GTel10 hydrogel collected before (Pre‐Irradiation) and 24 h after (Post‐Irradiation) a light trigger (LED, 50 mW cm^−2^, 20 min). (h) Release and leakage profiles of PDMA‐GTel10 hydrogel. The release profile was obtained after a single illumination (LED, 50 mW cm^−2^, 20 min), while the leakage profile was monitored in the dark without any illumination. (i) Release profiles of PDMA‐GTel10 after varying durations of LED illumination (50 mW cm^−2^). (j) Release profiles of PDMA‐GTel10 after varying power densities of laser illumination (30 s duration). (k) Release profiles of PDMA‐GTel10 under different phototriggering programs. Release from the iPDP device under three conditions: no trigger (baseline), a single trigger at day 0, and triple triggers at days 0, 2, and 4. All triggers consisted of brief irradiation (LED, 50 mW cm^−2^, 5 s). Arrows with lightning symbols indicate the timing of illumination (red: single; blue: triple). Data are presented as mean ± SD (*n* = 3).

To enable hydrogel incorporation, GTel was first functionalized by esterifying its remaining hydroxyl group with a carboxylic acid‐terminated oligo(ethylene glycol) monomethacrylate (OEGMA‐COOH). This yielded a polymerizable, phototriggered prodrug‐containing monomer GTel‐mono (Scheme S1). Its structure was confirmed by ^1^H NMR, ^13^C NMR, and HRMS (Figures S23–S25). GTel‐mono was then copolymerized via radical polymerization with *N*, *N*‐dimethylacrylamide (DMA) as the primary monomer and ethylene glycol dimethacrylate (EGDMA) as the crosslinker. The feed ratios are detailed in Table S1. The resulting hydrogels were designated as PDMA‐GTeln, where n is the mass percentage of GTel‐mono in the total monomer feed (Figure 2a). For subsequent implantation, the hydrogels were fabricated with a controlled thickness of 165 ± 5 µm using a glass slide mold (Scheme S2). This thickness was chosen to minimize physical interference with cardiac motion while maintaining high optical transparency.

The feed‐ratio‐dependent incorporation of GTel‐mono into the copolymerized hydrogel was confirmed by Fourier transform infrared spectroscopy (FT‐IR) (Figure 2b). In PDMA‐GTeln, the intensity of the ester carbonyl (C═O) stretching band at 1730 cm^−1^ correlated positively with the GTel‐mono feed ratio. Furthermore, the characteristic peaks of GTel‐mono at 1568, 1307, 990, 945, and 856 cm^−1^ also intensified at higher feed ratios.

The PDMA‐GTel hydrogel was further characterized by UV–vis absorption spectroscopy, as both the DM‐BODIPY and telmisartan motifs in GTel‐mono are intrinsic chromophores (Figure 2c). PDMA‐GTel10 exhibited the characteristic absorption profile of GTel‐mono, with distinct peaks at 299, 490, and 521 nm. In contrast, the blank PDMA hydrogel showed negligible absorption, confirming the successful incorporation of the prodrug GTel. Similar UV–vis absorption spectra were obtained for the PDMA‑GTel20 formulation (Figure S26a). To avoid signal saturation at the high GTel‐mono loading (10% feed ratio), a complementary measurement was performed on a hydrogel with a lower GTel‐mono content (3% feed ratio). Notably, the spectrum of PDMA‐GTel3 (dark blue curve at 0 min in Figure 2f) closely matched that of the free GTel‐mono solution, verifying that the chemical structure of the prodrug remained within the hydrogel.

The PDMA‐GTel hydrogel was also analyzed by PL emission spectroscopy, owing to the characteristic PL emission of the DM‐BODIPY moiety in GTel‐mono under 520 nm excitation (Figure 2d), and comparable emission was observed for PDMA‑GTel20 (Figure S26b). For PDMA‐GTel10, a broad emission band centered at 550 nm was observed—in contrast to free GTel‐mono in solution, which displayed two distinct peaks at 547 and 614 nm corresponding to DM‐BODIPY monomer and excimer emissions. This difference arises because the crosslinked network restricts molecular motion, thereby suppressing excimer formation.

The drug loading content of PDMA‐GTeln hydrogels increased steadily with the GTel‑mono feed ratio (10%, 20%, and 30%) (Figure 2e). This direct correlation shows that the drug payload can be precisely tuned through formulation, offering a straightforward way to adjust the dose as needed.

### 2.3 Phototriggered, Sustained‐Release Behavior and Stability of the GTel‐Hydrogel

Based on the established two‐stage cascade mechanism of the prodrug GTel, the corresponding GTel‐hydrogels were designed to function as a phototriggered, sustained‐release depot. This design function was verified by tracking the temporal evolution of the UV–vis absorption spectra of the GTel‐hydrogel. As shown in Figure 2f, the rapid decay of the DM‑BODIPY absorption (450–550 nm) within 20 min confirmed efficient photolytic activation. Correspondingly, the telmisartan‐specific absorption (270–350 nm) remained unchanged during illumination, indicating that drug release did not occur in this phase. When the hydrogel was subsequently incubated in the dark, the telmisartan signal gradually declined over 10 days—a direct reflection of sustained hydrolytic drug release from the unlocked network. Together, these results verified that the GTel‑hydrogel system successfully retained the two‑stage release kinetics of rapid photolysis followed by prolonged hydrolysis. Notably, HPLC analysis further validated this delayed drug release (Figure 2g). A distinct peak matching the retention time of the telmisartan standard appeared in the 24‐hour post‐irradiation chromatogram, definitively identifying the released molecule. The complete release profiles further quantified this sustained phase (Figure 2h). PDMA‐GTel10 released 44.4% ± 1.2% within 12 h and 64.0% ± 0.3% by day 1, reaching 97.7% ± 2.3% after 10 days.

The storage stability of the PDMA‐GTel hydrogel was assessed under dark conditions. Over a 10‑day period, HPLC analysis showed that cumulative drug leakage from the hydrogel remained below 20% of the total loaded amount (Figure 2h). This high retention confirms its stability in the absence of light and supports its potential as a long‑term implantable depot.

### 2.4 Programmability of Phototriggered Drug Release Behavior of the GTel‐Hydrogel

For programmable drug delivery, precise dose control at the level of individual phototriggering events is essential. The amount of drug released in response to a single phototrigger mode from PDMA‐GTel10 hydrogel was precisely programmed by modulating the illumination parameters, specifically either the exposure time or the light power. As an example, 20 s of LED irradiation (50 mW cm^−2^) triggered the release of about one‑third of the total drug load over 48 h, whereas reducing the exposure to 5 s decreased the output to approximately one‑seventh (Figure 2i). Similarly, 30 s of laser irradiation at 50 mW cm^−2^ yielded a comparable release fraction, while lowering the power to 2 mW cm^−2^ for the same duration limited the release to only one‑tenth of the total load (Figure 2j). Furthermore, each irradiation triggered a defined release cycle. This cycle consisted of a rapid photolytic initiation and a self‐sustaining hydrolysis‐driven release, which was largely complete within 2 days, followed by a return to a near‐quiescent baseline state. In this stable state, the GTel‑hydrogel system exhibited only minimal passive leakage (∼3% per day). These results demonstrate that the total drug released following a phototrigger can be accurately preset by defining the light dose. Additional calibration experiments (Figure S27) confirmed a quantitative dose‑response relationship between light dose and drug release from the PDMA‑GTel20 hydrogel, with cumulative release increasing progressively with both illumination time (5–300 s at 35 mW cm^−2^) and intensity (5–50 mW cm^−2^ for 1 min).

The system further demonstrated repeatable, multi‑trigger release capability. Using the same PDMA‐GTel10 hydrogel, three sequential phototriggering events were applied on days 0, 2, and 4, each consisting of 5 s of LED irradiation at 50 mW cm^−2^. After each trigger, approximately 14% of the total drug load was steadily released over the next 48 h, with release dropping below 5% between 24 and 48 h (Figure 2k). This reproducible phototriggered release behavior across multiple independent cycles verified that the hydrogel could enable on‑demand and programmable drug administration. This multi‐trigger functionality is fundamental for an implantable depot to match the evolving therapeutic windows of conditions like MI.

In summary, we developed a GTel‑based hydrogel platform that operated via a phototriggered, sustained‐release mechanism. This platform combines high retention stability with programmable control over drug loading (via monomer feed ratio) and release dose per trigger (via illumination parameters), and supports multiple on‑demand release cycles. Together, these programmable features provide a robust material foundation for constructing implantable, actively programmable multi‑trigger drug delivery devices such as the iPDP, which could adapt therapy in response to dynamic disease progression.

### 2.5 Fabrication and Characterization of the iPDP Device

**FIGURE 3:**
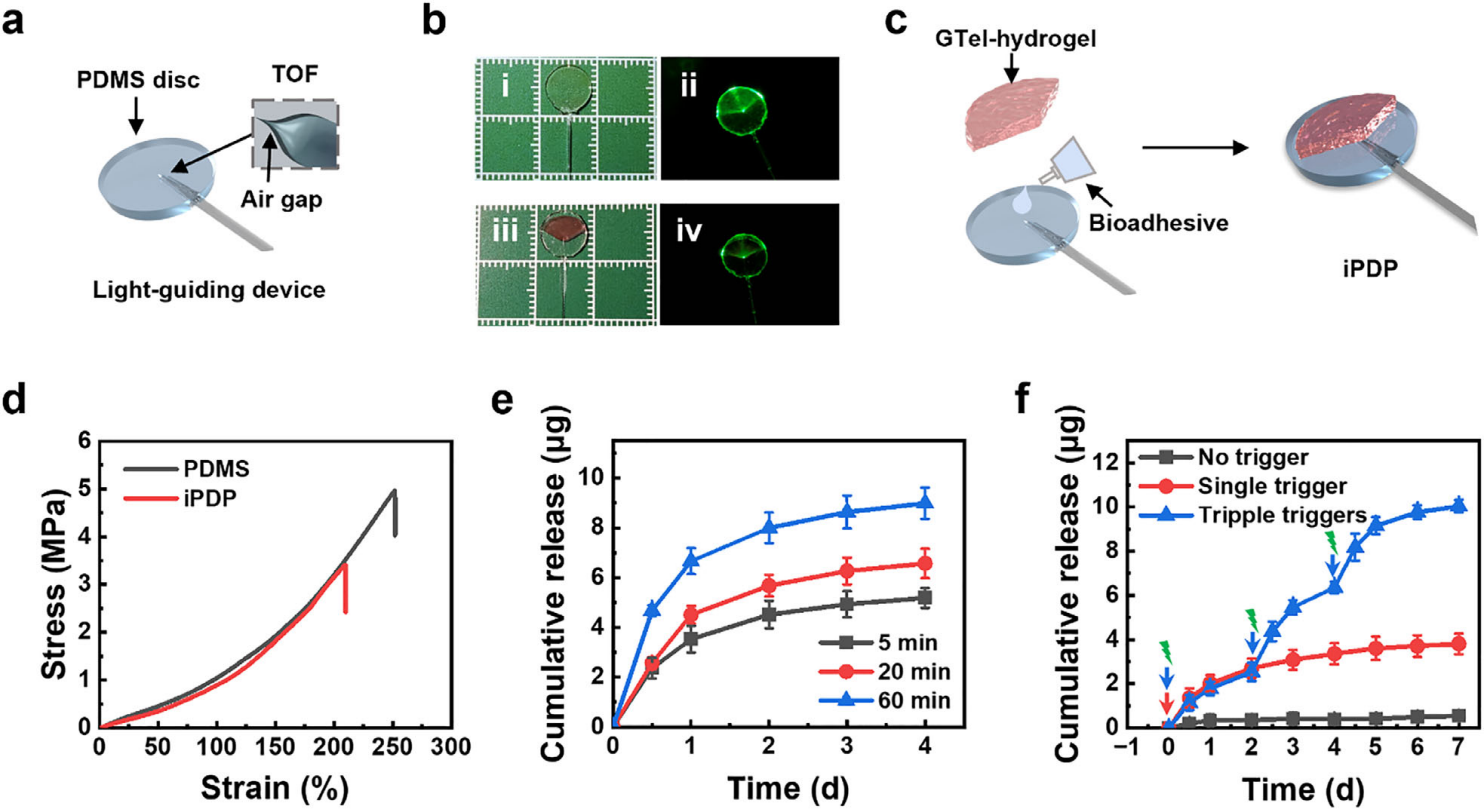
Integrated active programmable phototriggered release and mechanical characterization of the iPDP device. (a) Schematic illustration of the flexible light‐guiding device, comprising a PDMS disc embedded with a tapered optical fiber (TOF). A deliberate air gap is present between the TOF tip and the PDMS matrix. (b) Photographs of key fabrication stages, including (i) the light‑guiding device prior to hydrogel integration, (ii) the light‑guiding device in operation upon laser connection, (iii) the fully assembled iPDP, and (iv) the illuminated iPDP with light emitted through the hydrogel. (c) Schematic illustration of the iPDP assembly process. (d) Tensile stress–strain curves of PDMS alone and the complete iPDP. Tests were performed at 25°C using dumbbell‑shaped specimens (gauge length *L*_0_ = 10 mm, width of narrow section *W* = 2 mm). For PDMS alone, the specimen thickness was 1 mm. For the iPDP, the thickness was approximately 0.67 mm, matching the actual device (PDMS disc + swollen hydrogel layer). The crosshead speed was 10 mm min^−1^. Representative curves are shown. (e) Cumulative drug release profiles from the iPDP following single‑trigger laser illumination (35 mW cm^−2^) for different durations. (f) Drug release profiles of the iPDP under different triggering conditions: no trigger (baseline), a single trigger at Day 0, and triple triggers at Days 0, 2, and 4. The parameters were as follows: for the single trigger, 10 mW cm^−2^ for 1 min; for the triple triggers, 10 mW cm^−2^ for 1 min on Day 0, and 35 mW cm^−2^ for 5 and 20 min on Days 2 and 4, respectively. Arrows with lightning symbols mark the timing of each phototriggering event. Data are presented as mean ± SD (*n* = 3).

The biocompatible light‐guiding device was first designed and fabricated, with its structure and optical characteristics established for cardiac drug‐delivery applications. It consisted of a polydimethylsiloxane (PDMS) disc (radius: 4.06 ± 0.04 mm, thickness: 404 ± 7 µm) with an embedded tapered optical fiber (TOF) (Figure 3a). A key design feature, adapted from a previously reported luminescent cardiac patch [27], was the intentional introduction of a ∼20 µm air gap at the TOF tip within the PDMS matrix. This structure exploited the refractive index contrast between PDMS (*n*_PDMS_ ≈ 1.41) and air (*n*_air_ = 1) to induce wide‐angle (130.4° ± 1.3°) light scattering from the point source. As a result, the fiber output is transformed into a planar emission pattern suitable for illuminating the overlying hydrogel (Figure 3b‐i,ii).

To maximize light utilization, the hydrogel geometry needed to match the emission profile of the device. When a circular hydrogel was integrated with the device, illumination produced a fan‑shaped light field with a central angle of 126.9° ± 1°—consistent with the intended wide‑angle scattering design (Figure S28a). Regional PL emission spectroscopy was employed to directly compare the fan‐shaped irradiated zone with adjacent non‐irradiated areas of the hydrogel. The analysis showed that the photolytic reaction was highly efficient within the illuminated area but spatially restricted, with the prodrug remaining virtually intact outside the target zone (Figure S28b). Based on this profile, the prodrug‐loaded hydrogel was precision‐cut into a matching sector shape (radius: 4.33 ± 0.05 mm, central angle: 118.0° ± 0.4°) and bonded to the optical device, yielding the complete iPDP device (Figure 3c,b‐iii,iv). This geometry‐optimized design ensured that the prodrug‐containing hydrogel almost entirely occupied the effective light field, thereby compensating for the constrained illumination area. Quantitatively, under identical localized illumination, the fan‐shaped hydrogel achieved near‑complete cumulative drug release of 89% ± 6%, markedly surpassing the 50% ± 3% release obtained from a conventional circular design (Figure S28c). The sector‑shaped PDMA‑GTel20 hydrogel was used for device integration and all subsequent in vivo studies. The 20% prodrug monomer feed ratio was selected to compensate for the volume reduction (∼30%) of the sector‑shaped hydrogel compared to the original circular design, ensuring that the final iPDP achieved a therapeutically relevant payload (∼10 µg per device). Importantly, the release kinetics of GTel20 are similar to those of GTel10 (both release >70% within 48 h, difference <10%; Figure S29), confirming that the programmable release behavior established with GTel10 is transferable to the GTel20 formulation.

To ensure practical implantation, the iPDP device exhibited robust and compliant mechanical properties (Figure 3d; Figure S30, Table S2). The overall device dimensions were chosen to match the scale of a rat's left ventricle, with a disc radius of about 4 mm. The PDMA‐GTel hydrogel reached a thickness of 273 ± 6 µm at swelling equilibrium, keeping the total device thickness below 700 µm, thereby achieving a thin profile intended to minimize mechanical interference with cardiac motion. The hydrogel itself showed moderate tensile strength and modulus, while the PDMS disc substrate offered higher toughness but maintained a relatively low modulus. This combination ensured device integrity during handling and suturing, while allowing flexible, conformal contact with the beating heart. For optical input, the integrated optical fiber of the iPDP (∼40 cm in length) was readily connected to an external 520 nm laser via a standard fiber optic connector, thereby establishing a percutaneously accessible optical pathway for external control (Figure S31).

### 2.6 Programmable Multi‐Triggered Drug Release Performance of the iPDP Device

The programmable drug‑release performance of the iPDP was first evaluated under single‑trigger conditions. Devices were connected via optical fiber to a 520 nm laser and illuminated at a fixed irradiance of 35 mW cm^−2^ for 5, 20, or 60 min. The cumulative drug release over 48 h increased non‑linearly with illumination time, reaching 4.5 ± 0.5, 5.7 ± 0.4, and 8 ± 0.6 µg, respectively (Figure 3e). After this active release phase, all groups settled into a low‑rate plateau, with daily release dropping below 5% after 48 h. This reproducible transition to a near‐quiescent state after each release phase confirms the reliable “on‐off” release kinetics of the iPDP device, a critical feature for enabling on‐demand, discrete drug dosing.

To see whether the device could support a clinically relevant schedule, a single iPDP device was programmed to execute three scheduled drug releases mimicking a post‑infarction regimen. The total drug payload (10.7 ± 0.9 µg) was distributed for administration on days 0, 2, and 4, with a target dose of 2–4 µg per release cycle. The illumination parameters for each dose were chosen based on the established correlation between illumination settings and single‑trigger release output (Figure 3e). A dedicated experiment confirmed that 10 mW cm^−2^ for 1 min releases 2.7 ± 0.4 µg (Figure 3f), meeting the target per‑cycle dose of 2–4 µg for the first trigger. For the second trigger, a longer exposure time and higher illumination intensity (5 min at 35 mW cm^−2^) were applied, which released 3.8 ± 0.3 µg of drug. For the third trigger on day 4, to ensure complete drug release, we used 35 mW cm^−2^ for 20 min, resulting in a release of 3.7 ± 0.3 µg. The cumulative release after three triggers reached approximately 95% of the total payload. As shown in Figure 3f, the programmed regimen yielded three distinct release events, with measured drug amounts of 2.5 ± 0.4, 3.8 ± 0.3, and 3.7 ± 0.3 µg on the respective days. This successful demonstration of multi‑cycle, on‑demand dosing confirms that the iPDP can execute complex release schedules. In addition, the device showed excellent storage stability, with cumulative drug leakage below 5% after 7 days under light‑shielded conditions (Figure 3f). Release tests under more physiologically relevant culture medium confirmed that the programmable release behavior was comparable to that in 0.5% Tween (Figure S32).

To compare the release behavior of the hydrogel alone and the integrated iPDP device, we performed release experiments on the PDMA‑GTel20 hydrogel using the same illumination conditions as for the device (10 mW cm^−2^ for 1 min and 35 mW cm^−2^ for 5 min). Within 48 h, the hydrogel alone released 37.3% ± 2.4% and 82.4% ± 3.2% of its total drug load under these two conditions, while the iPDP device released 25.2% ± 4.1% and 42.2% ± 5.1%, respectively (Figure S33). The lower release from the device is attributed to its inhomogeneous light field, but the dose‑dependent trend remains consistent. These results confirm that the programmable release mechanism established with the hydrogel is retained in the device, albeit with quantitatively different output due to the distinct light delivery mode.

Based on the above results, the iPDP realizes the multidimensional programmability of the GTel‑hydrogel as a fully integrated, implantable system. It builds upon the intrinsic phototriggered release mechanism and the stability conferred by the covalent linkage of the prodrug, which enables pulsed release characterized by an active phase and a low‑leakage quiescent state. Crucially, the iPDP facilitates the programming of complex release schedules through simple adjustment of external laser parameters via its integrated optical fiber, while retaining the inherent multidimensional tunability of the hydrogel. This indicates the iPDP as an implantable prodrug depot capable of active, on‐demand drug release. Thereby, the iPDP translates molecular‑level photoresponse into an implant‑level programmable platform, which offers a potential strategy for on‑demand, multi‑cycle drug administration in MI therapy.

### 2.7 Biosafety of the iPDP Device

**FIGURE 4:**
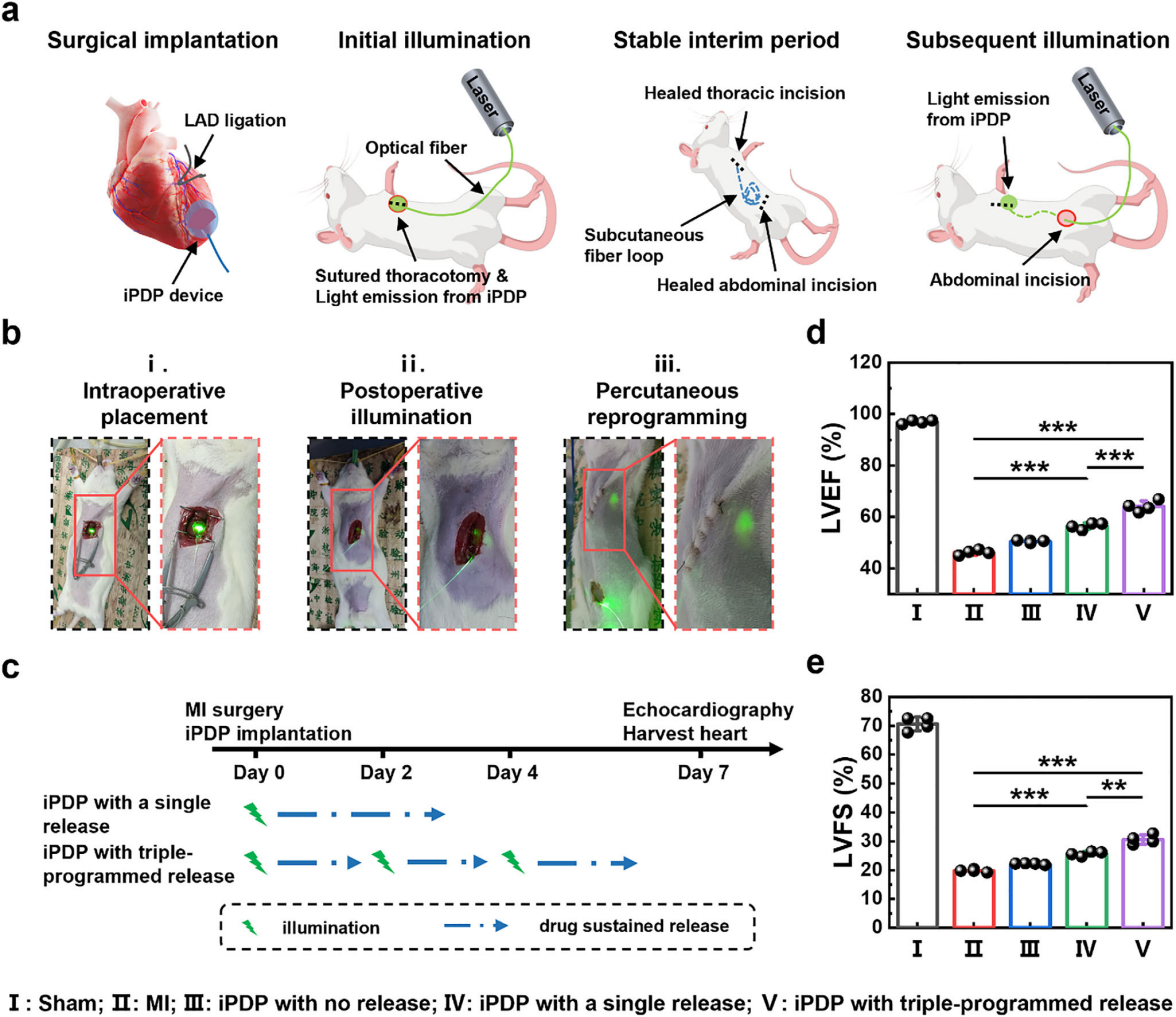
In vivo therapeutic efficacy of the iPDP device for post‐MI treatment. (a) Schematic diagram of illumination implantation operation of the iPDP device. (b) Photographs of the in vivo illumination procedure with the iPDP device, including (i) device placement on the exposed heart during implantation, (ii) first percutaneous illumination after surgery, (iii) second illumination via the subcutaneous fiber accessed from the abdomen, without repeat thoracotomy. (c) Schematic timeline of the animal study. The bottom panels conceptually illustrate the contrasting drug release strategies for groups IV (single release) and V (triple‐programmed release). (d) Left ventricular ejection fraction (LVEF) and (e) left ventricular fractional shortening (LVFS) evaluated by echocardiography at 7 days post‐implantation. Data are presented as mean ± SD (*n *= 4). **p < 0.01, ***p < 0.001. I: Sham group; II: MI group; III: iPDP with no release group; IV: iPDP with a single release group; V: iPDP with triple‐programmed release group.

For in vivo programmability and stability of the iPDP device, the device was first successfully implanted and secured onto the epicardial surface, with immediate light emission confirming both its placement and functional integrity (Figure 4a,b‐i). The first percutaneous illumination was successfully administered postoperatively (Figure 4a,b‐ii). Crucially, repeated programming phototriggering was achieved in the same animal via the pre‑implanted subcutaneous optical fiber on postoperative day 2 without additional surgery, demonstrating the feasibility of percutaneous multi‑cycle dosing (Figure 4a,b‐iii).

The safety profile of the iPDP was first assessed in vitro by co‑culturing the rat cardiomyocyte cell line (H9c2) with iPDP devices for 72 h in a Transwell system. CCK‑8 assays showed that cell viability remained above 90% for all GTel10‑based experimental groups (Figure S34) and also for the GTel20‑based iPDP device with or without light irradiation (Figure S35), confirming that the device is biocompatible and suitable for cardiac implantation.

To evaluate systemic safety after long‑term implantation, we performed comprehensive hematological, serum biochemical, and histological analyses. As shown in Figure S36, key hematological parameters, including white blood cell, neutrophil, lymphocyte, red blood cell, hemoglobin, and platelet counts, showed no statistically significant differences between the iPDP‑treated and sham‑operated groups on day 7 post‑operation. Liver function markers (aspartate aminotransferase, alanine aminotransferase, and their ratio), kidney function markers (urea and creatinine), and the albumin‐to‐globulin ratio were also comparable across groups (Figure S37). Hematoxylin and eosin (H&E) staining of major organs revealed no pathological damage in the iPDP group relative to the sham group (Figure S38). Together, these results indicate that the iPDP device and its programmable release regimen do not induce detectable systemic toxicity beyond the surgical procedure itself, supporting its biocompatibility and preliminary safety profile.

Serum glutathione (GSH) levels were measured on day 7. No significant difference was observed between the sham and iPDP‑treated groups, confirming the absence of light‑induced oxidative stress (Figure S39). Based on stoichiometric calculations (1:1 molar ratio between telmisartan and each byproduct), the estimated daily exposure to DM‑BODIPY‑OH and 2,6‑bis(hydroxymethyl)aniline is <0.8 and <0.5 µg, respectively, which are extremely low amounts and are not expected to pose any systemic toxicity risk.

### 2.8 Therapeutic Efficacy of the iPDP Device in a Rat MI Model

To assess therapeutic efficacy in vivo, a rat MI model was established by permanent ligation of the left anterior descending (LAD) coronary artery. The total telmisartan payload of the iPDP device was designed with reference to an efficacious oral dose of 10 mg^−1 ^kg^−1 ^day^−1^ [42]. Based on the rat heart‑to‑body weight ratio (∼0.38%), only about 7.6 µg of the daily oral dose may reach the cardiac tissue, establishing a local target in the low‑microgram range. The final iPDP device delivered a total payload of 10.7 ± 0.9 µg per device. The rats were randomly assigned to one of five distinct therapeutic groups: the sham group, defined as rats that underwent thoracotomy without LAD coronary artery ligation; the MI group, defined as rats that underwent ligation without treatment; the iPDP with no release group, defined as MI rats implanted with the iPDP device but not subjected to illumination; the iPDP with a single release group, defined as MI rats implanted with the iPDP device and immediately exposed to a single illumination; and the iPDP with triple‐programmed release group, defined as MI rats implanted with the iPDP device and treated with triple fractionated illumination on postoperative days 0, 2, and 4. For the two photo‑activated groups, total telmisartan release was kept at approximately 10 µg, allowing direct comparison of different intervention strategies under equivalent dosage conditions. Figure 4c illustrates the overall treatment regimen.

**FIGURE 5:**
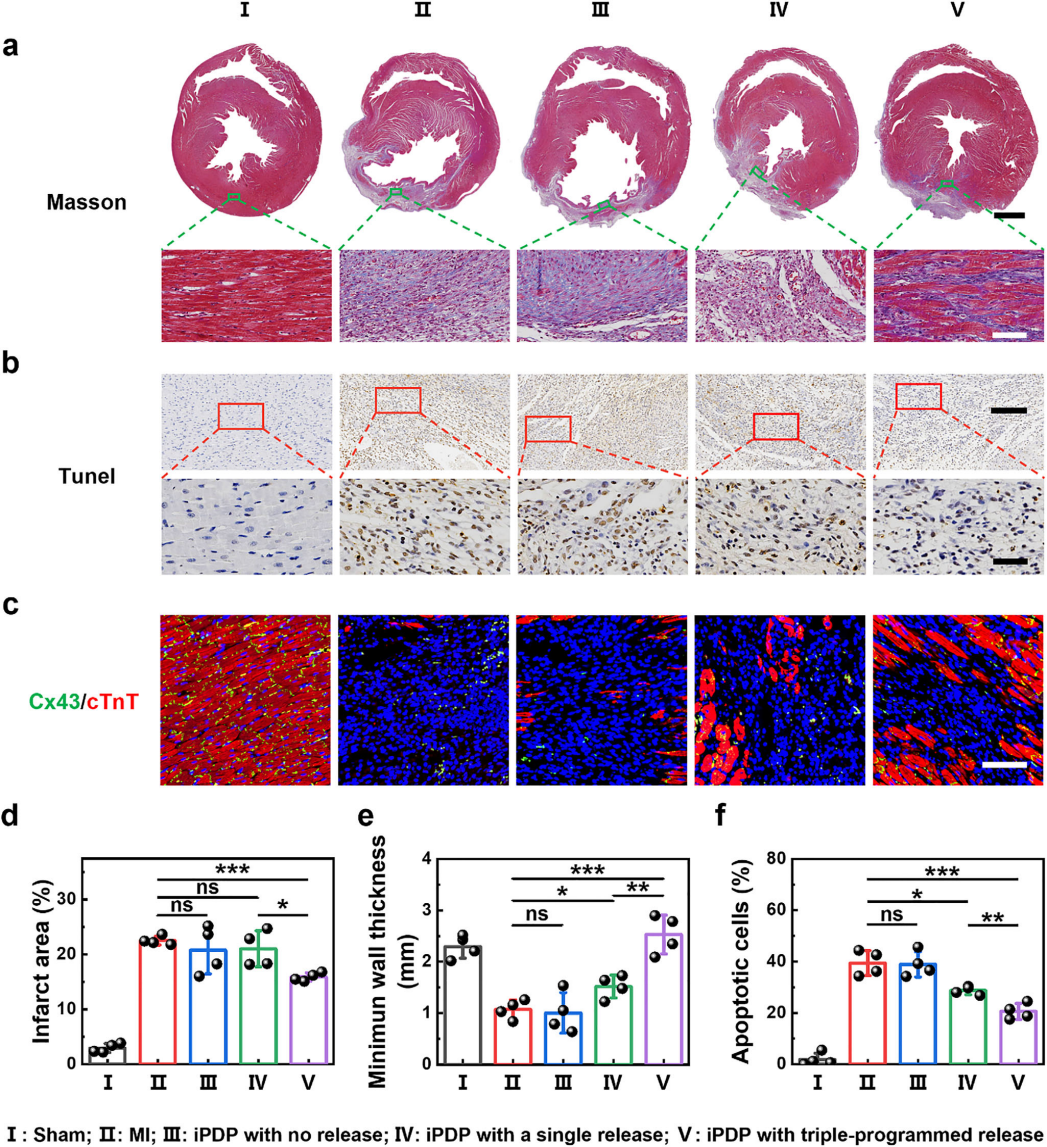
Multi‑parametric assessment of cardiac repair 7 days post‐MI. (a) Representative Masson's trichrome‐stained myocardial sections in various treatment groups (scale bars: black = 2 mm, white = 100 µm). (b) Representative TUNEL staining images of heart sections. Scale bars: upper panel = 200 µm, lower panel = 50 µm. (c) Representative immunofluorescence staining for Cx43 (green), cTnT (red), and nuclei (blue). Scale bar = 100 µm. Quantification of (d) infarct area and (e) minimum left ventricular wall thickness. (f) Quantitative analysis of the percentage of TUNEL^+^ apoptotic cells. Data are presented as mean ± SD (*n* = 4). *p < 0.05, **p < 0.01, ***p < 0.001, ns, not significant. I: Sham group; II: MI group; III: iPDP with no release group; IV: iPDP with a single release group; V: iPDP with triple‐programmed release group.

Echocardiographic assessment at 7 days post‑MI showed that both the single‑release and triple‑release groups improved cardiac function over the untreated MI group. Notably, the triple‐programmed release regimen led to significantly greater improvements in both LV ejection fraction (LVEF) and fractional shortening (LVFS) than the single release, highlighting the benefit of active programmable drug release (Figure 4d,e; Figure S40). Cardiac structural remodeling was evaluated by Masson's trichrome staining, as indicated by infarct size and LV wall thickness. While histological sections showed a trend toward preserved tissue architecture in both iPDP‐treated groups (Figure 5a), quantitative analysis revealed that only the triple‐programmed release group achieved a statistically significant reduction in infarct size relative to MI controls (Figure 5d). The single release group showed no statistically significant improvement. Furthermore, the triple‐programmed release group achieved significantly thicker minimum LV wall than the single release group (Figure 5e). This supports that repeated dosing is necessary to counteract the evolving pathology, as a single dose proved insufficient. Hence, the programmable, multi‐cycle release capability of the iPDP is essential for achieving therapeutic efficacy.

Cardiac cell apoptosis was assessed by TdT‐mediated dUTP Nick‐End Labeling (TUNEL) staining (Figure 5b,f). Compared to the Sham group, the MI group exhibited a significant increase in apoptotic cells, confirming substantial ischemic injury. Both the single‐ and triple‐release groups significantly reduced the extent of apoptosis relative to the MI group, with the triple‐programmed release regimen showing better efficacy in further reducing the apoptotic cell percentage. Furthermore, qualitative immunofluorescence observation suggested better preservation of myocardial tissue, as evidenced by more extensive and continuous areas of cardiac troponin T (cTnT)‐positive cardiomyocytes, accompanied by more organized localization of the gap junction protein connexin 43 (Cx43) within these areas, in the triple‐programmed release group compared to MI or single‐release groups (Figure 5c).

### 2.9 Transcriptomic Effects of the Actively Programmed Release Strategy of the iPDP Device in MI

**FIGURE 6:**
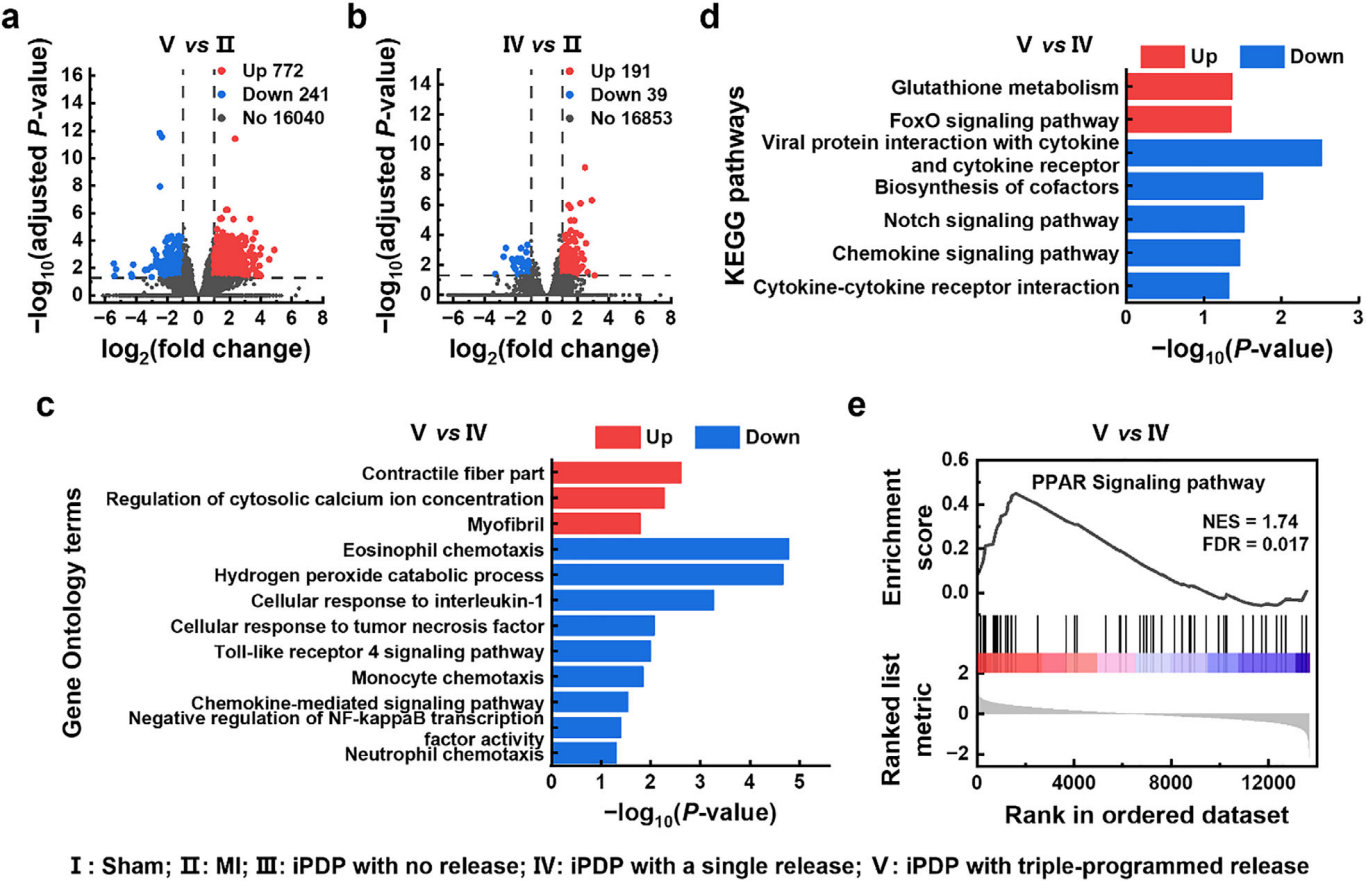
Transcriptomic profiling of infarcted heart tissues 7 days post‐MI. (a,b) Volcano plots visualizing DEGs. Genes with an adjusted *P*‐value < 0.05 and |log_2_(fold change)| > 1 are highlighted in red (upregulated) or blue (downregulated). (a) DEGs between group V and group II. (b) DEGs between group IV and group II. (c) Gene Ontology and (d) Kyoto Encyclopedia of Genes and Genomes (KEGG) pathway enrichment analysis of DEGs identified from the comparison between groups V and IV. Significantly enriched terms (*P*‐value < 0.05) are displayed. (e) Gene set enrichment analysis (GSEA) plot for the KEGG PPAR signaling pathway, comparing group V versus group IV. The positive normalized enrichment score (NES = 1.74) and false discovery rate (FDR = 0.017) indicate significant upregulation of this pathway in group V. *n* = 3. I: Sham group; II: MI group; III: iPDP with no release group; IV: iPDP with a single release group; V: iPDP with triple‐programmed release group.

To elucidate the molecular basis underlying the differential therapeutic efficacy of the dosing strategies, RNA sequencing analysis was performed on heart tissues of the infarct regions at 7 days post‐MI. Principal component analysis and hierarchical clustering revealed distinct transcriptomic separation among groups (Figure S41), with the triple‐release group (V) clustering closer to the Sham group (I), indicating that the programmed dosing strategy more effectively shifted the cardiac transcriptome toward a homeostatic profile. Volcano plot analysis showed that the triple‐programmed release induced extensive and specific transcriptomic reprogramming, as evidenced by 1013 differentially expressed genes (DEGs) compared to the MI group. In stark contrast, single release elicited only a minimal transcriptional response, with a mere 230 DEGs (Figure 6a,b).

Comparison of each treatment group against the MI group highlighted the superiority of programmed release (Figures S42,S43). While single release modestly upregulated some metabolic pathways (e.g., fatty acid oxidation) and downregulated a limited set of immune pathways, triple‐release elicited a far more comprehensive and robust transcriptional reprogramming. It strongly activated energy metabolism pathways (e.g., oxidative phosphorylation, TCA cycle) and cardiac structure genes (e.g., myofibril assembly, cardiac muscle contraction), while profoundly suppressing multiple inflammatory and pro‐fibrotic signaling axes, including the NF‐kappa B signaling pathway, IL‐17 signaling pathway, NOD‐like receptor signaling pathway, and critically, the renin‐angiotensin system (RAAS) (Figure S43b). The coordinated anti‐inflammatory and metabolic modulation observed above closely aligns with the known pharmacology of telmisartan as a partial PPARγ agonist [48], a pathway also enriched in both drug release groups.

To further delineate the molecular events specific to the programmed regimen, we focused on the direct comparison between triple and single release (V vs. IV). Gene Ontology (GO) enrichment analysis revealed that triple‐programmed release significantly downregulated a broad spectrum of pathways that directly initiate and propagate the inflammatory response (Figure 6c), including cellular responses to key cytokines (cellular response to interleukin‐1, cellular response to tumor necrosis factor), innate immune receptor signaling (toll‐like receptor 4 signaling pathway), and key steps in leukocyte recruitment (monocyte chemotaxis, neutrophil chemotaxis, eosinophil chemotaxis, chemokine‐mediated signaling pathway). Concurrently, triple‐release specifically upregulated pathways involved in cardiomyocyte structure and function maintenance, including contractile fiber part, myofibril, and regulation of cytosolic calcium ion concentration. Kyoto Encyclopedia of Genes and Genomes (KEGG) pathway analysis reinforced these findings (Figure 6d). The triple‐release group demonstrated pronounced downregulation of core inflammatory communication pathways, namely the chemokine signaling pathway and cytokine–cytokine receptor interaction, as well as the Notch signaling pathway, which has been implicated in fibroblast activation. Meanwhile, upregulation of glutathione metabolism and FoxO signaling pathway aligned with enhanced cellular antioxidant defense and survival capacity. To compare the activation of the PPAR signaling pathway between single release and triple‐programmed release, we performed a gene set enrichment analysis (GSEA) and observed higher activation in triple‐programmed release (Figure 6e), indicating that programmed delivery achieves more sustained and robust activation of this core pharmacological target.

### 2.10 In Vivo Validation of Anti‐Inflammatory Effects

**FIGURE 7:**
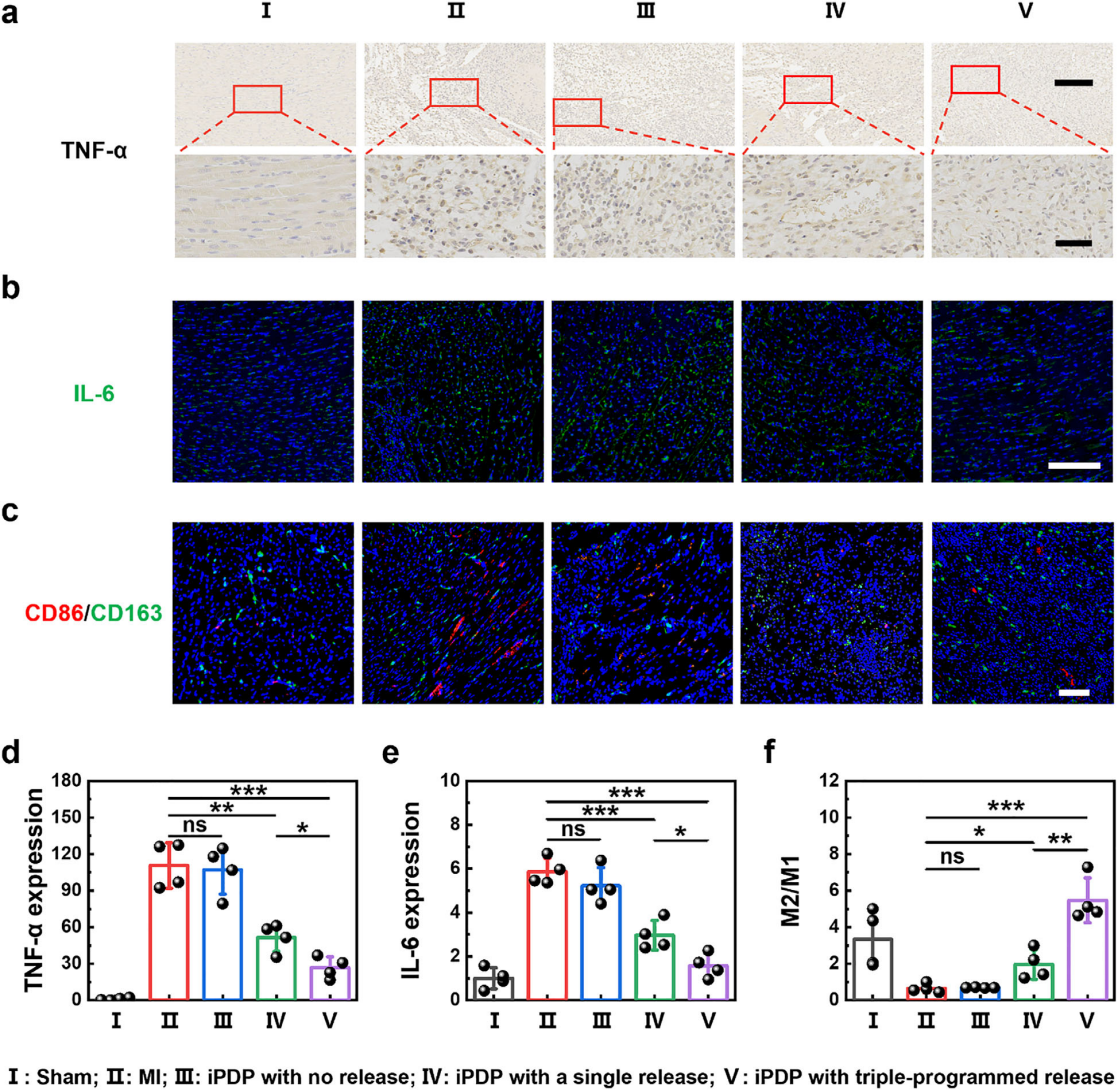
Analysis of inflammatory response and macrophage polarization 7 days post‑MI (a) Representative immunohistochemical staining for TNF‐α. Scale bars: upper panel = 200 µm, lower panel = 50 µm. (b) Representative immunofluorescence staining for IL‐6 (green) and nuclei (blue). Scale bar = 200 µm. (c) Representative immunofluorescence triple‐staining for macrophages: M1 phenotype (CD86^+^, red), M2 phenotype (CD163^+^, green), and nuclei (blue). Scale bar = 100 µm. (d,e) Semi‐quantitative analysis of (a) and (b), respectively, showing the relative expression levels of (d) TNF‐α and (e) IL‐6, normalized to the Sham group (I). (f) Quantitative analysis of the M2/M1 macrophage ratio. Data are presented as mean  ± SD (*n* = 4). *p < 0.05, **p < 0.01, ***p < 0.001, ns, not significant. I: Sham group; II: MI group; III: iPDP with no release group; IV: iPDP with a single release group; V: iPDP with triple‐programmed release group.

To validate the anti‐inflammatory effects suggested by the transcriptomic analysis, we examined key inflammatory markers at the protein level. iPDP treatment significantly modulated key pro‑inflammatory cytokines, tumor necrosis factor‐α (TNF‐α), and interleukin‐6 (IL‐6). As shown in Figure 7a, the TNF‑α signal within the infarction area was markedly reduced in the iPDP with triple‐programmed release group compared to all other groups. Quantitatively, TNF‑α expression in this group dropped to 24% of MI levels and was significantly lower than in the single release group (Figure 7d). Similarly, the triple‐programmed release group showed only a 1.6‑fold increase in IL‑6 levels over the Sham group. This was markedly lower than the threefold increase in the single release group and the 5.9‑fold increase in the MI group (Figure 7b,e). Macrophage polarization provided further mechanistic insight. The M2/M1 ratio, a key indicator of the shift from inflammation to repair, was highest in the triple‐programmed release group (5.5 ± 1.2), significantly elevated compared to the single release (2.0 ± 0.8) and MI (0.65 ± 0.24) groups (Figure 7c,f; Figure S44). These effects demonstrate the advantage of the prolonged, multi‑dose drug‑release profile achievable through programmed multi‐dose delivery. Unlike the single‑release regimen with its limited duration, the triple‑release strategy sustained therapeutic coverage over the critical 7‑day post‑MI period. This extended pharmacological presence more effectively maintains an anti‑inflammatory microenvironment and avoids the compensatory pro‑inflammatory activation seen after a single high‑dose treatment. Consequently, the active, programmable release via the iPDP achieves more profound and sustained suppression of the pro‑inflammatory cascade while fostering a repair‑conducive immune phenotype. This spatiotemporally coordinated immunomodulation is a central mechanism by which multi‑cycle iPDP therapy mitigates post‑infarction inflammation more effectively than a single‑release treatment.

### 2.11 In Vitro Validation of Anti‐Fibrotic Effects

Telmisartan is known to exert anti‐fibrotic effects by blocking the angiotensin II receptor [40, 41, 42]. In our study, Masson staining revealed reduced fibrosis after iPDP implantation, and transcriptomic analysis further demonstrated downregulation of pro‐fibrotic pathways, including Notch signaling and the RAAS, in the triple‐release group. To test the effect of triggerable release in inhibiting two key profibrotic phenotypes in fibroblasts, namely excessive proliferation and migration, an in vitro Transwell co‑culture experiment was performed. The light‐activated iPDP showed a clear dose‑dependent inhibition of fibroblast proliferation across a range of initial drug loadings, with the effect plateauing at higher doses where no significant difference was observed between the 44 and 22 µg groups (Figure S45a). This dose‑response profile closely matched that of equivalent concentrations of free telmisartan (Figure S45b). Control groups, including non‑irradiated devices, blank hydrogels, and cells alone, showed negligible effects, confirming the specificity of photo‑triggered drug release. Furthermore, the light‐activated iPDP also significantly suppressed fibroblast migration to a level comparable with free telmisartan, whereas the non‑irradiated device showed no such effect (Figure S46).

These in vitro results demonstrate that telmisartan released from the iPDP retains its intrinsic pharmacological activity to directly suppress fibroblast proliferation and migration. This provides a mechanistic basis for the anti‐fibrotic effects observed in vivo, complementing the inflammation‐resolving actions of the programmed release strategy.

## 3 Discussion

The iPDP integrates a phototriggered prodrug hydrogel with a percutaneous light‐guiding device to achieve actively programmable drug release in deep tissues. The prodrug is engineered with a self‐immolative linker that undergoes rapid photolysis upon brief illumination, followed by sustained hydrolysis that releases the active drug over days. This two‑stage release profile offers a distinct advantage over conventional phototriggered systems: a single brief light pulse initiates an autonomous, sustained release program (∼48 h), eliminating the need for continuous illumination and avoiding an initial burst. Direct epicardial delivery of 10.7 µg achieves local drug concentrations in the low‑micromolar range, consistent with the in vitro EC_50_ of telmisartan (∼4.5 µm) for PPARγ activation [48]. Sequential triggers on days 0, 2, and 4 thereby maintain therapeutic coverage over the 7‑day study period, as illustrated in Scheme 1b. This cascade decouples the optical trigger from drug release, enabling each brief light input to initiate a complete, autonomous release program. At the device level, this mechanism translates into two key programmable features: dose per trigger controlled by illumination parameters, and multiple on‑demand release cycles from a single implant. The integrated light guide provides a percutaneous interface for post‑implantation programming without repeat surgery. These design features establish iPDP as a directly addressable depot capable of adapting drug delivery to evolving clinical needs.

The therapeutic superiority of the triple‑programmed release regimen was firmly established by comprehensive in vivo assessments. Compared with single‑dose delivery, fractionated dosing significantly improved cardiac function and structure, as evidenced by elevated LVEF, reduced infarct size, attenuated cardiomyocyte apoptosis, and preserved gap junction organization. Transcriptomic analysis revealed that these functional benefits originated from coordinated modulation of multiple pathological pathways: sustained activation of PPAR signaling drove broad‑spectrum anti‑inflammatory effects via suppression of the NF‑κB signaling pathway, while concurrent inhibition of the RAAS provided a direct anti‑fibrotic mechanism. These transcriptional changes translated into tangible improvements in the myocardial microenvironment, as confirmed by reduced pro‑inflammatory cytokines (TNF‑α, IL6) and an elevated M2/M1 macrophage ratio in vivo, as well as by the direct suppression of fibroblast proliferation and migration in vitro. Thus, the actively programmed regimen does not merely prolong drug exposure but fundamentally reshapes the post‑infarct healing trajectory—suppressing pathological inflammation, mitigating fibrosis, and enabling endogenous repair in a temporally coordinated manner—a multi‑target modulation unattainable with a single static dose. These transcriptomic findings are directly consistent with the established dual pharmacology of telmisartan (PPARγ activation and RAAS inhibition) [48]. Moreover, the observed differences between single and triple release suggest that the fractionated regimen provides more sustained anti‑inflammatory coverage during the acute phase and better promotes reparative pathways during the subacute repair phase.

The translational potential of the iPDP platform is supported by its modular design and use of clinically compatible materials (PDMS, PDMA‑based hydrogels, visible light). PDMA was chosen for its high hydrophilicity, which is beneficial for mitigating adhesion, a common complication associated with cardiac patches. Although the mechanical properties of the PDMS disc (modulus ∼1 MPa) are much higher than those of native myocardium (epicardial modulus 30–70 kPa [49]), the thin device architecture (<700 µm) and minimal suture fixation (two sutures at the disc edge) allow conformal contact and reduce stress transmission, consistent with the absence of detectable tissue damage or functional impairment in our 7‑day in vivo study. Future iterations could adopt softer elastomers (e.g., SEBS or soft silicone) with modulus closer to cardiac tissue to further improve mechanical compatibility.

Beyond the specific application demonstrated here, the modular design of the iPDP platform offers significant versatility for broader therapeutic applications. The sustained release rate can be rationally tuned by varying the hydrophilicity of the primary monomer, as evidenced by the library of hydrogels with distinct release kinetics (detailed in Figures S47–S50). This tunability provides a straightforward handle to match the release profile to the desired treatment duration for different indications. Furthermore, the phototriggered prodrug chemistry is not limited to telmisartan; it can be adapted to other therapeutic agents bearing suitable functional groups (e.g., carboxylic acids, amines), enabling the platform to target diverse chronic conditions requiring sustained local therapy. The adaptability of the prodrug platform to other therapeutic agents is supported by the structural versatility of the 2,6‑bis(hydroxymethyl)aniline self‑immolative spacer [38, 47]. Drugs containing a carboxyl group can be conjugated via an ester bond (as demonstrated with telmisartan), while drugs containing a primary amine are compatible via a carbamate linkage (e.g., mexiletine). The release kinetics are governed by hydrolysis of the ester or carbamate bond, which is influenced by structural factors such as steric hindrance, electronic effects, and drug hydrophobicity—with more hydrophobic drugs exhibiting slower hydrolysis due to restricted water access [50, 51, 52]. These structure‑release kinetics relationships enable the selection of appropriate drug payloads to match the desired therapeutic time course—fast release for acute conditions such as antiarrhythmic intervention, or sustained release for chronic conditions such as post‑MI remodeling. This inherent modularity positions iPDP as a generalizable strategy for precision medicine.

Despite these therapeutic benefits, the current device has several limitations that require further optimization. First, the percutaneous optical fibers, while enabling precise and programmable light delivery, may introduce a potential infection risk and require a small skin incision for each activation. Although the device is implanted during the same surgery used to induce MI, the need for repeated percutaneous access could limit long‑term clinical acceptance. Future iterations could adopt fully implantable, wirelessly powered micro‑LEDs [23, 25, 26] to eliminate the percutaneous interface, enabling less invasive triggering and improving translational potential. Moreover, in the current prototype, although the device is biocompatible, it is non‑biodegradable. Future iterations could be designed as fully biodegradable to eliminate any concerns associated with permanent material residence. Biodegradable epicardial photonic patches, such as iCarP, have been developed for minimally invasive implantation onto beating hearts, demonstrating safety and efficacy in large‑area illumination without tissue puncture [27]. Additionally, biodegradable hydrogels with tunable degradation rates [53, 54, 55] could be integrated into our platform. Further, the optimal hydrolysis rate for post‑MI therapy has not been systematically determined; future studies comparing different release kinetics in animal models would help identify the ideal profile for myocardial repair. Based on these limitations, future efforts may focus on technical miniaturization (e.g., wireless light sources), the integration of biofeedback for autonomous control, and the development of combinatorial regimens using multi‐wavelength‐sensitive prodrugs to enable complex, stage‐specific therapies within a single device. Key steps toward clinical application include replacing the percutaneous fiber with a fully implantable, wirelessly powered micro‑LED and using biodegradable elastomers for the patch substrate, as well as the pivotal validation in large animal models (e.g., porcine MI model) to evaluate long‑term safety and efficacy. The platform's adaptability to different drugs and release kinetics positions it as a versatile tool for on‑demand therapy in various chronic conditions.

## 4 Conclusion

This work developed an implantable, actively programmable prodrug depot (iPDP) that integrates a phototriggered, sustained‐release prodrug hydrogel with a percutaneous light guide, addressing the critical challenge of controlling drug release kinetics in deep tissues after implantation. The core innovation is that transient optical signals serve as precise inputs to initiate and define sustained drug release programs. Upon brief illumination, rapid photolysis of the prodrug triggers a cascade that autonomously yields prolonged drug release, with the total dose per event precisely dictated by the light parameters. This process, once integrated into the hydrogel network, enables programmable control over both the dose per trigger and the schedule of multiple release cycles. Clinically, this enables percutaneous, surgery‐free programming of both release timing and dosage. For post‐MI therapy, the iPDP delivers spatiotemporally controlled release. In rat models, an actively programmed multi‐dose regimen, which fully spans the pathological continuum from inflammation to remodeling, demonstrates superior efficacy in improving cardiac function and structure and suppressing maladaptive inflammation, thereby effectively addressing the dynamic pathological progression. This platform principle holds considerable potential for adaptation as an on‐demand treatment modality for a range of other deep‐seated conditions through the incorporation of suitable therapeutic agents.

## 5 Experimental Section

### 5.1 Synthesis of GTel‐Hydrogels

GTel‐hydrogels were synthesized via redox‐initiated free radical copolymerization of GTel‐mono with three acrylate monomers: DMA, 2‐hydroxyethyl methacrylate (HEMA), and oligo(ethylene glycol) monomethacrylate (OEGMA), at different feed ratios as specified in Table S1. A typical procedure for preparing the prodrug hydrogel PDMA‐GTel10 is described as follows: 180 mg of the main monomer DMA, 20 mg of the functional comonomer GTel‐mono, and 1.0 mg of the crosslinker ethylene glycol dimethacrylate were mixed thoroughly. Subsequently, 26 µL of an ammonium persulfate solution (24 mg mL^−1^) and 5 µL of *N*, *N*, *N*′, *N*′‐tetramethylethylenediamine were added sequentially to the mixture, which was then immediately vortexed to ensure homogeneity. An aliquot of the precursor solution was sandwiched between two clean glass slides using a 165 µm‐thick coverslip as a spacer to define the hydrogel thickness (Scheme S2). The polymerization was allowed to proceed at 25°C for 24 h. After polymerization, the resulting hydrogel was thoroughly washed to remove unreacted monomers and impurities. This was achieved by sequential immersion in ethanol and dichloromethane, each for three cycles, followed by solvent exchange with ethanol and ultrapure water, also for three cycles each. The obtained hydrogel was denoted as PDMA‐GTel10, where the number following “GTel” indicates the mass percentage of GTel‐mono relative to the total mass of monomers.

Following an analogous procedure, a series of hydrogels including PDMA‐GTel20, PDMA‐GTel30, POEGMA‐GTel10, PHEMA‐GTel10, and PDH‐GTel10 (where “DH” represents an equimass mixture of DMA and HEMA) were similarly prepared for comparative studies.

### 5.2 In Vitro Drug Release and Drug Loading Capacity Determination of GTel‐Hydrogels

#### 5.2.1 General Procedures

Sample preparation: The GTel‐hydrogels were cut into small slices with a uniform thickness of approximately 400 µm and a comparable size of approximately 2 cm^2^. The samples were dried and accurately weighed to obtain a dry mass of approximately 5 mg.

Drug release incubation: The weighed dry gels were equilibrated in ultrapure water and then transferred individually to vials containing 2 mL of release medium (ultrapure water containing 0.5% (w/v) Tween‐80). These vials were incubated in a thermostatic shaker at 37°C and 120 rpm in the dark. At specific intervals, 1 mL of the release medium supernatant was withdrawn from each vial for HPLC analysis and replaced with an equal volume of fresh release medium. All procedures were performed under light‐protected conditions unless otherwise specified.

HPLC analysis: Drug quantification was performed by HPLC using a Thermo Scientific Hypersil GOLD C18 column (250 × 4.6 mm, 5 µm). The mobile phase was acetonitrile/0.05 M potassium phosphate monobasic aqueous solution (50:50, v/v) with a flow rate of 1 mL min^−1^. Detection was carried out at 295 nm with an injection volume of 20 µL.

#### 5.2.2 In Vitro Phototriggered Drug Release Profile

To investigate phototriggered drug release, a light source (LED or laser) at 520 nm was employed, with power density and irradiation duration set according to the specific experimental design. For the single‐irradiation groups, samples were irradiated once before immersion in the release medium. For the multiple‐irradiation groups, samples were briefly taken out at specific time points for additional irradiation after the initial pre‐immersion exposure. After irradiation, the sample was returned to the shaker for continued incubation in the dark. The cumulative amount of drug released was quantified by HPLC.

#### 5.2.3 Drug Loading Capacity Determination

The actual drug loading content of the GTel‐hydrogels was determined by measuring the cumulative drug release over a 30‐day period following irradiation for 1 h using a 520 nm LED light source (50 mW cm^−2^). The cumulative amount of drug released was quantified by HPLC. The drug loading content (µg mg^−1^) was calculated by dividing the total released drug amount by the dry mass of the hydrogel.

### 5.3 In Vitro Dark Stability Test of GTel‐Hydrogels

The dark stability of the GTel‐hydrogels in vitro, defined as the non‐specific release of the drug in the absence of light, was evaluated by HPLC. The procedures for sample preparation, incubation, and HPLC analysis were identical to those in Section 5.2, but no light irradiation was applied.

### 5.4 Fabrication and Characterization of the Implantable Phototriggered Prodrug Depot Patch

#### 5.4.1 Fabrication of the Light‐Guiding Device

The light‐guiding device featured a TOF seamlessly integrated into a PDMS substrate. The TOF was fabricated and surface‐treated following a previously reported protocol with slight modifications [27]. Briefly, a Fujikura 80S+ fusion splicer was operated under the [MM‐MM/Taper splice] mode with an elongation length set to 400 µm to produce a tapered region of 280 µm in length and a tip diameter of approximately 1 µm. The TOF was subsequently surface‐activated via plasma cleaner‐induced hydroxylation, followed by a 12‐hour vapor‐phase fluorination in a vacuum desiccator with trichloro(1H,1H,2H,2H‐tridecafluoro‐*n*‐octyl)silane under a pressure below 0.09 MPa, thereby depositing a uniform low‐surface‐energy coating on the fiber surface.

For PDMS disc encapsulation and device integration, a custom‐made polytetrafluoroethylene mold was fabricated, featuring a central circular pit (8 mm diameter, 400 µm depth) with a peripheral notch (200 µm × 200 µm). The processed TOF was carefully embedded into this notch, with its tapered tip suspended within the central area of the pit. Following this, a pre‐mixed PDMS prepolymer with a base‐to‐curing agent ratio of 10:1 by mass was poured into the mold until slightly overfilled. The assembly was degassed in a vacuum desiccator for 30 min to remove air bubbles. After degassing, a polyester film was applied, and a slight weight was placed to ensure surface flatness during thermal curing at 80°C for 30 min. After demolding, the optical fiber was gently pulled outward by approximately 1 mm to create a precisely controlled air gap between the tapered tip and the PDMS matrix. This design enables wide‐angle light scattering (approximately 120°).

#### 5.4.2 Fabrication and Characterization of the Implantable Phototriggered Prodrug Depot Patch

To ensure precise alignment with the light field, the hydrogel geometry was first optimized during the fabrication of the iPDP. The photobleached area was measured on an 8 mm diameter circular hydrogel that matched the PDMS disc, revealing an approximately 120° fan‐shaped central region positioned 1 mm from the upper edge and 3 mm from the lower edge at the bottom (Figure S28a‐iii). Accordingly, a PDMA‐GTel hydrogel was precision‐cut into a matching 120° fan shape with a radius of 4 mm. This fan‐shaped segment was then preliminarily bonded to the center of the PDMS disc using 10 µL of medical cyanoacrylate, forming the final iPDP device. All device characterizations and in vivo studies reported in the main text were performed using this PDMA‐GTel20 formulation. For in vivo implantation, the device was then surgically sutured onto the myocardial surface via the PDMS disc edge. The key geometric dimensions (radius, thickness, and central angle) of the fabricated iPDP devices were measured using a digital thickness gauge and ImageJ software for statistical analysis.

#### 5.4.3 Connection of the iPDP Device to the Laser Source

To enable in vivo light irradiation, the optical fiber tail of the iPDP device was connected to an external laser source. Specifically, the distal end of the fiber was first stripped and cleaved to produce a clean, flat end‐face. An FC‐type fiber optic connector with a 130 µm ferrule was then assembled onto the prepared fiber tip. Finally, the device fiber was connected to the SMA‐terminated laser source via an FC‐to‐SMA adapter, thereby establishing a complete and robust optical path. The output power of the 520 nm laser used in this study was continuously tunable by adjusting its drive current. The irradiance values specified for iPDP experiments (e.g., 10 or 35 mW cm^−2^) refer to the measured values at the emission surface of the light‐guiding device (prior to hydrogel attachment) under corresponding current settings (e.g., 650 or 1440 mA), as quantified using the irradiance meter (FZ‐A). This value represents the incident irradiance delivered to the prodrug hydrogel in the fully integrated iPDP.

### 5.5 In Vitro Device‐Based Drug Release and Stability Testing of the iPDP Device

The in vitro drug release profile of the iPDP device was evaluated by HPLC, with incubation conditions and HPLC analysis identical to those described in Section 5.2. For the single‐trigger groups, each iPDP device was connected to the laser for activation over a specified duration. Immediately afterward, all fiber optic connections were severed and removed, and the device was immersed in the release medium for dark incubation. For the multi‐trigger groups, to facilitate repeated handling, the hydrogel of each iPDP device was detached and transferred to fresh release medium for incubation after each laser activation, and it was taken out and re‐attached for each subsequent trigger. The actual drug loading content of the iPDP device was determined by measuring the cumulative drug release over a 30‐day period following irradiation for 1 h using a 520 nm LED light source (50 mW cm^−2^).

The dark stability of the iPDP device in vitro was assessed via the drug release test without laser input or any other form of light exposure.

For more physiologically relevant conditions, release tests were also performed with the hydrogel remaining attached to the PDMS disc throughout the entire incubation period. The release medium was DMEM supplemented with 10% fetal bovine serum (FBS) instead of 0.5% Tween. Before HPLC analysis, four volumes of acetonitrile were added to one volume of sample to precipitate serum proteins, followed by centrifugation at 16 000 rpm for 2 min. The supernatant was then collected for drug quantification. For triple‑trigger experiments, the assembled iPDP device was incubated in a container sealed with parafilm through which the optical fiber was passed.

### 5.6 In Vivo Illumination Procedure of the iPDP Device

The iPDP device was surgically implanted in male Sprague–Dawley rats (6 weeks old, 200 ± 20 g) under anesthesia induced by an intraperitoneal injection of 1% sodium pentobarbital. Following endotracheal intubation and mechanical ventilator support, a left thoracotomy and pericardiotomy were performed to expose the heart. The iPDP device was placed on the epicardial surface of the left ventricular (LV) infarct area, oriented with the optical fiber toward the abdomen, and fixed to the myocardium with two interrupted 6‐0 polypropylene sutures placed at opposite edges of the PDMS disc.

Initial illumination was performed after device fixation and layered thoracic closure, during which the optical fiber tail was exteriorized. Following extubation upon the resumption of stable spontaneous respiration, the exposed fiber end was cleaned, stripped, and cleaved to obtain a clean end‐face. It was subsequently connected to an external 520 nm laser source via an FC‐to‐SMA adapter for a specified irradiation period to trigger drug release. Upon completion, the fiber was disconnected from the laser source, gently coiled into a subcutaneous loop in the abdominal region, and its terminal end was secured in the lower abdomen.

For subsequent illuminations, rats were re‐anesthetized with sodium pentobarbital without the need for re‐intubation or thoracotomy. A small abdominal incision was made, and the buried fiber tail was carefully exteriorized. The fiber end was then re‐cleaned and connected to the laser for illumination. Following the procedure, the fiber was re‐embedded subcutaneously, and the incision was closed with sutures.

### 5.7 Rat Myocardial Infarction Treatment

An MI model was established in rats using permanent ligation of the LAD coronary artery [56]. Briefly, rats were anesthetized with an intraperitoneal injection of 1% sodium pentobarbital, endotracheally intubated, and mechanically ventilated. Under aseptic conditions, the heart was exposed through a left thoracotomy and pericardiotomy, followed by permanent ligation of the LAD coronary artery with a 6‐0 silk suture to induce acute MI. A total of 20 male Sprague–Dawley rats (6 weeks old, 200 ± 20 g) were randomly assigned to 5 groups. (i) The Sham group underwent thoracotomy without LAD ligation. (ii) The MI group received LAD ligation without any subsequent treatment. (iii) The iPDP with no release group received iPDP implantation without illumination. (iv) The iPDP with a single release group received iPDP implantation followed by immediate single illumination (520 nm laser, 35 mW cm^−2^, 60 min), yielding a total released telmisartan dose of approximately 10 µg. (v) The iPDP with triple‐programmed release group received iPDP implantation with fractionated illumination on postoperative days 0, 2, and 4 using the following parameters: day 0: 10 mW cm^−2^ for 1 min; day 2: 35 mW cm^−2^ for 5 min; day 4: 35 mW cm^−2^ for 20 min. The total released telmisartan dose was approximately 10 µg.

Ten minutes after ligation, for rats in groups iii, iv, and v, the iPDP device was implanted onto the epicardial surface of the infarcted area and sutured in place as described previously. Following device implantation, the thoracic incision was closed in layers for all groups. In the iPDP with no release group, the optical fiber was directly embedded subcutaneously in the abdomen. In both the iPDP with a single release and iPDP with triple‐programmed release groups, the first illumination was conducted according to the in vivo illumination procedure (Section 5.6) after chest closure and stabilization of spontaneous respiration. The iPDP with triple‐programmed release group subsequently received additional illuminations on postoperative days 2 and 4, following the same protocol.

On postoperative day 7, cardiac function was assessed by echocardiography using a Vevo 2100 system (Visual Sonics, Canada). Two‐dimensional and M‐mode images were acquired from the parasternal long‐axis and short‐axis views at the papillary muscle level. From the M‐mode tracings, heart rate and LV dimensions were measured, including LV end‐diastolic and end‐systolic internal diameters as well as diastolic and systolic wall thickness. The LV ejection fraction and fractional shortening were calculated from these primary measurements.

After echocardiography, the rats were euthanized, and their heart tissues were harvested. The hearts were rinsed in phosphate‐buffered saline (1×, pH 7.4), arrested in 10% potassium chloride solution, and then carefully dissected. The larger part of each heart, encompassing approximately half of the infarcted area along with the surrounding and remote myocardium, was fixed in 4% paraformaldehyde for subsequent paraffin embedding and sectioning. The remaining portion of tissue from the infarcted area was immediately frozen in liquid nitrogen and stored at −80°C for later RNA sequencing. Consecutive histological analyses were performed to evaluate therapeutic outcomes. Masson's trichrome staining was used to determine the infarct size, expressed as the percentage of fibrotic area (blue staining) relative to the total cross‐sectional area of the left ventricle. Immunohistochemical staining for TNF‐α and immunofluorescence double staining for IL‐6/DAPI were employed to assess inflammatory factor expression. Furthermore, macrophage polarization was evaluated via immunofluorescence triple staining for CD86, CD163, and DAPI, with CD86‐positive (M1) and CD163‐positive (M2) macrophages quantified for statistical analysis. Cardiomyocyte apoptosis was detected using the TUNEL assay. Additionally, immunofluorescence triple staining for Cx43, cTnT, and DAPI was performed to assess cardiomyocyte integrity and Cx43 localization. All histological images were analyzed using ImageJ software.

### 5.8 RNA Sequencing and Analysis

Total RNA from all frozen tissue samples was extracted, quality‐controlled, and sequenced on an Illumina platform by Novogene Co., Ltd. (Beijing, China), which also performed strand‐specific library preparation. After quality control, high‐quality reads were aligned to the rat reference genome using Hisat2, and gene expression was quantified with featureCounts. Differential expression analysis was performed using DESeq2, defining genes with an adjusted *P*‐value < 0.05 and |log_2_(fold change)| > 1 as significant. Subsequent analyses included principal component analysis, hierarchical clustering of DEGs, volcano plot visualization, GO enrichment analysis, and KEGG pathway enrichment analysis. GSEA was further conducted to evaluate the enrichment of predefined KEGG pathways in the ranked gene list, with significance assessed by the normalized enrichment score (NES) and false discovery rate (FDR).

### 5.9 Cell Proliferation Inhibition Assay of the iPDP Device

The inhibitory effect of the drug released from the iPDP device on the proliferation of L929 fibroblasts was evaluated in vitro using a Transwell co‐culture system (0.4 µm pore size). The experimental groups were designed as follows. (i) iPDP devices loaded with approximately 5.5, 11, 22, and 44 µg of drug, achieved by incorporating 0.25, 0.5, 1, and 2 pieces of 6‐mm‐diameter circular PDMA‐GTel10 hydrogel discs (each containing 21.8 ± 2.8 µg of telmisartan), respectively, all of which were pre‐irradiated for 20 min using a 520 nm LED at a power density of 50 mW cm^−2^. (ii) iPDP devices containing a single piece of the same hydrogel but without any irradiation. (iii) iPDP devices incorporating one piece of a drug‐free blank hydrogel, also without irradiation. (iv) Cells without any device treatment, which served as the negative control and were assigned 100% viability. All iPDP devices were sterilized by immersion in 75% ethanol prior to the assay.

Additionally, a dose‐response assay of free telmisartan was conducted for direct comparison. L929 cells were seeded in a 24‐well plate at a density of 1 × 10^4^ cells per well and cultured for 24 h for attachment. The medium was then replaced with fresh complete medium containing telmisartan at gradient concentrations (0, 5.6, 11.3, 22.5, and 45 µg mL^−1^) and incubated for another 72 h.

For both the device‐based and free‐drug assays, cell viability was assessed using the cell counting kit‐8 (CCK‐8) method after the incubation period. Briefly, 100 µL of medium from each well was transferred to a 96‐well plate, mixed with 10 µL of CCK‐8 solution, and incubated for 2–4 h. Absorbance at 450 nm was measured using a microplate reader. The cell proliferation inhibition rate was calculated using the equation:

(1) $$\mathrm{Inhibition}\;\mathrm{rate}\;\left(\%\right)=\left(1\;-\;\frac{Abs_{\exp}}{Abs_{\mathrm{ctrl}}}\right)\;\times \;100\%$$

where *Abs*_exp_ and *Abs*_ctrl_ are the absorbance values of the experimental and control groups, respectively.

### 5.10 Cell Migration Inhibition Assay of the iPDP Device

The effects of the iPDP device on the migration of L929 fibroblasts were evaluated using a Transwell assay. The experimental groups were designed to assess the inhibitory effects under different conditions against a background of 100 nm angiotensin II (Ang II). (i) The control group was cultured in normal medium without Ang II or other additives. (ii) The Ang II‐Migration group was treated with Ang II‐containing medium to establish the baseline migratory response. (iii) The Tel‐Inhibition group was exposed to medium supplemented with both Ang II and 20 µg mL^−1^ telmisartan. (iv) The iPDP‐NoLight group received medium containing Ang II along with the extract from non‐irradiated devices. (v) The iPDP‐Light group was treated with medium containing Ang II and the extract from the pre‐irradiated devices. For the device groups (iv and v), the hydrogel quantity was standardized to yield a theoretical maximum telmisartan concentration of 20 µg mL^−1^, ensuring comparability with group (iii).

To prepare the required extracts, non‐irradiated and pre‐irradiated iPDP devices were prepared and incubated separately in complete medium with 10% fetal bovine serum (FBS) under aseptic conditions at 37°C for 3 days in the dark. The pre‐irradiated devices were treated with a 520 nm LED at 50 mW cm^−2^ for 20 min prior to incubation. The resulting supernatant, serving as the extract for the lower chamber, was collected and stored at 4°C for later use. In parallel, devices from the same non‐irradiated and pre‐irradiated groups were separately immersed in medium with 0.5% FBS to generate the corresponding upper chamber treatment media.

For the migration assay, 24‐well plates and Transwell inserts with an 8 µm pore size were used. Briefly, L929 cells were serum‐starved for 12 h and then prepared as a cell suspension. Cells for each group were resuspended in their respective treatment media (all containing 0.5% FBS). A 100 µL aliquot of each cell suspension containing 2 × 10^4^ cells was seeded into the upper chamber of each insert. The lower chamber of each well was filled with 600 µL of the matching complete medium containing 10% FBS to establish a chemotactic gradient. After incubation for 4 h, cells on the membrane of the Transwell inserts were fixed with 4% paraformaldehyde and stained with 0.1% crystal violet. Subsequently, non‐migrated cells on the upper surface of the membrane were removed with a cotton swab. The membranes were imaged under a microscope, and the number of migrated cells in multiple randomly selected fields was counted.

### 5.11 Statistical Analysis

All data are presented as mean ± standard deviation (SD). All data points were included in the final analysis; no outliers were excluded. Normality of the data was assessed using the Shapiro–Wilk test (as recommended for small sample sizes). For comparisons between two groups, an unpaired two‑tailed Student's *t*‑test was used. For comparisons involving more than two groups, one‑way analysis of variance (ANOVA) followed by Tukey's post‑hoc test was applied. A *P*‐value of less than 0.05 was considered statistically significant, denoted in figures as follows: ns (not significant), *p < 0.05, **p < 0.01, ***p < 0.001. All statistical tests were performed using GraphPad Prism (version 10.0).

## Funding

National Key Research and Development Program of China (Grant No. 2025YFE0125200), National Natural Science Foundation of China (Grant No. 52425305 and 82470524), Lingyan Program of Zhejiang Province (Grant No. 2024C03074), Fundamental Research Funds for the Central Universities (Grant No. 226‐2024‐00147).

## Ethics Statement

The animal experiments have been reviewed and approved by the institutional animal care and use committee (IACUC) of Zhejiang Center of Laboratory Animals (approval No. ZJCLA‐IACUC‐20010782).

## Conflicts of Interest

The authors declare no conflicts of interest.

## Supporting information




**Supporting File**: advs76161‐sup‐0001‐SuppMat.docx.

## Data Availability

The data that support the findings of this study are available from the corresponding author upon reasonable request.
