# Supplementary material for: An Implantable Phototriggered Prodrug Depot Patch Enables Actively Programmable Drug Release for Post‐Myocardial Infarction Therapy
Source: Adv Sci (Weinh). 2026 Jun 26:e76161. Online ahead of print. doi: 10.1002/advs.76161 (PMC13336649; doi:10.1002/advs.76161)
Supplement: Supplementary file 1 — Supporting File: advs76161‐sup‐0001‐SuppMat.docx. [file ADVS-9999-e76161-s001.docx]

**Supporting Information**

**An Implantable Phototriggered Prodrug Depot Patch Enables** **Actively Programmable Drug Release for Post-Myocardial Infarction Therapy**

Haipeng Lu, Kaicheng Deng, Zhang Zhang, Qirui Wang, Weijing Gao, Liyin Shen, Lei Zhang, Wenting Hu^*^, Yang Zhu, Zhengwei Mao^*^, Tanchen Ren^*^

**1. Reagents and Materials**

The following reagents and solvents were employed in this study: 2-nitrobenzene-1,3-diacetic acid (Energy Chemical, 98%), borane tetrahydrofuran complex solution (Aladdin, 1 M in tetrahydrofuran), nickel(Ⅱ) acetate tetrahydrate (Macklin, 99%), sodium borohydride (Sinopharm Chemical Reagent, 98%), *tert*-butyldimethylsilyl chloride (Macklin, 97%), 1,8-diazabicyclo[5.4.0]undec-7-ene (Macklin, 99%), oligo(ethylene glycol) monomethacrylate (OEGMA, Sigma-Aldrich, *M*_n_ = 500), pyridine (Sinopharm Chemical Reagent, 99.5%), 4-dimethylaminopyridine (DMAP, Aladdin, 99%), succinic anhydride (Macklin, 99%), 2,4-dimethylpyrrole (Aladdin, 97%), acetoxyacetyl chloride (Energy Chemical, 97%), diisopropylethylamine (Energy Chemical, 99.5%), boron trifluoride diethyl etherate (Energy Chemical, 98%), methylmagnesium bromide (Energy Chemical, 3 M in ethyl ether), triphosgene (Macklin, 99%), tetrabutylammonium fluoride (Aladdin, 1 M in tetrahydrofuran), telmisartan (Aladdin, 98%), 1-ethyl-3-(3-dimethylaminopropyl)carbodiimide hydrochloride (Macklin, 98%), N,N-dimethylacrylamide (DMA, Aladdin, 99%), 2-hydroxyethyl methacrylate (HEMA, Aladdin, 96%), ethylene glycol dimethacrylate (Macklin, 98%), ammonium persulfate (Sinopharm Chemical Reagent, 98%), *N*,*N*,*N*′,*N*′-tetramethylethylenediamine (Macklin, 99%), trichloro(1H,1H,2H,2H-tridecafluoro-*n*-octyl)silane (Aladdin, 97%), crystal violet (Aladdin, 90%), tween-80 (MedChemExpress). Common inorganic salts and solvents, including sodium chloride (NaCl), anhydrous sodium sulfate (Na_2_SO_4_), sodium hydrogen sulfate, ammonium chloride, potassium phosphate monobasic, potassium chloride, tetrahydrofuran, methanol, ethyl acetate (EA), petroleum ether (PE), acetonitrile (ACN), dichloromethane (DCM), and ethanol, were procured from commercial sources such as Sinopharm Chemical Reagent. All reagents and solvents were used as received without further purification. Clinically used side glow optical fiber and matt flat-end optical fiber were purchased from Nanjing Chunhui Technology Industry (China). Dulbecco’s modified Eagle’s medium, fetal bovine serum (FBS) and penicillin/streptomycin were obtained from Gibco (USA). Cell counting kit-8 (CCK-8) and angiotensin Ⅱ (Ang Ⅱ) were purchased from TargetMol (USA). Antibodies against Interleukin-6 (IL-6), Cluster of Differentiation 86 (CD86), Cluster of Differentiation 163 (CD163), and corresponding secondary antibodies were sourced from Abcam (UK). 4′,6-diamidino-2-phenylindole (DAPI) was acquired from Sigma (USA). Water used in all experiments was purified using a Millipore Milli-Q system.

^1^H nuclear magnetic resonance spectrometry (^1^H NMR, Advance Ⅲ 400, Bruker, Germany), high resolution mass spectrometry (HRMS, G6545 QTOF ESI MS, Agilent, USA), high-performance liquid chromatography (HPLC, 1260 Infinity Ⅲ, Agilent, USA), liquid chromatography/mass spectrometry (LC/MS, 6475 Triple Quadrupole, Agilent, USA), Fourier transform infrared spectrometry (FT-IR, TENSOR Ⅱ, Bruker , Germany), microplate reader (Varioskan LUX Multimode, Thermo Fisher Scientific, USA), X-ray photoelectron spectroscopy (XPS, K-Alpha, Thermo Fisher Scientific, USA), and an electro-mechanical universal testing machine (AG-IC, Shimadzu, Japan) were employed to characterize the chemical structures and properties of the synthesized chemicals and hydrogels. Irradiations were carried out employing either a LED lamp (*λ* = 520–530 nm, JG, Xuzhou Ai Jia Electronic Technology, China) or laser (*λ* = 520 ± 5 nm, LR-MFJ-520/600 mW, Changchun Laser Technology, China). The light irradiance was determined by irradiance meter (400–1000 nm, FZ-A, Beijing Shida Photoelectric Technology, China).

**2. Experimental Section**

***2.1. Synthesis and characterization of the light-triggered prodrug (GTel)***

***Synthesis of (2-nitrobenzene-1,3-diyl)dimethanol (1)***

The 2-nitrobenzene-1,3-diacetic acid (4.07 g, 19.3 mmol, 1.0 eq.) was dissolved in 80.0 mL anhydrous tetrahydrofuran and stirred in an ice bath for 15 minutes. Borane tetrahydrofuran complex solution (100.0 mL, 100.0 mmol, 5.2 eq.) was added to the solution within 1 hour. Then, the mixture was stirred at room temperature for 48 hours. Methanol (40.0 mL) was added slowly via a syringe and the mixture was concentrated in vacuo. The residue was extracted with EA, washed with saturated NaCl solution and dried over anhydrous Na_2_SO_4_. The solution concentrated until only little EA was left and precipitated into PE. **(2-nitrobenzene-1,3-diyl)dimethanol (1)** was obtained (3.18 g, yield 90.1%) as white solid without extra purification. ^1^H NMR (500 MHz, DMSO-*d*6, 25 °C, TMS, ppm): *δ* = 7.62–7.50 (m, 3H), 5.48 (t, *J* = 5.6 Hz, 2H), 4.54 (d, *J* = 5.6 Hz, 4H). ^13^C NMR (125 MHz, DMSO-*d*6, 25 °C, TMS, ppm): *δ* = 147.39, 134.31, 130.73, 127.52, 59.23. HRMS(ESI): m/z calculated for C_8_H_8_NO_4_^⁻^ [M − H]^⁻^: 182.0459, found: 182.0458.

***Synthesis of 2,6-bis(hydroxymethyl)aniline (BHA, 2)***

The (2-nitrobenzene-1,3-diyl)dimethanol (**1**, 3.68 g, 20.1 mmol, 1.0 eq.) was mixed with nickel(Ⅱ) acetate tetrahydrate (1.04 g, 4.2 mmol, 0.21 eq.) in ACN/H_2_O (66.0 mL/7.0 mL) solution in a 500 mL beaker. Solid sodium borohydride (3.85 g, 101.7 mmol, 5.1 eq.) was slowly added into the mixture under vigorous stirring. After 20 minutes, the reaction was terminated by the addition of 70 mL water. The product was extracted with EA, washed with saturated NaCl solution, dried over anhydrous Na_2_SO_4_, and concentrated by a rotary evaporator. **2,6-bis(hydroxymethyl)aniline (2)** was obtained (3.04 g, yield 98.8%) as white solid without extra purification. ^1^H NMR (500 MHz, DMSO-*d*6, 25 °C, TMS, ppm): *δ* = 6.99 (d, *J* = 7.4 Hz, 2H), 6.52 (t, *J* = 7.4 Hz, 1H), 5.03 (t, *J* = 5.4 Hz, 2H), 4.81 (s, 2H), 4.42 (d, *J* = 5.3 Hz, 4H). HRMS(ESI): m/z calculated for C_8_H_12_NO_2_⁺ [M + H]⁺: 154.0863, found: 154.0863.

***Synthesis of 2,6-bis[(tert-butyldimethylsilyloxy)methyl]aniline (BHA-OTBDMS_2_, 3)***

The 2,6-bis(hydroxymethyl)aniline (**2**, 3.04 g, 19.8 mmol, 1.0 eq.) and *tert*-butyldimethylsilyl chloride (8.97 g, 59.5 mmol, 3.0 eq.) were dissolved in 70.0 mL ACN and stirred in an ice bath. Diazabicycloundecene (9.13 g, 60.0 mmol, 3.0 eq.) in 10.0 mL ACN was added dropwise to the solution within 30 minutes. Then, the mixture was stirred for 24 hours at room temperature. After evaporating the solvent, the residue was extracted with EA, washed with saturated NaCl solution, dried over anhydrous Na_2_SO_4_, and concentrated by a rotary evaporator. The resultant crude product was purified by column chromatography on silica gel (PE/EA, 20:1 v/v) to afford **2,6-bis[(*tert*-butyldimethylsilyloxy)methyl]aniline** (**3**, 7.28 g, yield 96.2%) as a colorless oil. ^1^H NMR (400 MHz, DMSO-*d*6, 25 °C, TMS, ppm): *δ* = 7.04 (d, *J* = 7.5 Hz, 2H), 6.56 (t, *J* = 7.5 Hz, 1H), 4.71 (s, 2H), 4.62 (s, 4H), 0.88 (s, 18H), 0.06 (s, 12H). ^13^C NMR (100 MHz, DMSO-*d*6, 25 °C, TMS, ppm): *δ* = 143.50, 126.34, 124.36, 115.67, 62.87, 25.76, 17.89, -5.33. HRMS(ESI): m/z calculated for C_20_H_40_NO_2_Si_2_⁺ [M + H]⁺: 382.2592, found: 382.2593.

***Synthesis of succinic acid-modified oligo(ethylene glycol) monomethacrylate (OEGMA-COOH, 4)***

**OEGMA** (10.01 g, 19.0 mmol, 1.0 eq.) was dissolved in pyridine (4.84 mL, 59.8 mmol, 3.1 eq.) and DCM (10.0 mL) in a round bottom flask equipped with magnetic stir bar. DMAP (495.0 mg, 4.1 mmol, 0.21 eq.) was added and the reaction mixture cooled to 0 °C. Succinic anhydride (3.01 g, 30.1 mmol, 1.6 eq.) was added as a powder and the reaction was stirred vigorously for 12 hours. Water (2.0 mL) was added and the reaction allowed to stir for 6 hours. Subsequently, the reaction mixture was extracted with DCM, washed with 10% (w/w) aqueous sodium hydrogen sulfate and then with saturated NaCl solution, each for three times, dried over anhydrous Na_2_SO_4_, and concentrated by a rotary evaporator. The product was collected as a colorless viscous oil (11.34 g, 95.2%). ^1^H NMR (500 MHz, CDCl_3_, 25 °C, TMS, ppm): *δ* = 6.13 (s, 1H), 5.58 (s, 1H), 4.28 (dt, *J* = 18.1, 4.6 Hz, 4H), 3.77–3.62 (m, 36H), 2.68–2.63 (m, 4H), 1.95 (s, 3H). HRMS(ESI): m/z calculated for C_28_H_49_O_15_^⁻^ [M − H]^⁻^: 625.3077, found: 625.3073.

***Synthesis of 8-acetoxymethyl-1,3,5,7-tetramethyl pyrromethene fluoroborate (BODIPY-OAc, 5)***

2,4-Dimethyl pyrrole (10.0 mL, 97.1 mmol, 2.0 eq.) was dissolved in dry DCM. Acetoxyacetyl chloride (6.3 mL, 58.6 mmol, 1.2 eq.) was added to the solution, and the reaction was left stirring at 40 °C under argon. After 2 hours, it was cooled to room temperature and diisopropylethylamine (33.8 mL, 194.0 mmol, 4.0 eq.) was added followed after 15 minutes by dropwise addition of boron trifluoride diethyl etherate (24.0 mL, 194.5 mmol, 4.0 eq.). During addition of boron trifluoride diethyl etherate the color changed from pale yellow to dark red. The reaction was stopped after 24 hours, the solvent was evaporated under reduced pressure, and the crude reaction mixture was loaded onto a silica gel flash column and eluted with PE/EA (1:1, v/v) to give **BODIPY-OAc (5)** as orange-green crystals (4.87 g, yield 33.0%). ^1^H NMR (400 MHz, CDCl_3_, 25 °C, TMS, ppm): *δ* = 6.08 (s, 2H), 5.29 (s, 2H), 2.53 (s, 6H), 2.36 (s, 6H), 2.13 (s, 3H). ^13^C NMR (100 MHz, CDCl_3_, 25 °C, TMS, ppm): *δ* = 170.56, 156.63, 141.48, 133.32, 132.67, 122.32, 57.87, 20.60, 15.63, 14.69. HRMS(ESI): m/z calculated for C_16_H_19_BF_2_N_2_NaO_2_⁺ [M + Na]⁺: 343.1400, found: 343.1402.

***Synthesis of 4,4′-dimethyl-8-hydroxymethyl-1,3,5,7-tetramethyl-4-bora-3a,4a-diaza-s-indacene (DM-BODIPY-OH, 6)***

The BODIPY-OAc (**5**, 4.87 g, 15.2 mmol, 1.0 eq.) was dissolved in 400.0 mL of dry DCM, and methyl magnesium bromide (3M in ethyl ether, 100.0 mL, 300.0 mmol, 19.7 eq.) was added dropwise. The reaction was stirred at room temperature and monitored by thin-layer chromatography (PE/EA, 2:1 v/v). After the reaction was complete in 6 hours, it was diluted adding 400 mL DCM. 300 mL of saturated ammonium chloride was added to quench the reaction, and the organic phase was collected using a separating funnel. The organic phase was washed with the saturated NaCl solution and dried over Na_2_SO_4_. The solution was concentrated on a rotary evaporator and purified using flash chromatography on silica gel (PE/EA, 4:1 v/v) to obtain **DM-BODIPY-OH** (**6**, 2.68 g, yield 65.1%) as an orange solid. ^1^H NMR (400 MHz, CDCl_3_, 25 °C, TMS, ppm): *δ* = 6.09 (s, 2H), 4.96 (s, 2H), 2.53 (s, 6H), 2.46 (s, 6H), 0.18 (s, 6H). HRMS(ESI): m/z calculated for C_16_H_24_BN_2_O⁺ [M + H]⁺: 271.1976, found: 271.1978.

***Synthesis of DM-BODIPY-BHA-OTBDMS_2_ (7)***

To a dried flask, the triphosgene (1.13 g, 3.8 mmol, 0.64 eq.) was dissolved in 20.0 mL anhydrous DCM under argon, and the reaction mixture cooled to 0 °C. BHA-OTBDMS_2_ (**3**, 4.12 g, 10.8 mmol, 1.8 eq.) and DMAP (2.64 g, 21.6 mmol, 3.6 eq.) were dissolved in 40.0 mL anhydrous DCM, respectively, and added dropwise to the reaction mixture simultaneously. Then the mixture was stirred for 2 hours at 25 °C. Followed, DMAP(1.32 g, 10.8 mmol, 1.8 eq.) and DM-BODIPY-OH (**6**, 1.62 g, 6.0 mmol, 1.0 eq.) were added, and the mixture was stirred for 24 hours. After evaporating the solvent, the crude product was purified using silica gel column chromatography, yielding a red solid (2.70 g, yield 66.5%). ^1^H NMR (500 MHz, CDCl_3_, 25 °C, TMS, ppm): *δ* = 7.56 (s, 1H), 7.36 (d, *J* = 7.7 Hz, 2H), 7.24 (t, *J* = 7.6 Hz, 1H), 6.10 (s, 2H), 5.41 (s, 2H), 4.71 (s, 4H), 2.49–2.43 (m, 12H), 0.87 (s, 18H), 0.20 (s, 6H), 0.05 (s, 12H). HRMS(ESI): m/z calculated for C_37_H_60_BN_3_NaO_4_Si_2_⁺ [M + Na]⁺: 700.4108, found: 700.4112.

***Synthesis of DM-BODIPY-BHA-OH_2_ (8)***

**DM-BODIPY-BHA-OTBDMS_2_ (7**, 806.5 mg, 1.2 mmol, 1.0 eq.) was dissolved in tetrahydrofuran (60.0 mL), and then tetrabutylammonium fluoride (2.40 mL, 2.4 mmol, 2.0 eq.) was added. The mixture was stirred for 3 hours at −20 °C. The reaction solution was diluted with EA (200 mL) and washed with saturated NaCl solution (200 mL). The organic phases were dried over Na_2_SO_4_ and concentrated under vacuum. The resultant crude product was purified by column chromatography on silica gel (PE/EA, 1:1 v/v) to furnish **DM-BODIPY-BHA-OH_2_** (**8**, 477.1 mg, yield 89.2%) as a red solid. ^1^H NMR (500 MHz, CDCl_3_, 25 °C, TMS, ppm): *δ* = 7.60 (s, 1H), 7.39 (d, *J* = 7.5 Hz, 2H), 7.31–7.26 (m, 1H), 6.12 (s, 2H), 5.46 (s, 2H), 4.63 (s, 4H), 2.50–2.44 (m, 12H), 0.21 (s, 6H). HRMS(ESI): m/z calculated for C_25_H_32_BN_3_NaO_4_⁺ [M + Na]⁺: 472.2378, found: 472.2379.

***Synthesis of DM-BODIPY-BHA-OH-Tel (GTel, 9)***

Telmisartan (116.5 mg, 226.4 μmol, 1.0 eq.), DM-BODIPY-BHA-OH_2_ (**8**, 100.2 mg, 223.0 μmol, 1.0 eq.), and DMAP (27.5 mg, 225.1 μmol, 1.0 eq.) were dissolved in 15.0 mL of DCM and cooled in an ice bath. Then, 1-ethyl-3-(3-dimethylaminopropyl)carbodiimide hydrochloride (90.8 mg, 473.7 μmol, 2.1 eq) was added to the reaction mixture, which was then stirred for 24 hours at 25 °C. The reaction solution was diluted with EA (100 mL) and washed with saturated NaCl solution (100 mL). The organic phases were dried over Na_2_SO_4_ and concentrated under vacuum. The resultant crude product was purified by column chromatography on silica gel (PE/EA, 1:3 v/v) to furnish **GTel** (**9**, 83.5 mg, yield 39.6%) as a red solid. ^1^H NMR (500 MHz, DMSO-*d*6, 25 °C, TMS, ppm): *δ* = 9.17 (s, 1H), 7.79–7.72 (m, 2H), 7.64 (d, *J* = 7.2 Hz, 1H), 7.61–7.53 (m, 2H), 7.50–7.34 (m, 4H), 7.28–7.19 (m, 4H), 7.17–7.09 (m, 3H), 6.89–6.84 (m, 1H), 6.19 (s, 2H), 5.59 (s, 2H), 5.25–5.17 (m, 3H), 5.05 (s, 2H), 4.46 (d, *J* = 5.5 Hz, 2H), 3.81 (s, 3H), 2.89 (t, *J* = 7.6 Hz, 2H), 2.63 (s, 3H), 2.41 (s, 6H), 2.31 (s, 6H), 1.85–1.75 (m, 2H), 0.98 (t, *J* = 7.4 Hz, 3H), 0.12 (s, 6H). ^13^C NMR (125 MHz, DMSO-*d*6, 25 °C, TMS, ppm): *δ* =170.80, 167.80, 156.60, 154.64, 153.08, 137.74, 137.12, 136.55, 135.23, 132.33, 131.15, 129.17, 127.91, 126.92, 123.81, 123.18, 122.49, 122.23, 119.16, 110.82, 109.63, 60.22, 59.37, 46.47, 32.17, 29.21, 21.23, 21.12, 16.92, 16.66, 15.70, 14.56, 14.29. HRMS(ESI): m/z calculated for C_58_H_61_BN_7_O_5_⁺ [M + H]⁺: 946.4822, found: 946.4823.

***2.2. Synthesis and characterization of DM-BODIPY-BHA-OEGMA-Tel (GTel-mono, 10)***

GTel (**9**, 1.55 g, 1.6 mmol, 1.0 eq.), OEGMA-COOH (**4**, 1.13 g, 1.8 mmol, 1.1 eq.), and DMAP (41.3 mg, 0.34 mmol, 0.21 eq.) were dissolved in 100.0 mL of DCM and cooled in an ice bath. Then, 1-ethyl-3-(3-dimethylaminopropyl)carbodiimide hydrochloride (692.9 mg, 3.3 mmol, 2.1 eq) was added to the reaction mixture, which was then stirred for 24 hours at 25 °C. The reaction solution was diluted with EA (100 mL) and washed with saturated NaCl solution (100 mL). The organic phases were dried over Na_2_SO_4_ and concentrated under vacuum. The residue was dried under vacuum to afford **GTel-mono (10)** as a red, wax-like semi-solid (2.34 g, yield 91.9%). ^1^H NMR (500 MHz, DMSO-*d*6, 25 °C, TMS, ppm): *δ* = 9.41 (s, 1H), 7.78–7.72 (m, 2H), 7.63 (d, *J* = 7.8 Hz, 1H), 7.61–7.53 (m, 2H), 7.49–7.42 (m, 2H), 7.37 (d, *J* = 7.3 Hz, 1H), 7.29–7.17 (m, 5H), 7.16–7.08 (m, 3H), 6.94–6.89 (m, 1H), 6.18 (s, 2H), 6.02 (d, *J* = 5.1 Hz, 1H), 5.68 (d, *J* = 19.1 Hz, 1H), 5.59 (s, 2H), 5.25 (s, 2H), 5.07–4.98 (m, 4H), 4.23–4.06 (m, 4H), 3.80 (s, 3H), 3.66–3.43 (m, 36H), 2.88 (t, *J* = 7.6 Hz, 2H), 2.63 (s, 3H), 2.57 (s, 4H), 2.40 (s, 6H), 2.31 (s, 6H), 1.88 (s, 3H), 1.84–1.75 (m, 2H), 0.98 (t, *J* = 7.3 Hz, 3H), 0.11 (s, 6H). ^13^C NMR (125 MHz, DMSO-*d*6, 25 °C, TMS, ppm): *δ* = 172.34, 172.04, 167.83, 166.99, 156.58, 154.72, 154.51, 153.09, 143.15, 142.99, 141.47, 140.19, 137.78, 137.11, 136.57, 136.29, 135.22, 134.32, 134.21, 133.76, 133.70, 132.09, 131.14, 130.82, 130.04, 129.16, 128.92, 128.72, 127.92, 127.34, 126.89, 126.27, 123.82, 123.69, 123.18, 122.47, 122.22, 119.16, 110.80, 109.63, 72.82, 70.32, 70.25, 70.19, 68.72, 68.68, 64.20, 63.94, 63.16, 62.55, 60.68, 58.96, 55.38, 32.15, 29.19, 28.98, 28.94, 21.11, 18.44, 16.92, 16.65, 15.71, 14.28. HRMS(ESI): m/z calculated for C_86_H_108_BN_7_NaO_19_⁺ [M + Na]⁺: 1576.7685, found: 1576.7694.

***2.3. Cell culture***

H9c2 rat cardiomyocytes and L929 fibroblasts were obtained from the Cell Bank of Typical Culture Collection of Chinese Academy of Sciences (Shanghai, China). They were cultured with Dulbecco's Modified Eagle Medium containing 10% FBS, 100 μg mL^−1^ streptomycin, and 100 U mL^−1^ penicillin at 37 °C in a 5% CO_2_ incubator.

***2.4. Cytotoxicity assay of the iPDP device***

The cytotoxicity of the iPDP device and its released drug molecules was evaluated in vitro toward H9c2 rat cardiomyocytes using a Transwell co-culture system. This setup allows small molecular factors released from the devices to diffuse through the permeable membrane to interact with the cells in the lower chamber, while physically separating the devices themselves from direct contact with the cells.

The experimental groups and the co-culture procedure were identical to those described for the Cell proliferation inhibition assay (**Section 5.9**).

Briefly, H9c2 rat cardiomyocytes were seeded in a 24-well plate at a density of 1 × 10^4^ cells per well and cultured for 24 hours for attachment. Then the culture medium was replaced with fresh complete medium and the sterilized devices from various groups were placed into the corresponding Transwell inserts (Corning, 0.4 μm pore size). The inserts containing devices from the designated irradiation groups were immediately photo-irradiated. Following irradiation, all inserts were transferred to the corresponding wells of the 24-well plate, and 200 μL of fresh complete medium was then added to each insert. The plate was incubated for an additional 72 hours. After this co-culture phase, the Transwell inserts were carefully removed. To perform the CCK-8 assay, 100 μL of medium from each lower chamber was transferred to a 96-well plate, mixed with 10 μL of CCK-8 solution, and incubated for 2–4 hours. Absorbance at 450 nm was measured using a microplate reader. Relative cell viability was calculated as a percentage, normalized to the control group (set at 100%).

***2.5. Systemic toxicity assessment***

A total of 12 male Sprague-Dawley rats (6 weeks old, 200 ± 20 g) were randomly assigned to 3 groups. (ⅰ) The control group was established as a non-surgical healthy baseline. (ⅱ) The sham group was established as a surgical control, undergoing the complete operative procedure including thoracotomy, pericardiotomy, chest closure, and a mock abdominal incision without device implantation. (ⅲ) The iPDP group was established as the treatment group, receiving the iPDP implant followed by three illumination sessions on days 0, 2, and 4 (520 nm laser; day 0: 10  mW cm^−2^ for 1 min; day 2: 35  mW cm^−2^ for 5 min; day 4: 35  mW cm^−2^ for 20 min). Biospecimens were collected upon euthanasia on day 7 post-surgery for safety assessment. For hematological and biochemical analyses, whole blood was collected. A portion of each sample was used for complete blood count analysis, while the remaining blood was centrifuged to obtain serum for the assessment of key hepatic and renal function markers, including aspartate aminotransferase (AST), alanine aminotransferase (ALT), albumin (ALB), globulin (GLB), urea (UREA), and creatinine (CRE). For the histological assay, the main organs (including the heart, liver, spleen, lung, and kidney) were harvested, rinsed with phosphate-buffered saline, and then fixed in 4% paraformaldehyde for 24 hours. The specimens were dehydrated in graded ethanol, embedded in paraffin, and cut into 5-μm thick sections. The sections were deparaffinized, rehydrated, and stained with hematoxylin and eosin (H&E) for microscopic observation. Serum glutathione (GSH) levels were measured on day 7 using a commercial colorimetric assay kit (Suzhou Grace Biotechnology, China) according to the manufacturer’s instructions.

**3. Supporting Figures**

**Scheme S1.** Synthetic route of light-triggered prodrug (GTel, **9**) and light-triggered prodrug-containing monomer (GTel-mono, **10**). BH_3_·THF: borane tetrahydrofuran complex solution, NaBH_4_: sodium borohydride, NiAc_2_·4H_2_O: nickel(Ⅱ) acetate tetrahydrate, TBDMSCl: *tert*-butyldimethylsilyl chloride, DBU: diazabicycloundecene, DIPEA: diisopropylethylamine, BF_3_·OEt_3_: boron trifluoride diethyl etherate, TBAF: tetrabutylammonium fluoride, EDC·HCl: 1-ethyl-3-(3-dimethylaminopropyl)carbodiimide hydrochloride.


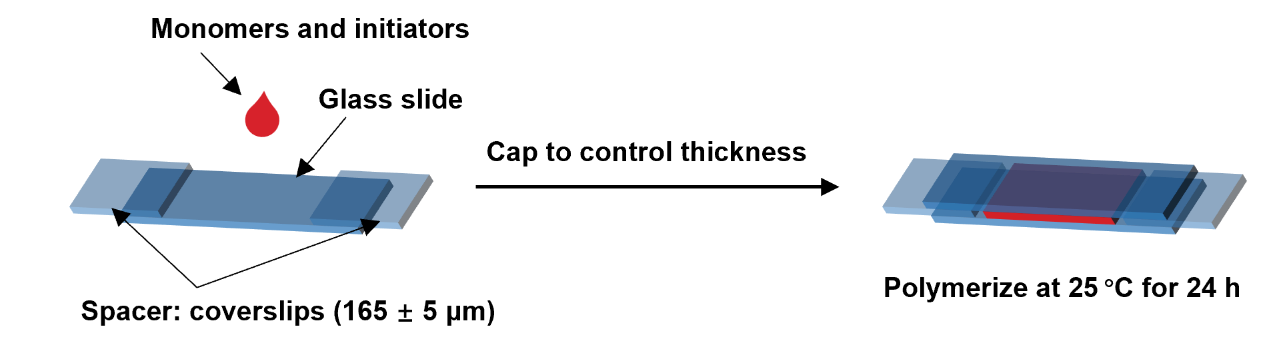


**Scheme S2.** Schematic illustration of the mold-casting process for fabricating GTel-hydrogel thin films. The precursor solution containing monomers and initiators is confined between two glass slides using 165 μm-thick coverslips as spacers to define the uniform thickness of the hydrogel. Polymerization proceeds at 25 °C for 24 hours to yield the freestanding prodrug hydrogel.


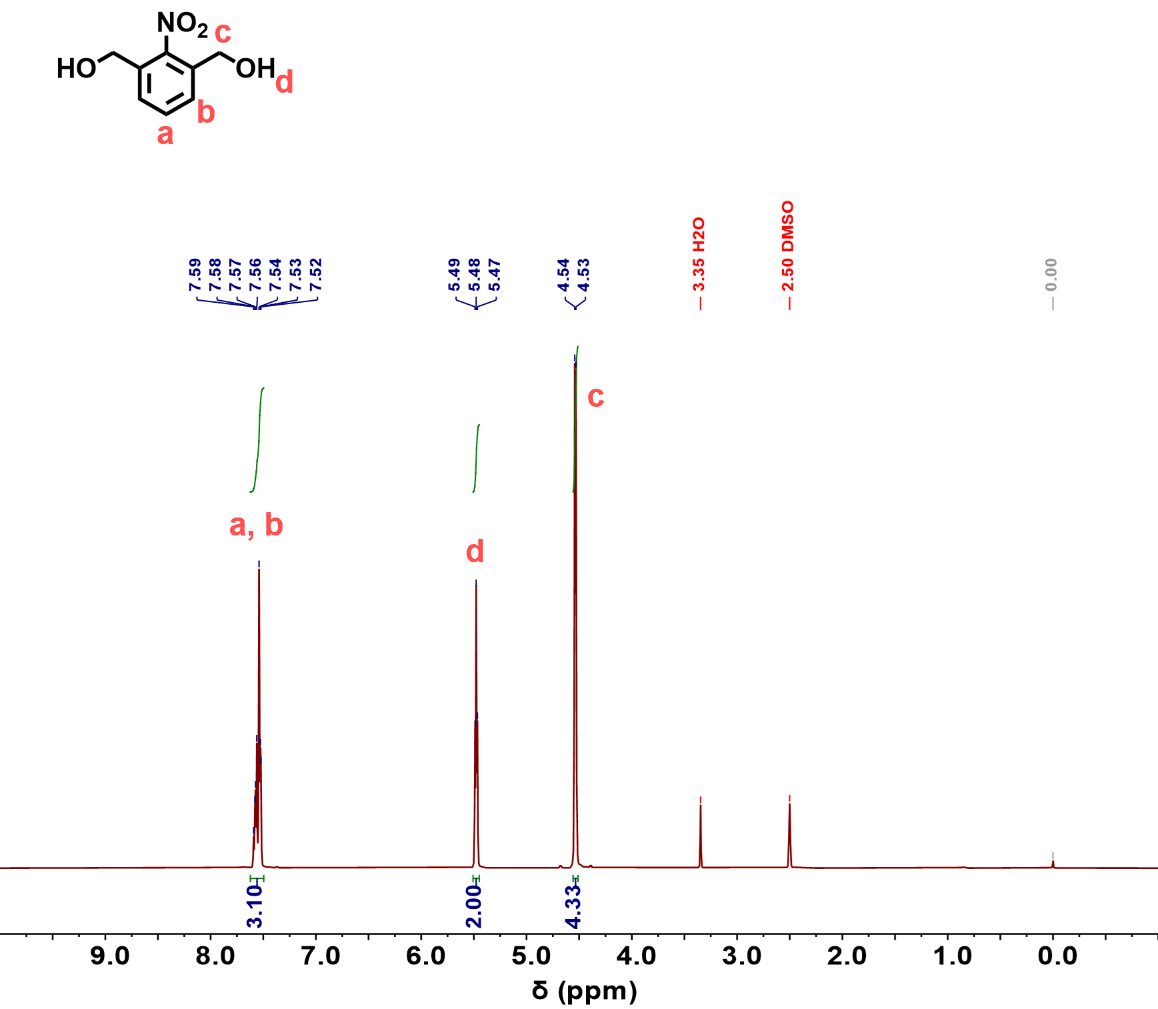


**Figure S1.** ^1^H NMR spectrum of (2-nitrobenzene-1,3-diyl)dimethanol (**1**) in DMSO-*d*6.


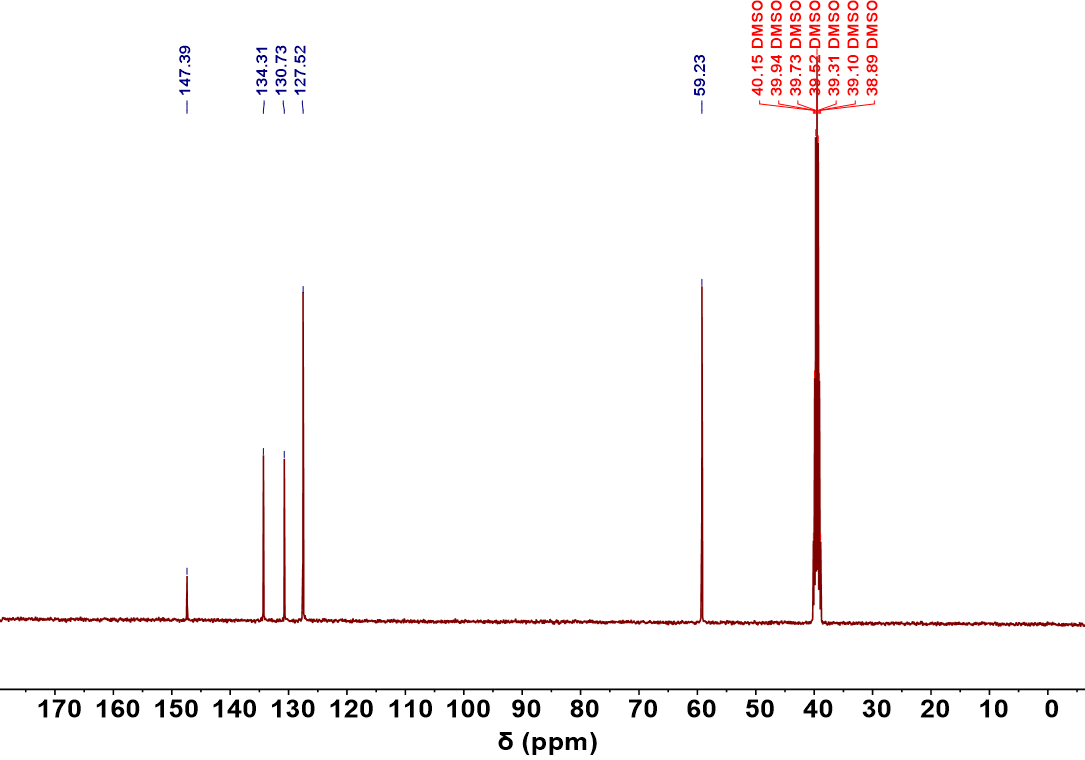


**Figure S2.** ^13^C NMR spectrum of (2-nitrobenzene-1,3-diyl)dimethanol (**1**) in DMSO-*d*6.


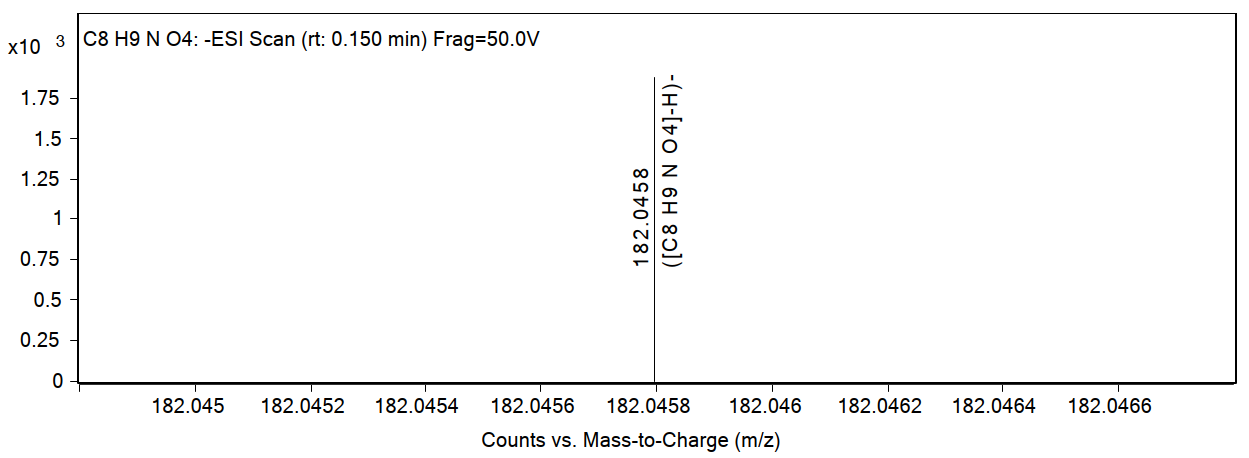


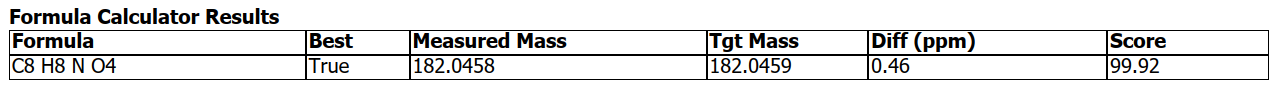


**Figure S3.** HRMS spectrum of (2-nitrobenzene-1,3-diyl)dimethanol (**1**).


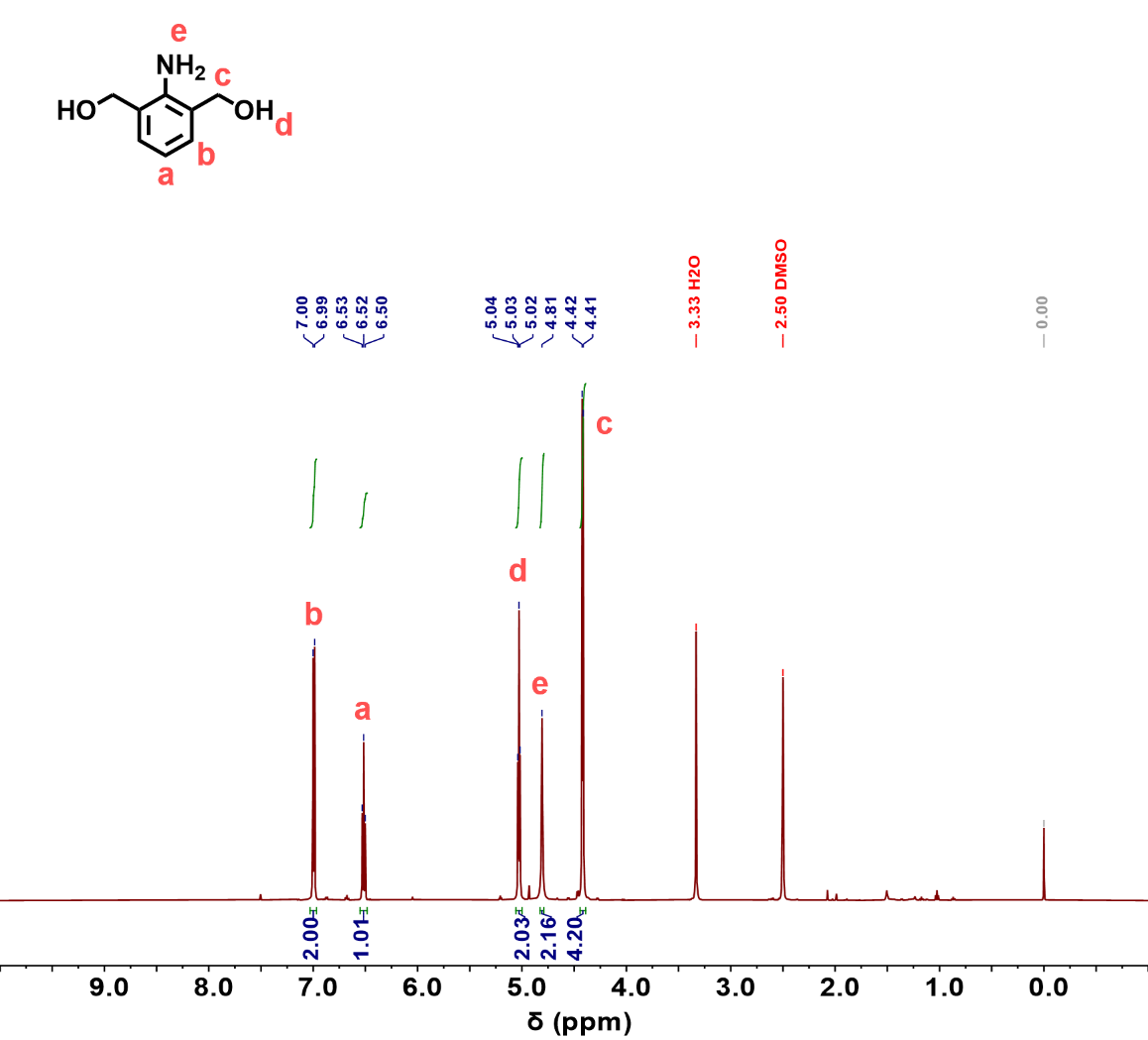


**Figure S4.** ^1^H NMR spectrum of 2,6-bis(hydroxymethyl)aniline (BHA, **2**) in DMSO-*d*6.


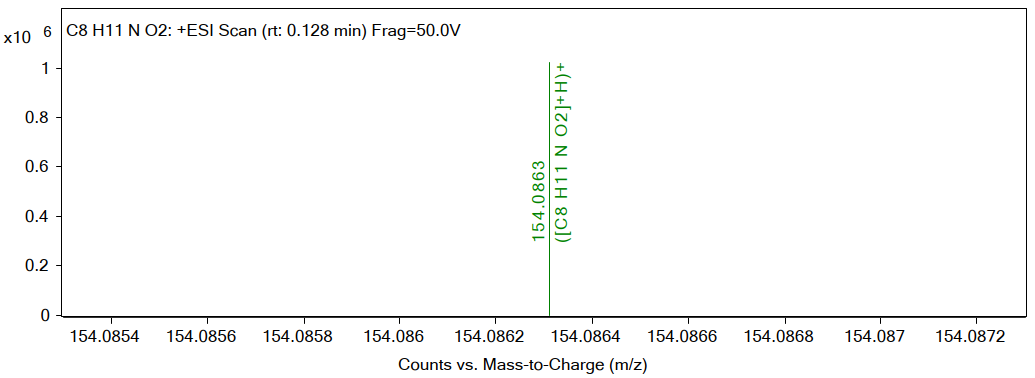


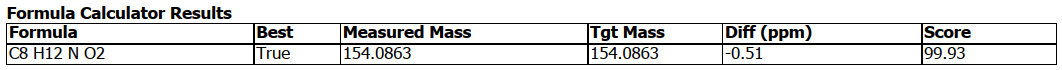


**Figure S5.** HRMS spectrum of 2,6-bis(hydroxymethyl)aniline (BHA, **2**).


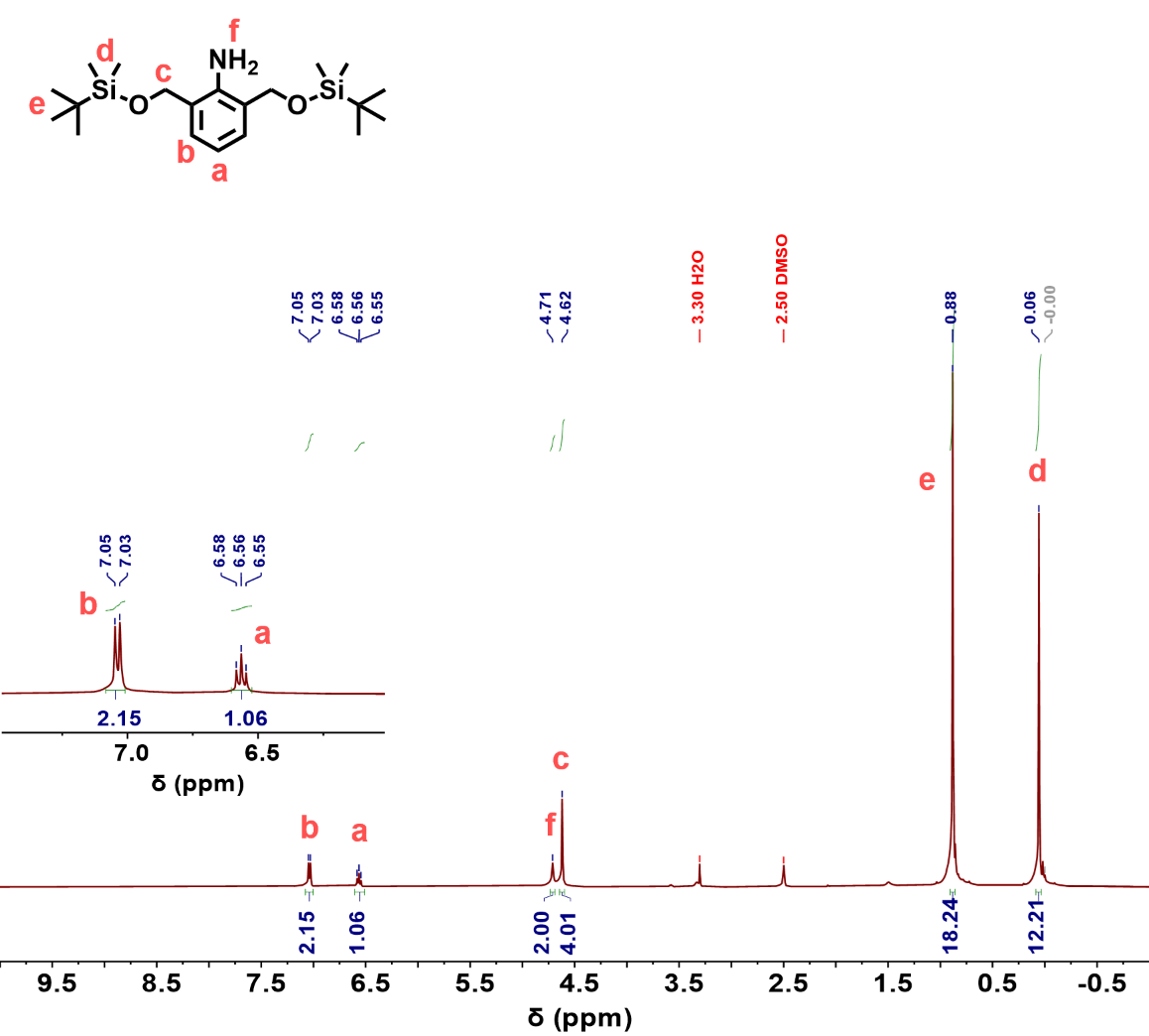


**Figure S6.** ^1^H NMR spectrum of 2,6-bis[(*tert*-butyldimethylsilyloxy)methyl]aniline (BHA-OTBDMS_2_, **3**) in DMSO-*d*6.


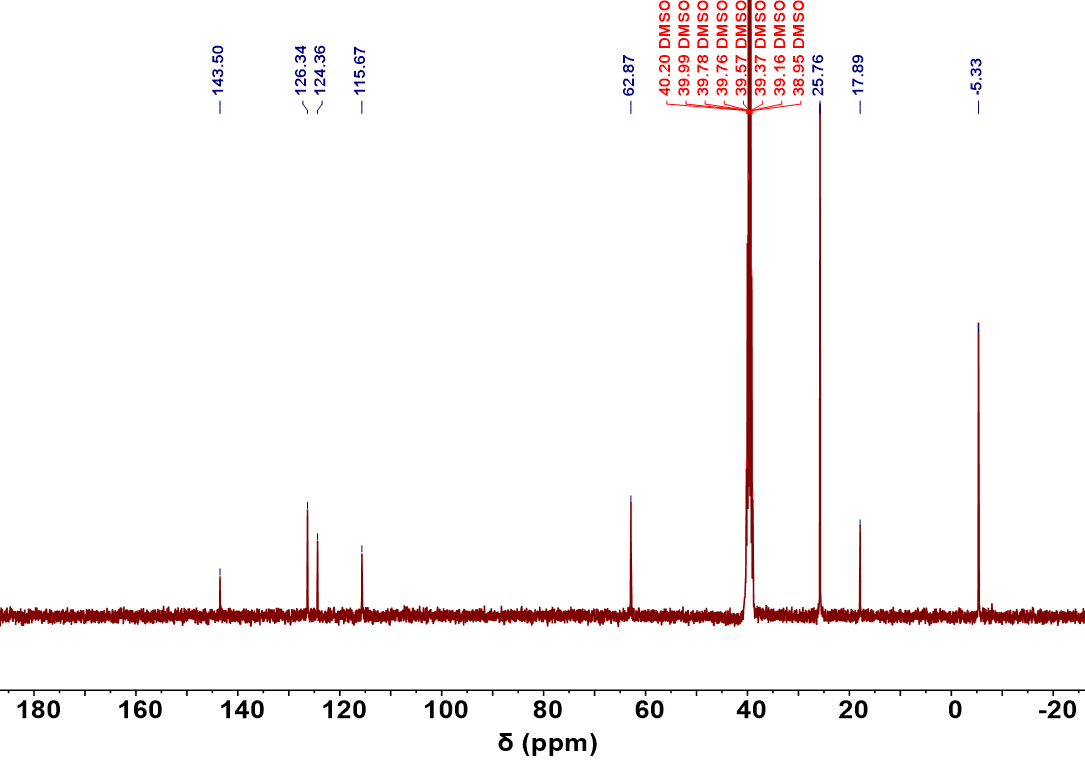


**Figure S7.** ^13^C NMR spectrum of 2,6-bis[(tert-butyldimethylsilyloxy)methyl]aniline (BHA-OTBDMS_2_, **3**) in DMSO-*d*6.


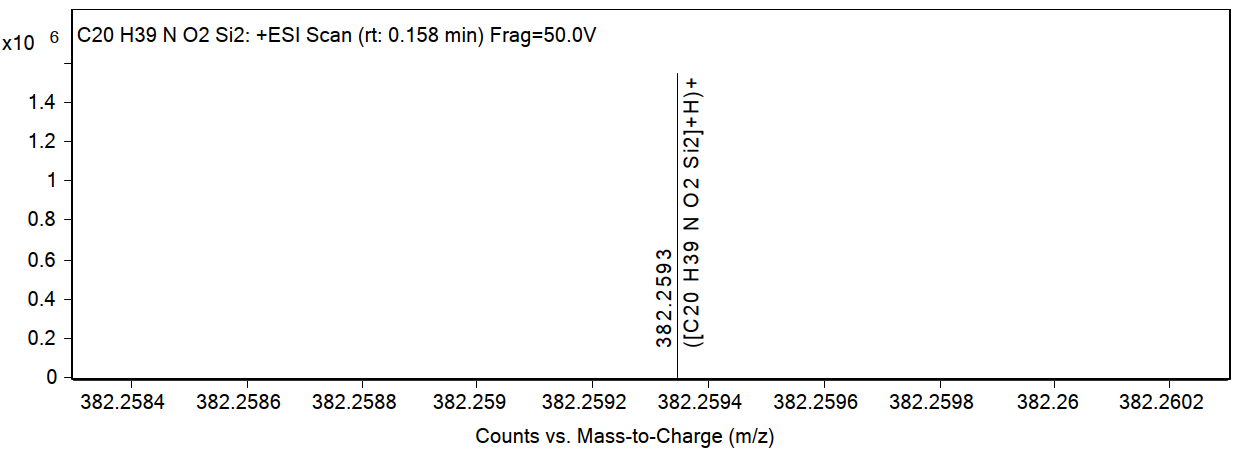


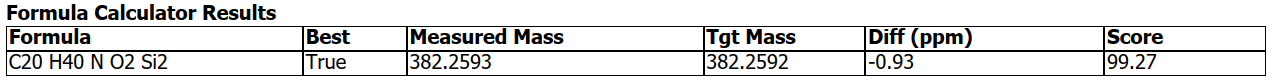


**Figure S8.** HRMS spectrum of 2,6-bis[(*tert*-butyldimethylsilyloxy)methyl]aniline (BHA-OTBDMS_2_, **3**).


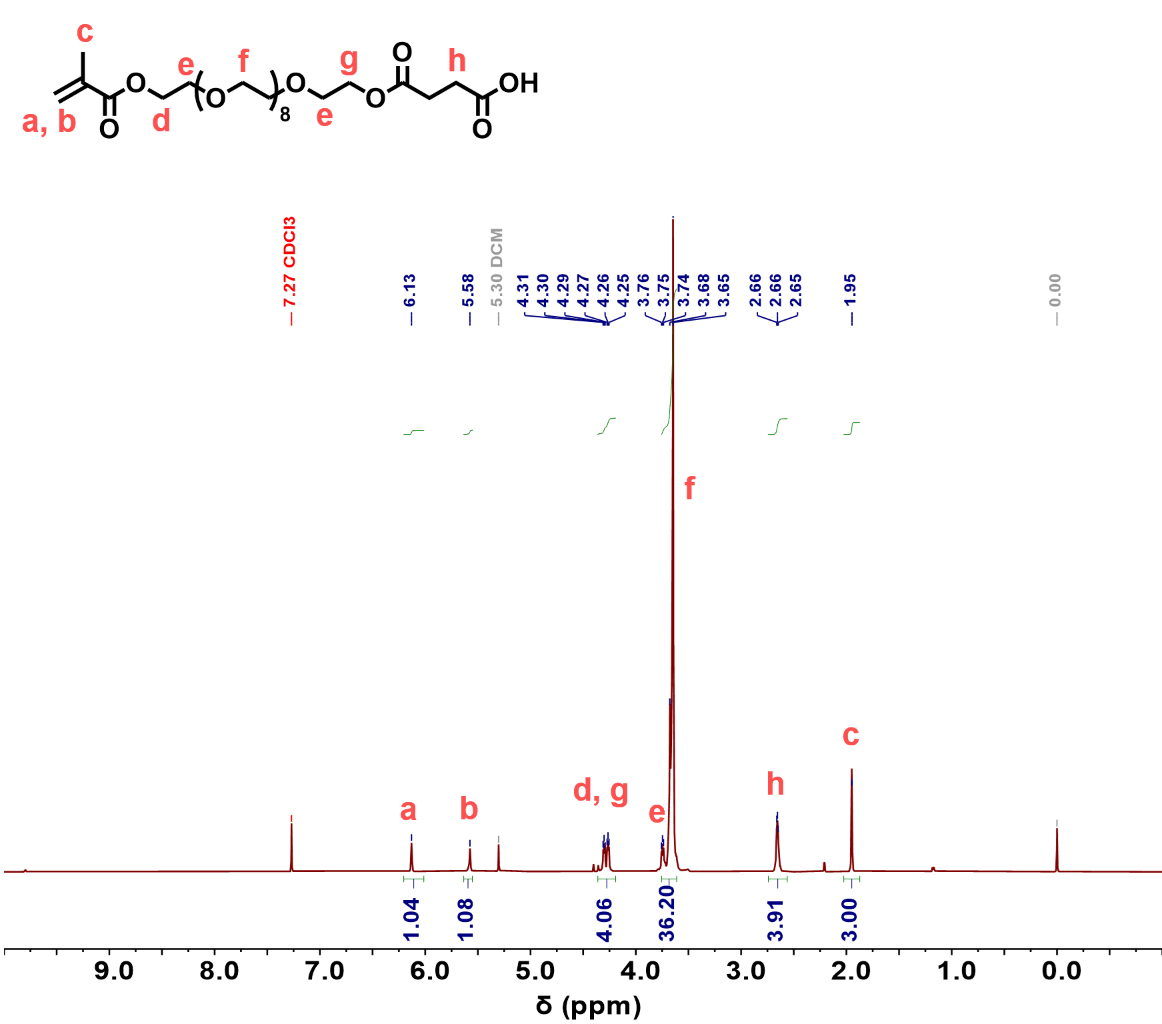


**Figure S9.** ^1^H NMR spectrum of succinic acid-modified methyl acrylate mono-oligethylene glycol ester (OEGMA-COOH, **4**) in CDCl_3_.


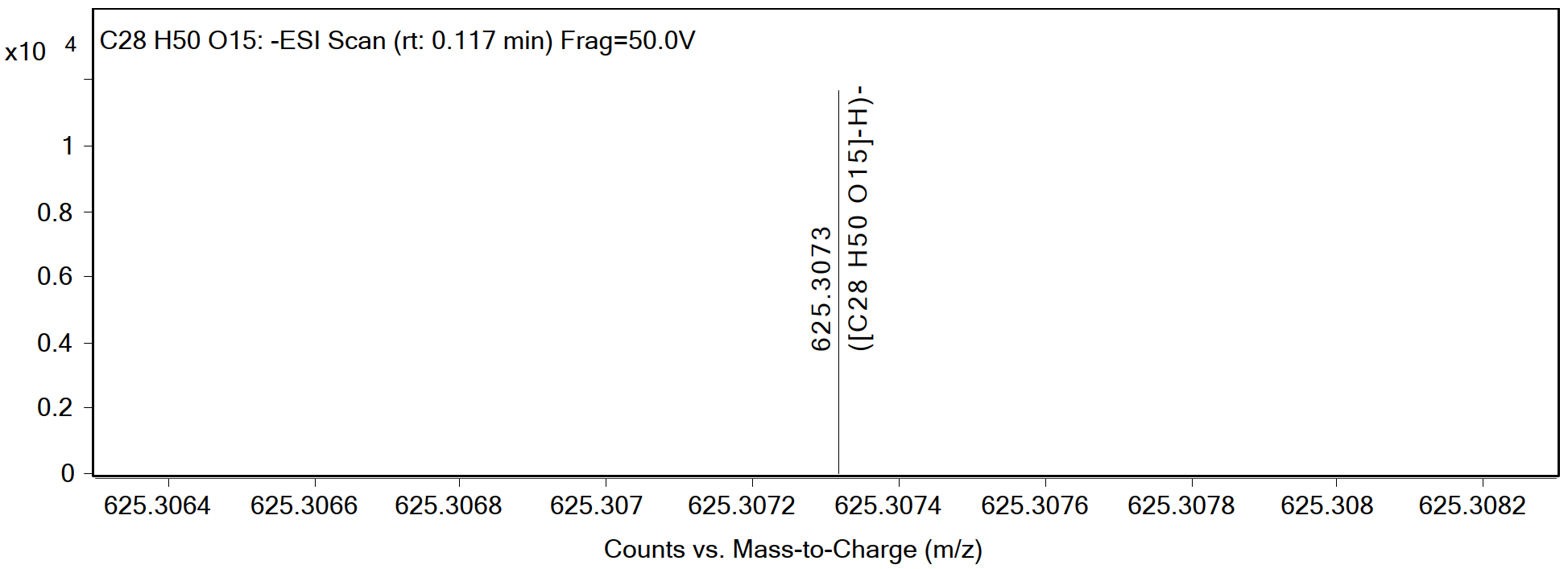


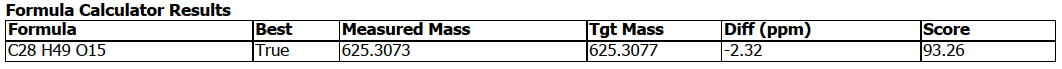


**Figure S10.** HRMS spectrum of succinic acid-modified methyl acrylate mono-oligethylene glycol ester (OEGMA-COOH, **4**).


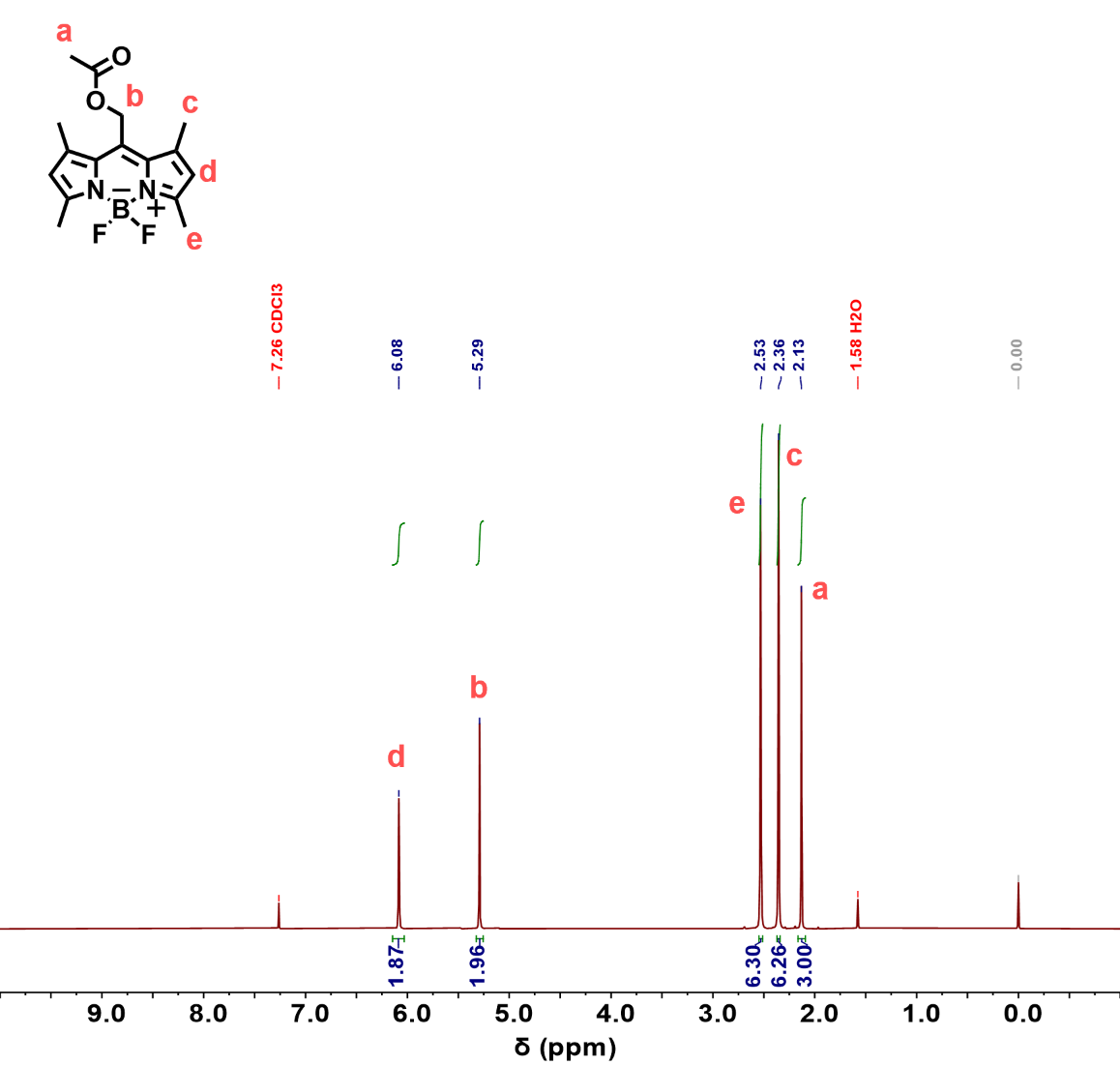


**Figure S11.** ^1^H NMR spectrum of 8-acetoxymethyl-1,3,5,7-tetramethyl pyrromethene fluoroborate (BODIPY-OAc, **5**) in CDCl_3_.


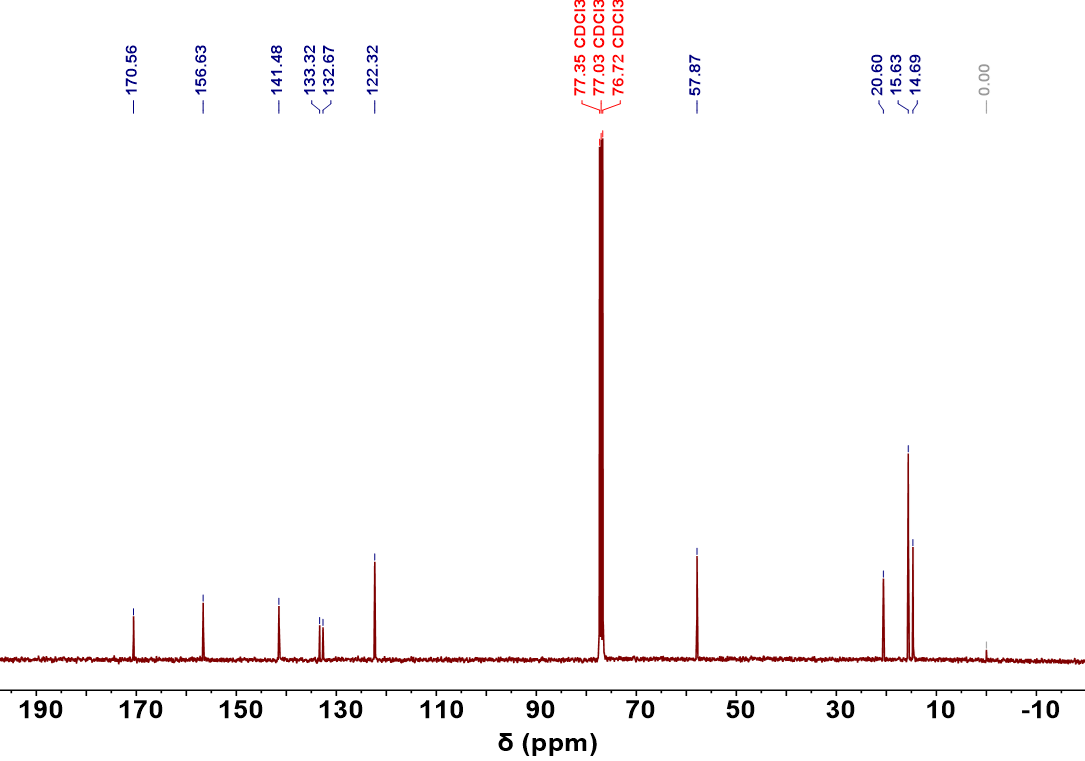


**Figure S12** ^13^C NMR spectrum of 8-acetoxymethyl-1,3,5,7-tetramethyl pyrromethene fluoroborate (BODIPY-OAc, **5**) in CDCl_3_.


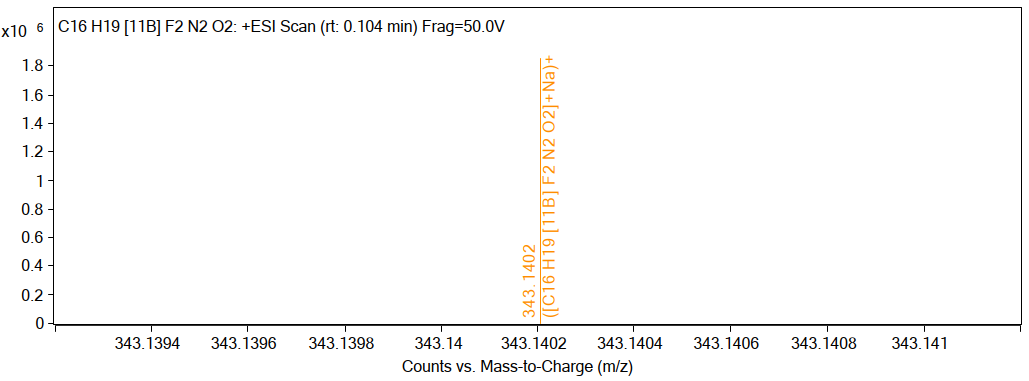


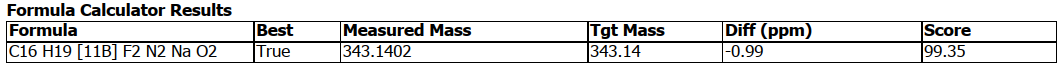


**Figure S13** HRMS spectrum of 8-acetoxymethyl-1,3,5,7-tetramethyl pyrromethene fluoroborate (BODIPY-OAc, **5**).


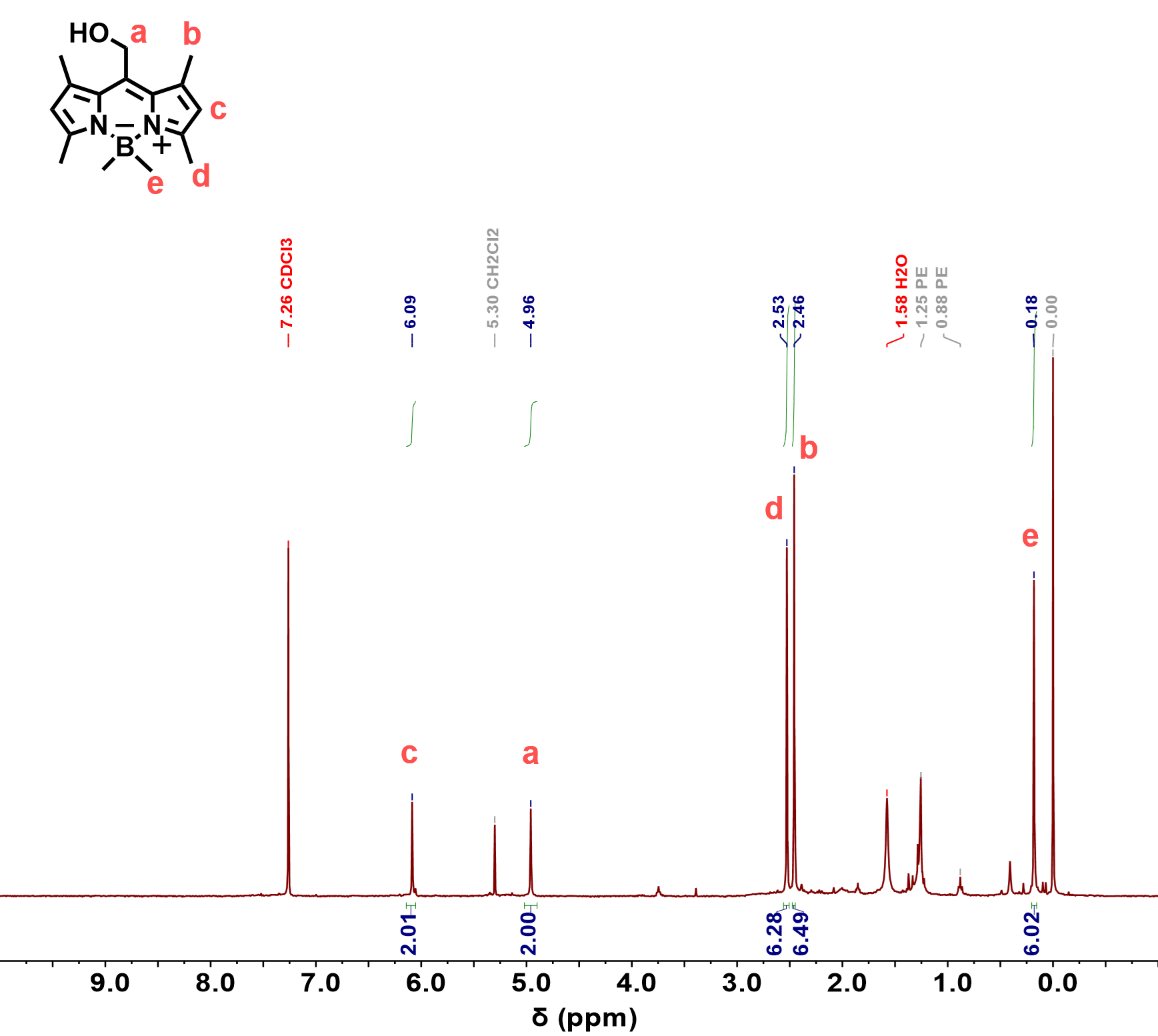


**Figure S14** ^1^H NMR spectrum of 4,4′-dimethyl-8-hydroxymethyl-1,3,5,7-tetramethyl-4-bora-3*a*,4*a*-diaza-*s*-indacene (DM-BODIPY-OH, **6**) in CDCl_3_.


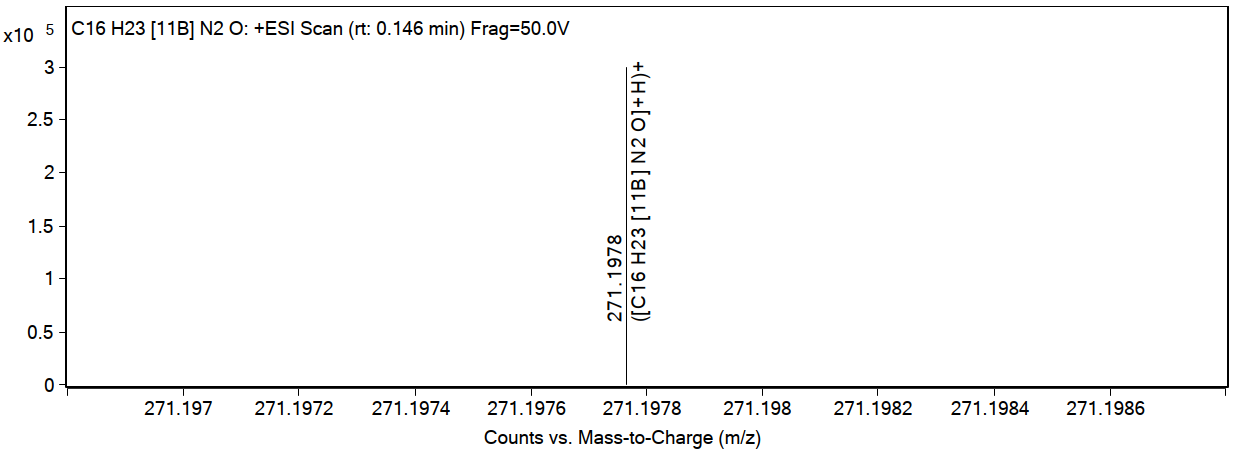


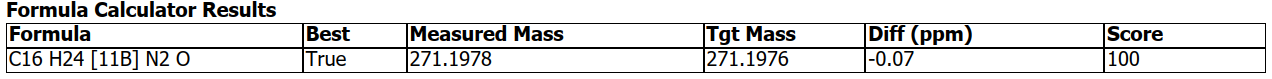


**Figure S15** HRMS spectrum of 4,4′-dimethyl-8-hydroxymethyl-1,3,5,7-tetramethyl-4-bora-3*a*,4*a*-diaza-*s*-indacene (DM-BODIPY-OH, **6**).


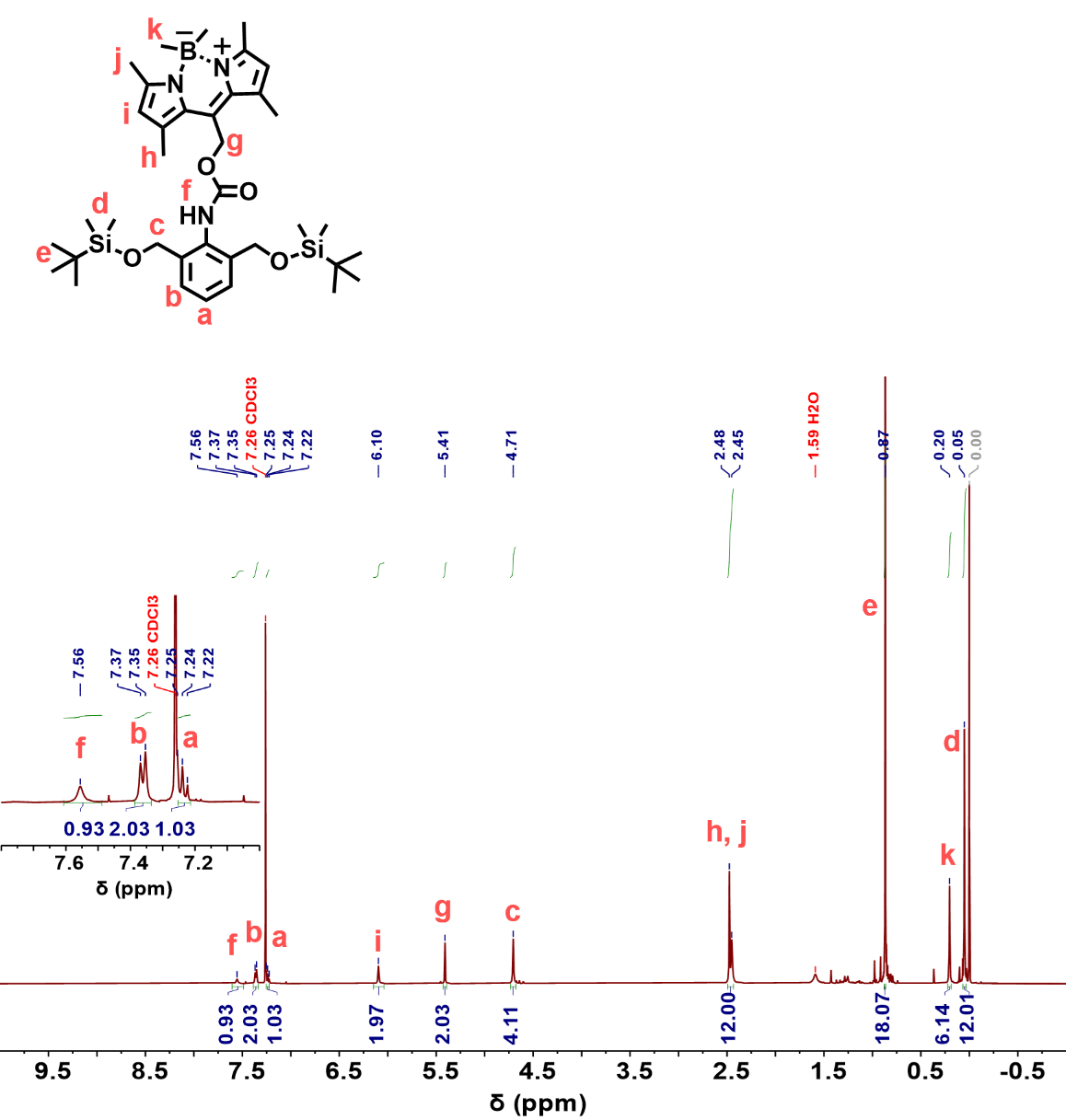


**Figure S16** ^1^H NMR spectrum of DM-BODIPY-BHA-OTBDMS_2_ (**7**) in CDCl_3_.


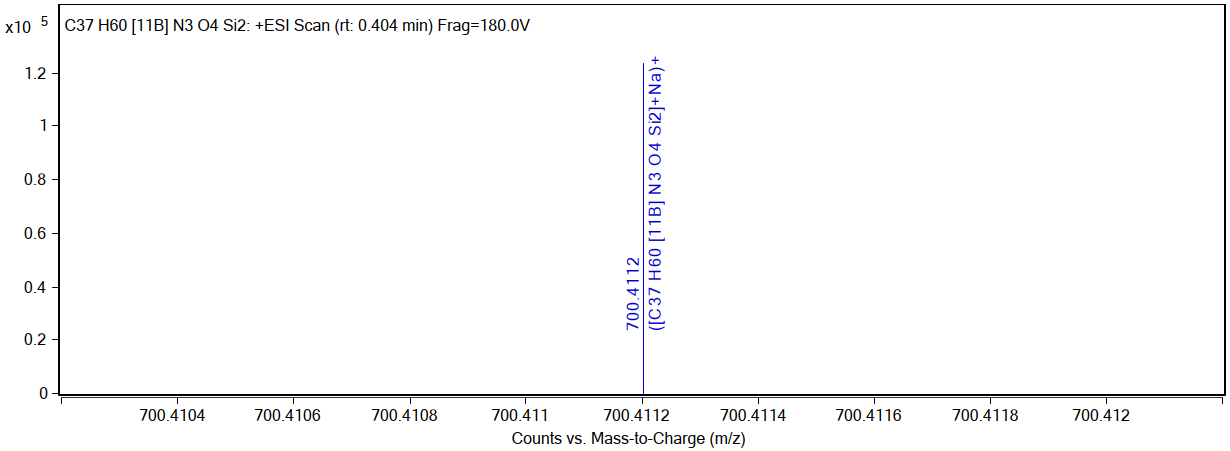


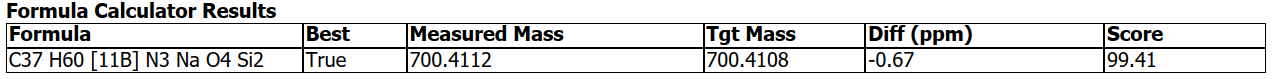


**Figure S17** HRMS spectrum of DM-BODIPY-BHA-OTBDMS_2_ (**7**).


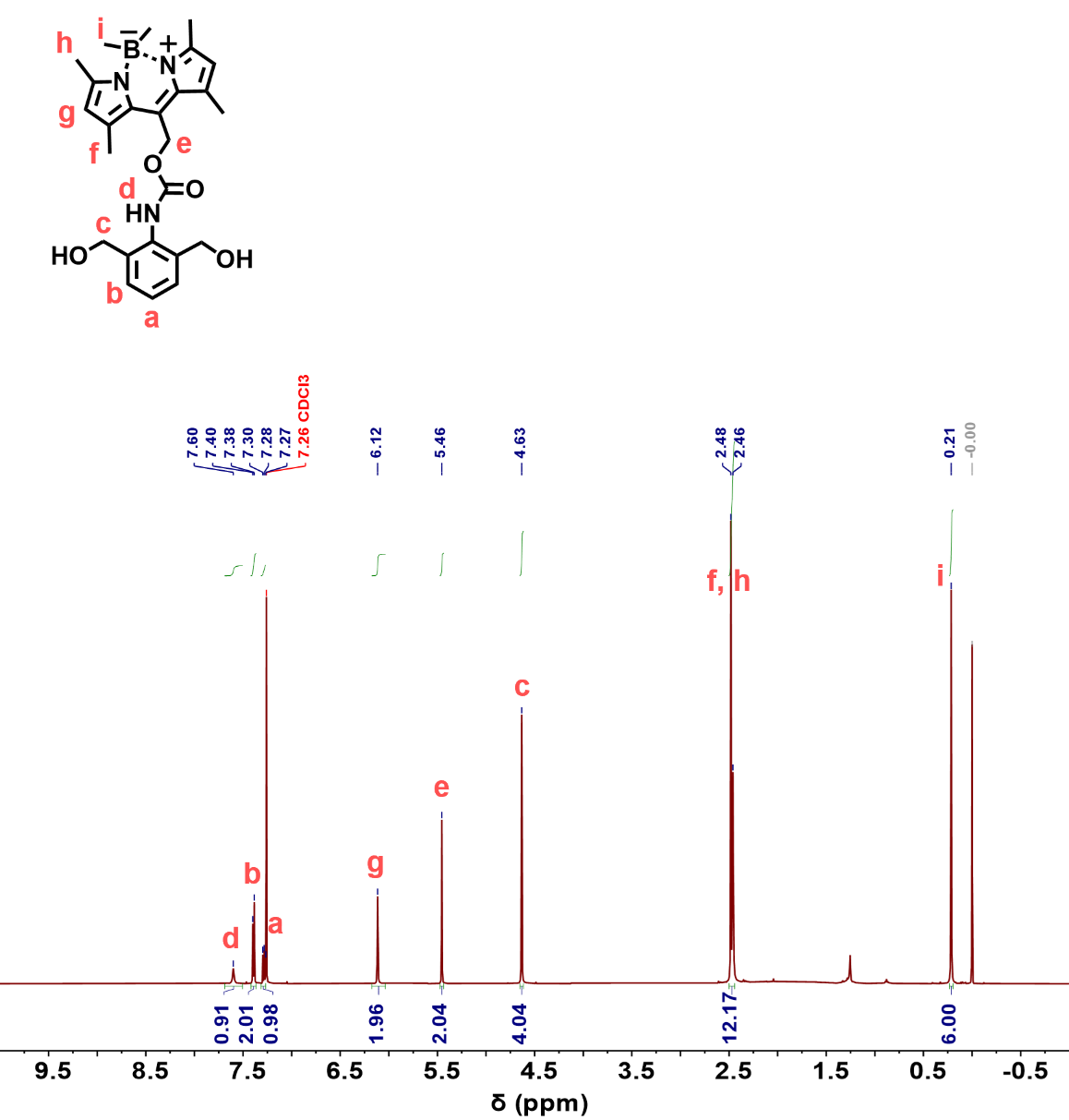


**Figure S18** ^1^H NMR spectrum of DM-BODIPY-BHA-OH_2_ (**8**) in CDCl_3_.


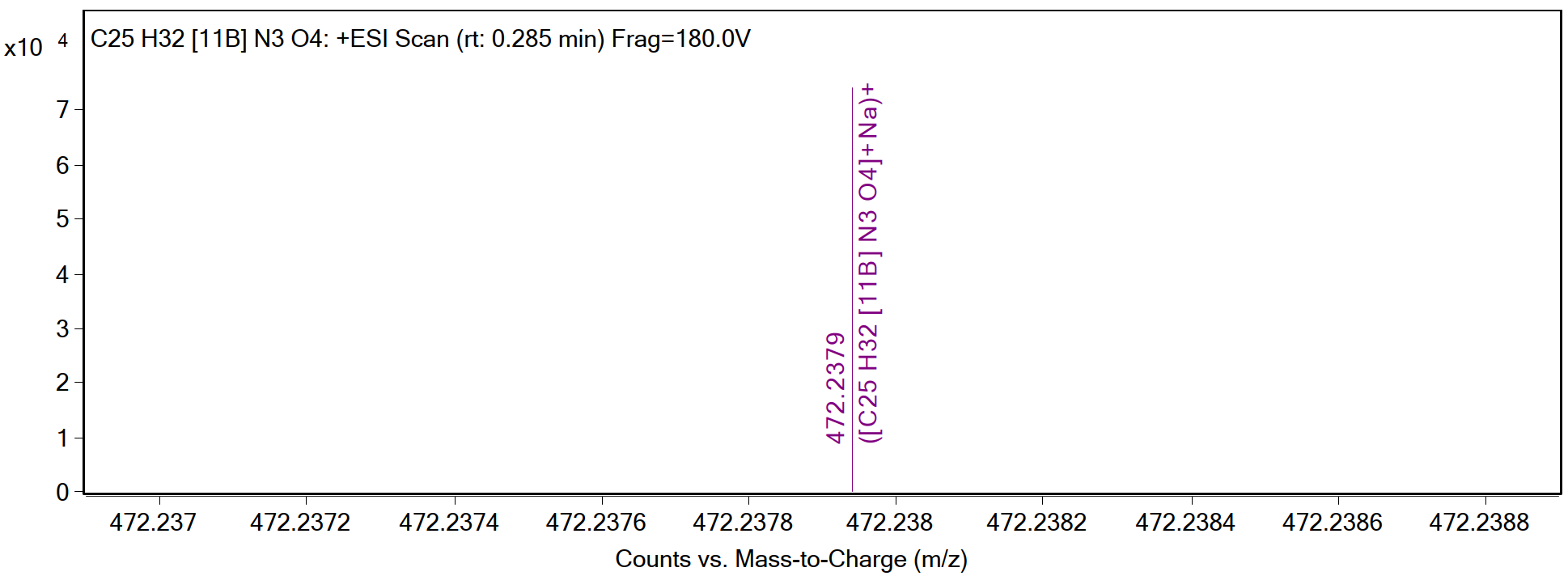


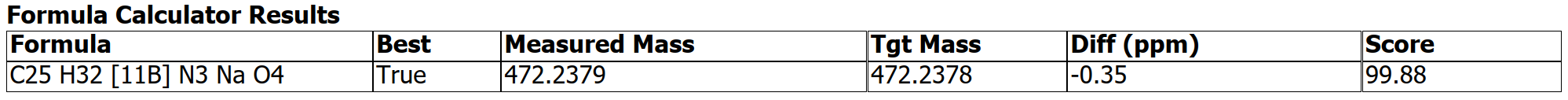


**Figure S19.** HRMS spectrum of DM-BODIPY-BHA-OH_2_ (**8**).


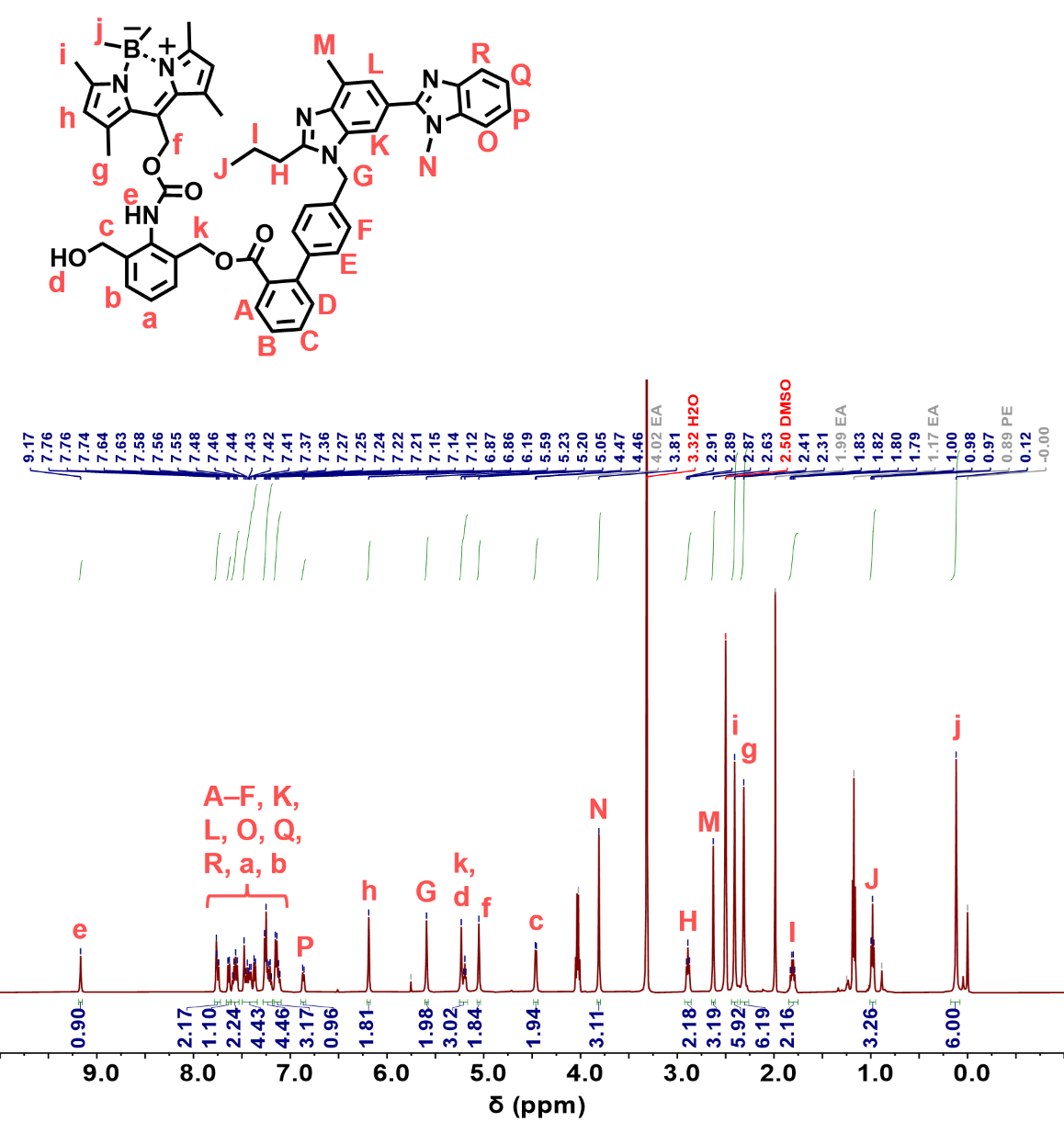


**Figure S20.** ^1^H NMR spectrum of DM-BODIPY-BHA-OH-Tel (GTel, **9**) in DMSO-*d*6.


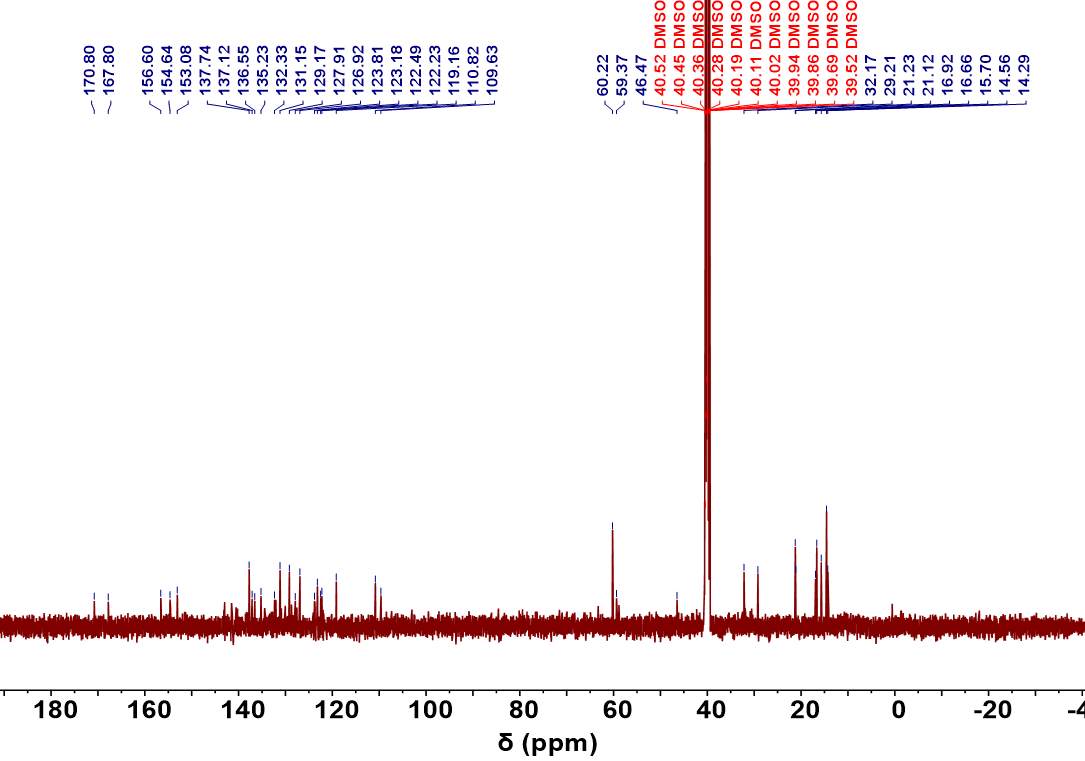


**Figure S21.** ^13^C NMR spectrum of DM-BODIPY-BHA-OH-Tel (GTel, **9**) in DMSO-*d*6.


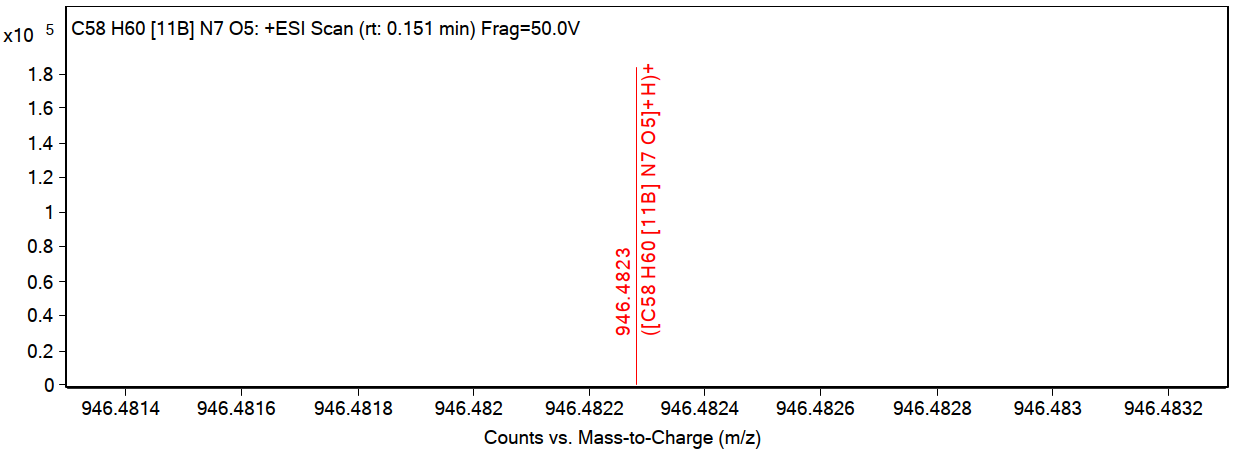


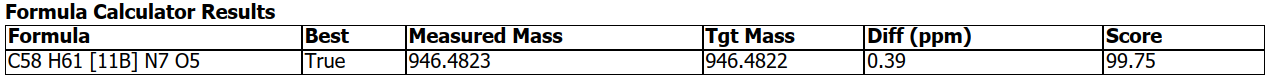


**Figure S22.** HRMS spectrum of DM-BODIPY-BHA-OH-Tel (GTel, **9**).


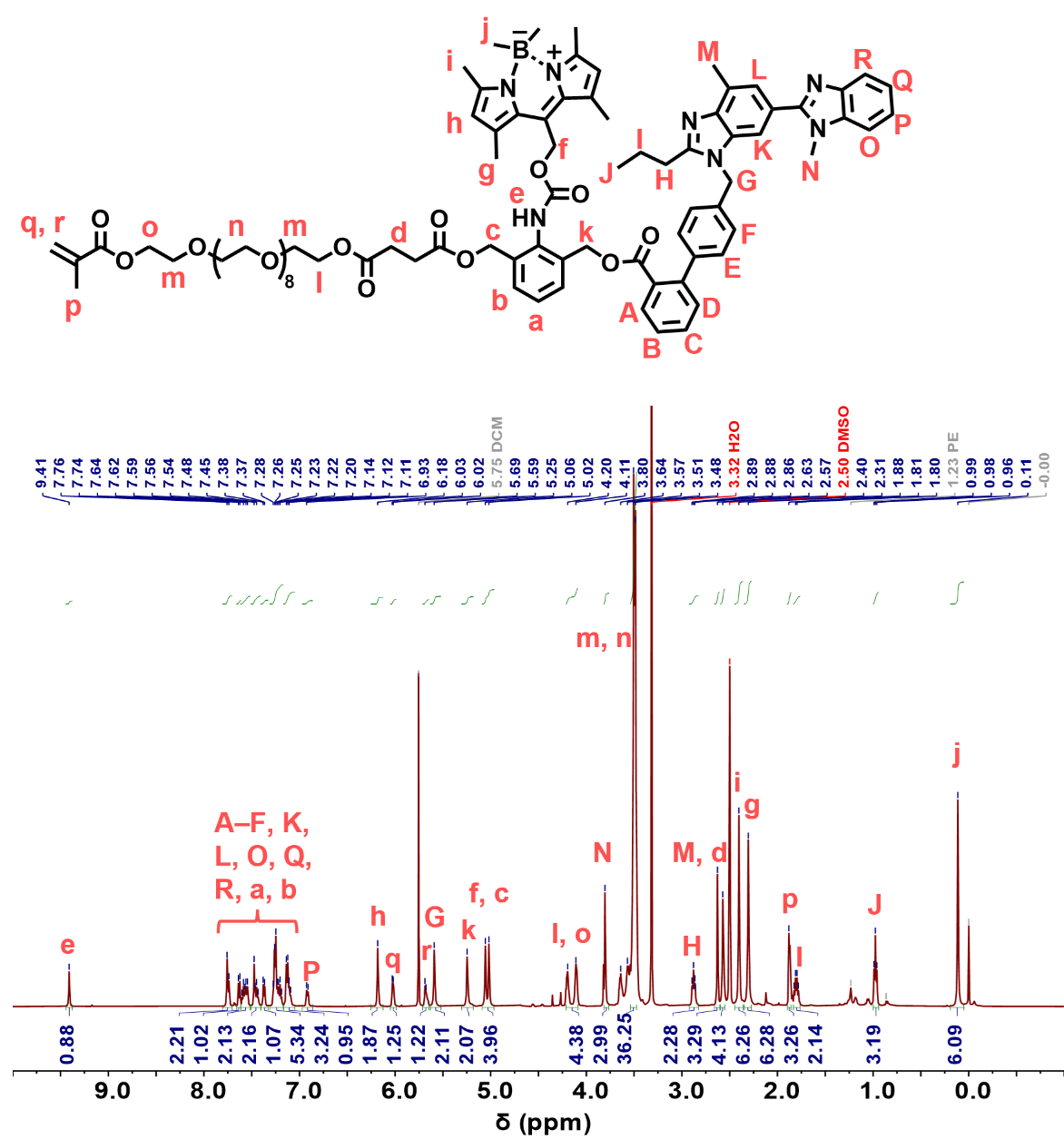


**Figure S23.** ^1^H NMR spectrum of DM-BODIPY-BHA-OEGMA-Tel (GTel-mono, **10**) in DMSO-*d*6.


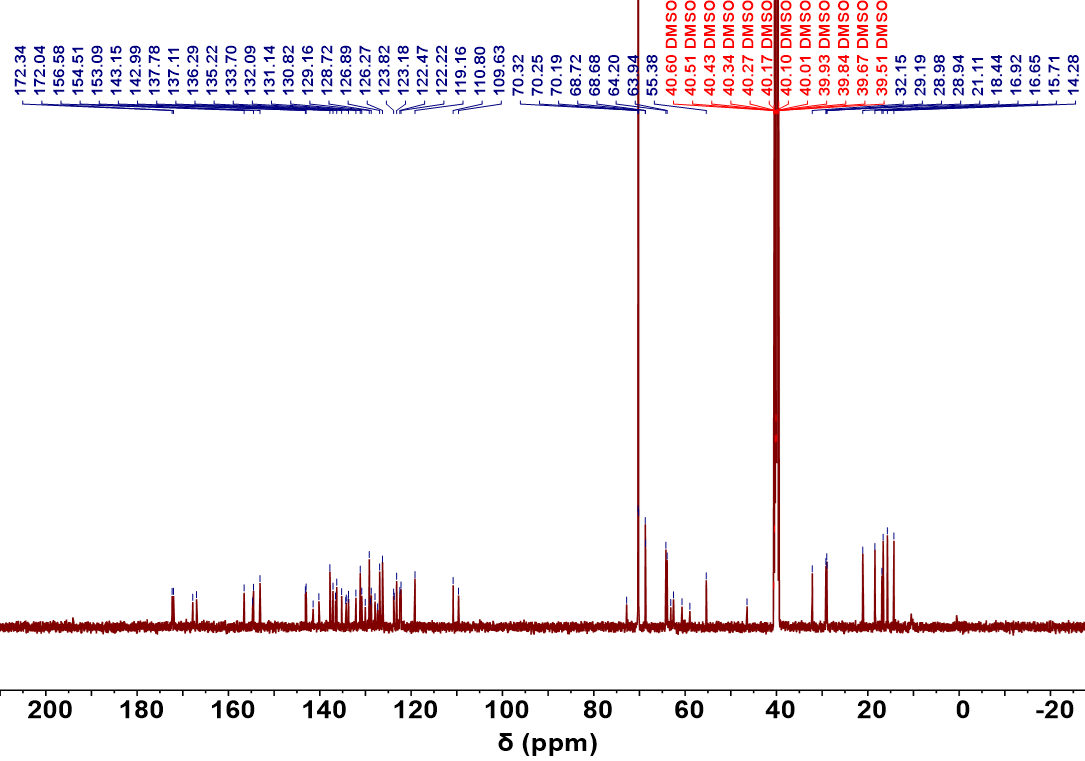


**Figure S24** ^13^C NMR spectrum of DM-BODIPY-BHA-OEGMA-Tel (GTel-mono, **10**) in DMSO-*d*6.


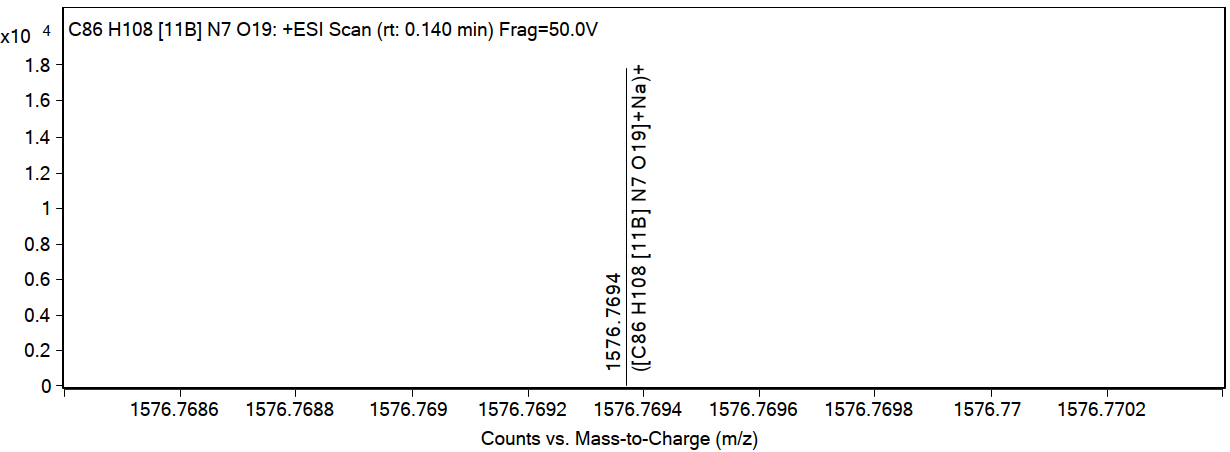


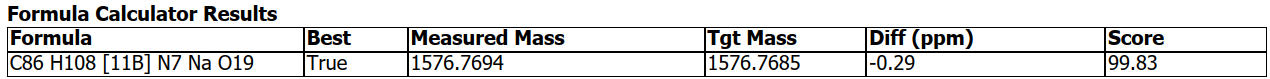


**Figure S25.** HRMS spectrum of DM-BODIPY-BHA-OEGMA-Tel (GTel-mono, **10**).


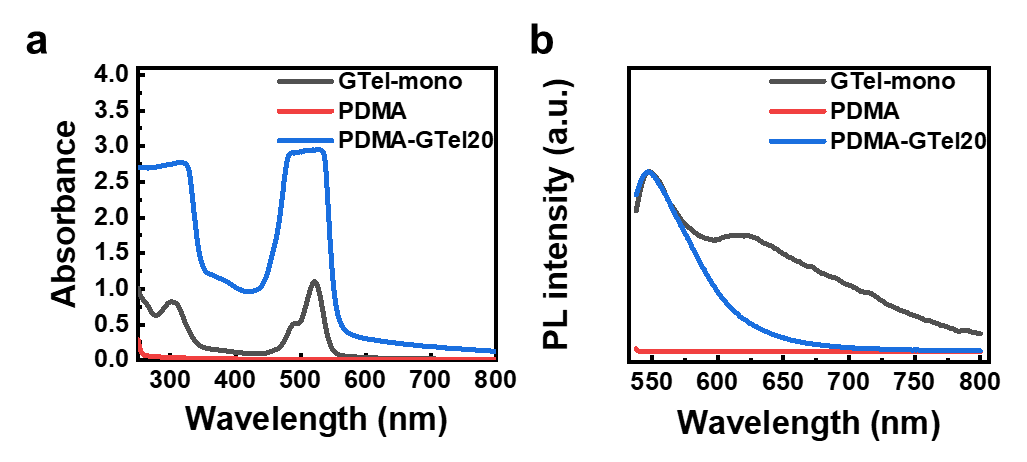


**Figure S26** (a) UV–vis absorption ang (b) PL emission (*λ*_ex_ = 520 nm) spectra of the GTel-mono, blank PDMA hydrogel, and PDMA-GTel20 hydrogel.


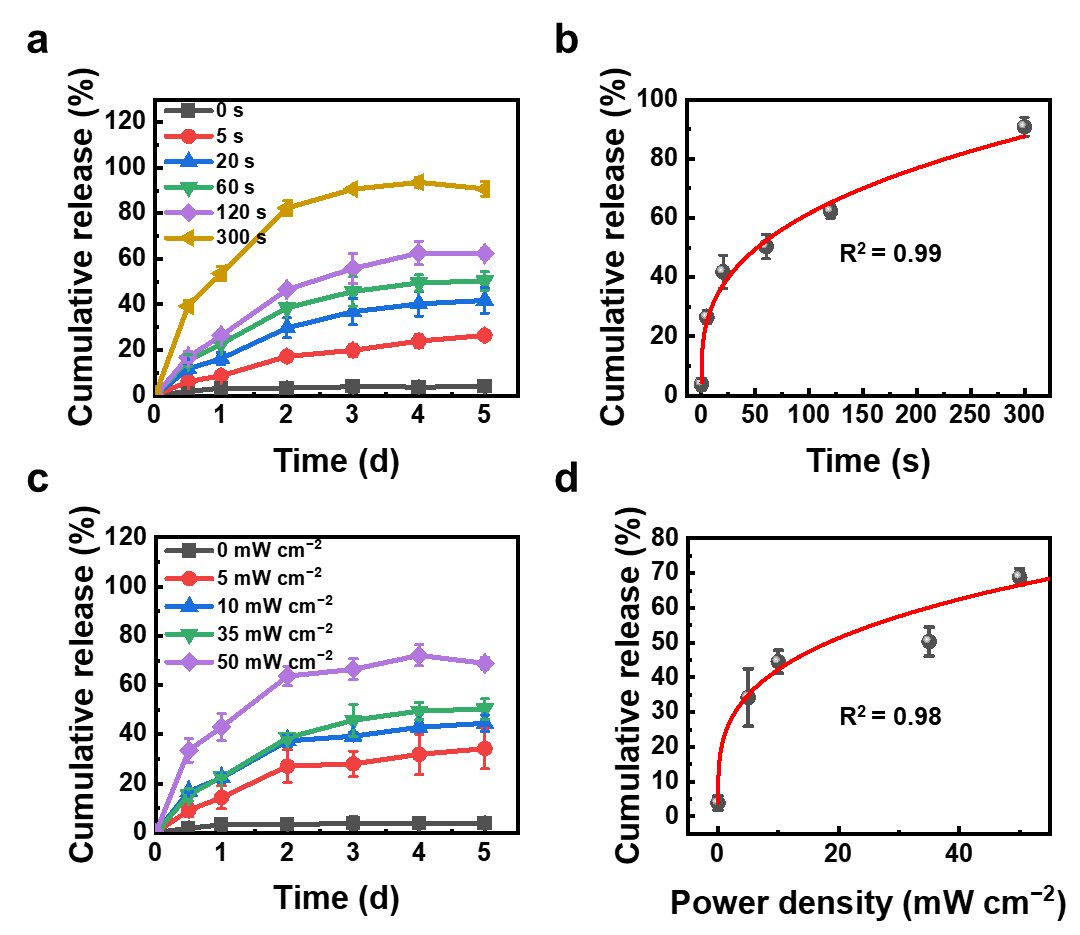


**Figure S27** Light dose‑dependent drug release from PDMA‑GTel20 hydrogel. (a) Cumulative release profiles under fixed LED power density (35 mW cm^−2^) with varying exposure times (5, 20, 60, 120, 300 s). A negative control (no light) is included. (b) Calibration curve of cumulative release (5‑day) versus exposure time. Data are fitted with a four-parameter logistic model (*y* = A2 + (A1 − A2)/(1 + (*x*/*x*_0_)^*p*), A1 = 4.42626, A2 = 22393.84652, *x*_0_ = 3.50642 × 10^9^, *p* = 0.34361, R^2^ = 0.99). (c) Cumulative release profiles under fixed exposure time (1 min) with varying power density (5, 10, 35, 50 mW cm^−2^). Negative control (0 mW cm^−2^) is included. (d) Calibration curve of cumulative release (5‑day) versus power density. Data are fitted with a four-parameter logistic model (*y* = A2 + (A1 − A2)/(1 + (*x*/*x*_0_)^*p*), A1 = 3.9288, A2 = 3994.42298, *x*_0_ = 3.31488 × 10^7^, *p* = 0.30871, R^2^ = 0.98). Data are presented as mean ± SD (*n* = 3).


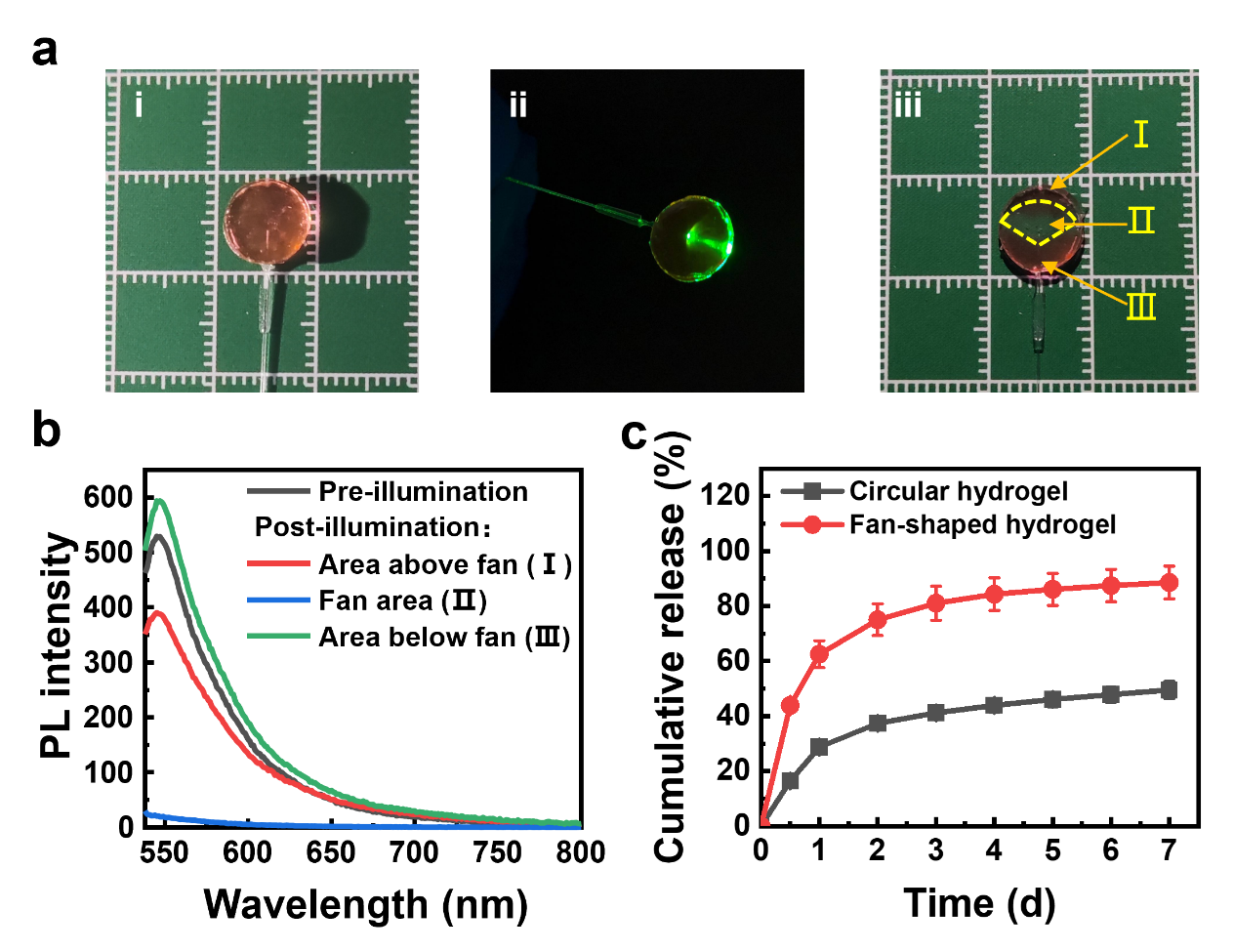


**Figure S28.** Light emission distribution characteristics of iPDP device and region-restricted drug release. (a) Photograph of iPDP device integrated with circular PDMA-GTel20 hydrogel: (ⅰ) no-light-emission state (1 × 1 cm grid background), (ⅱ) active-light-emission state (520 nm laser input via optical fiber), (ⅲ) post-sustained-emission state (after 1 h continuous light emission; yellow dashed fan: r = 4 mm, center 5 mm from top and 3 mm from bottom). (b) Regional PL emission spectral analysis of circular PDMA-GTel20 hydrogel (corresponding to a.ⅲ) (c) Cumulative release of circular and fan-shaped PDMA-GTel20 hydrogel under iPDP confined light emission. Duration: 1 h, operating current: 1440 mA. Data are presented as mean ± SD (*n* = 3).

**Figure S29**. Comparison of release kinetics and storage stability between GTel10 and PDMA‑GTel20 hydrogels. Red lines: release under illumination (520 nm LED, 50 mW cm^−2^, 20 min); black lines: baseline leakage under dark conditions (storage stability). Darker lines represent GTel10; lighter lines represent PDMA‑GTel20. Data are presented as mean ± SD (n = 3).


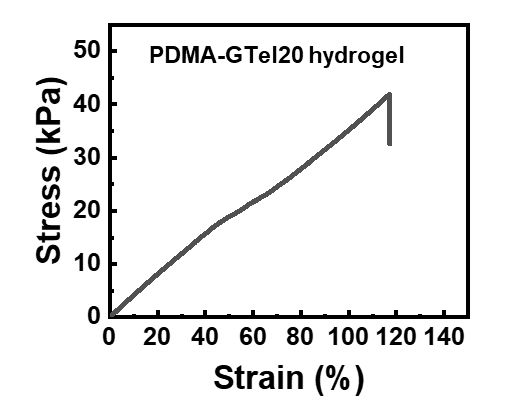


**Figure S30.** Tensile stress-strain curve of the PDMA-GTel20 hydrogel. The test was performed at 25 °C using dumbbell-shaped specimens (gauge length *L*_0_ = 10 mm, width of narrow section *W* = 2 mm, thickness = 1 mm) at a crosshead speed of 10 mm min^−1^. A representative curve is shown.


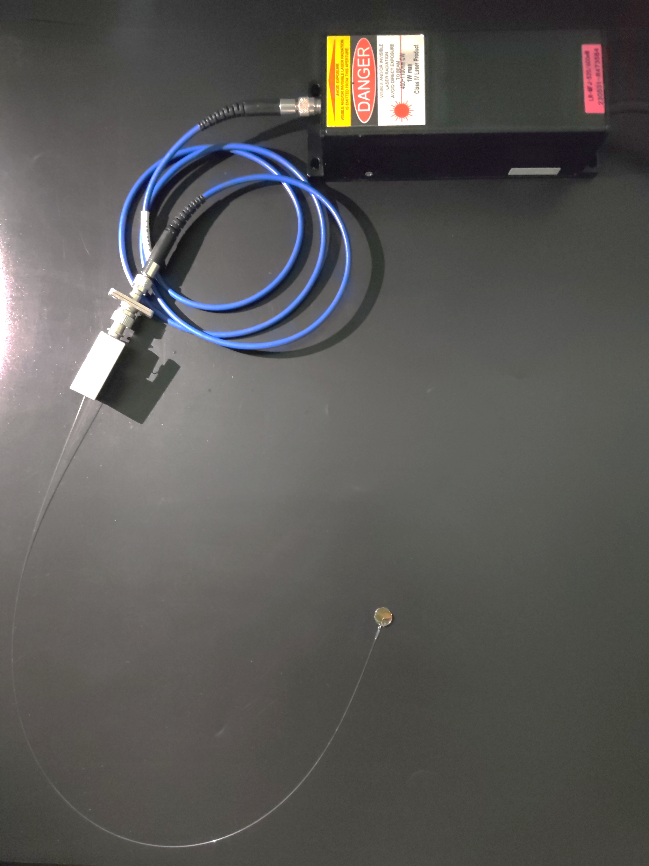


**Figure S31.** Optical connection of iPDP device. Photograph of iPDP device connected to 520 nm laser.

**Figure S32** Comparison of iPDP release profiles in 0.5% Tween and 10% serum media under different triggering conditions. Release tests were performed with the hydrogel remained attached to the PDMS disc throughout the incubation. Three conditions were tested: (i) single trigger (35 mW cm^−2^, 60 min), (ii) triple‑trigger regimen (days 0, 2, 4; parameters as in **Figure 3f**), and (iii) no‑light control (baseline leakage). All data are presented as cumulative release percentage over 7 days. Data are presented as mean ± SD (*n* = 3).


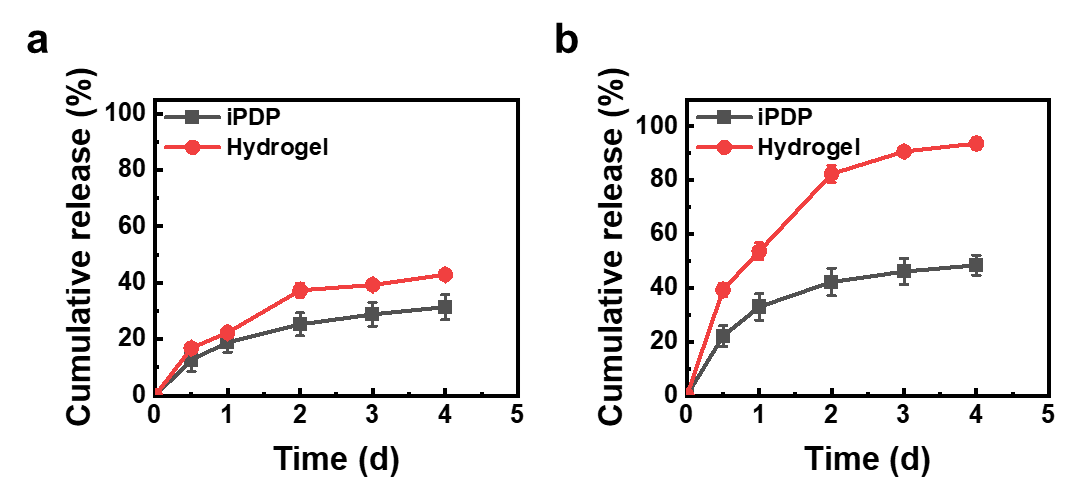


**Figure S33**. Comparison of cumulative drug release (4‑day) from PDMA‑GTel20 hydrogel alone and from the complete iPDP device under identical illumination conditions. The hydrogel alone was tested using the LED source (same as in hydrogel characterization), while the iPDP device was illuminated via its integrated optical fiber (520 nm laser). Two illumination conditions were applied: (a) 10 mW cm^−2^ for 1 min, and (b) 35 mW cm^−2^ for 5 min. Data are presented as mean ± SD (*n* = 3).


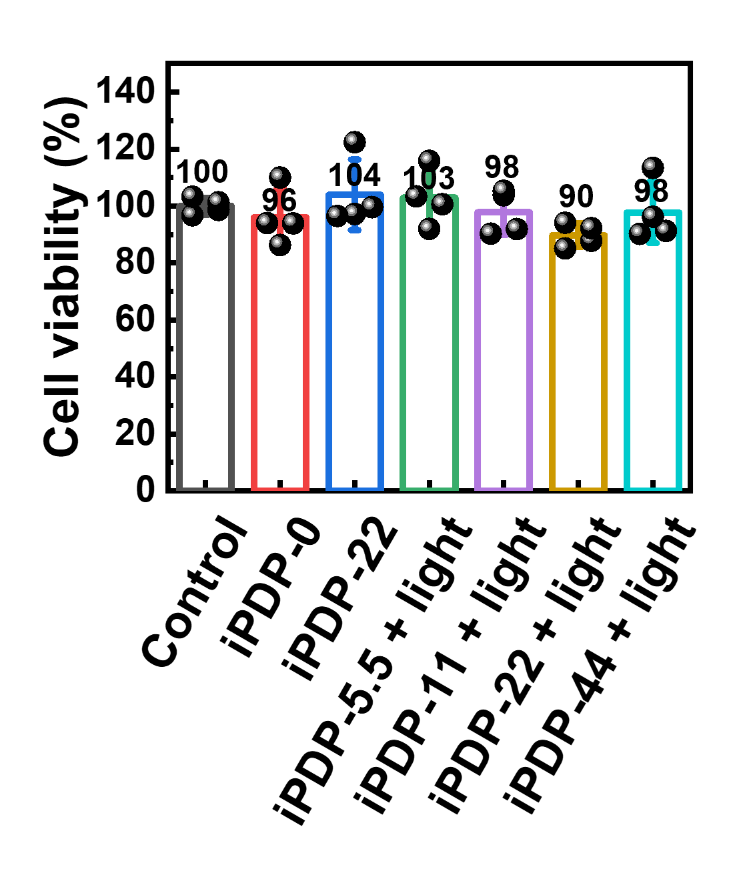


**Figure S34.** In vitro cytotoxicity evaluation of the iPDP device. Cell viability of H9c2 cardiomyocytes was assessed using a CCK-8 assay after 72 h of co-culture with the device in a Transwell system. Groups are as follows: Control (cells only); iPDP-0 (device with a drug-free blank hydrogel, without irradiation); iPDP-22 (device with a prodrug hydrogel containing approximately 22 μg telmisartan, without irradiation); and devices with pre-irradiated hydrogels at graded drug loads (iPDP-5.5 + light, iPDP-11 + light, iPDP-22 + light, iPDP-44 + light, where the number indicates the approximate loaded telmisartan mass in μg). Data are presented as mean ± SD (*n* = 4).


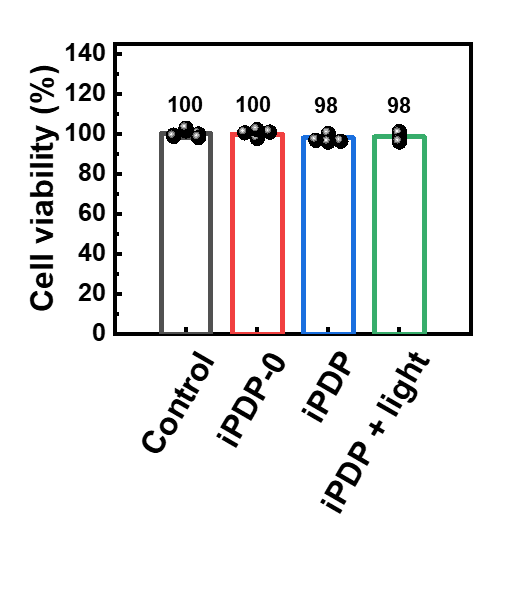


**Figure S35**. CCK‑8 cytotoxicity assay of the iPDP device (PDMA‑GTel20) using a Transwell co‑culture system. Groups: control (cells alone), iPDP‑0 (PDMA disc with PDMA), iPDP (PDMA disc with PDMA‑GTel20), and iPDP + light (device irradiated after placement). Data are presented as mean ± SD (n = 4).


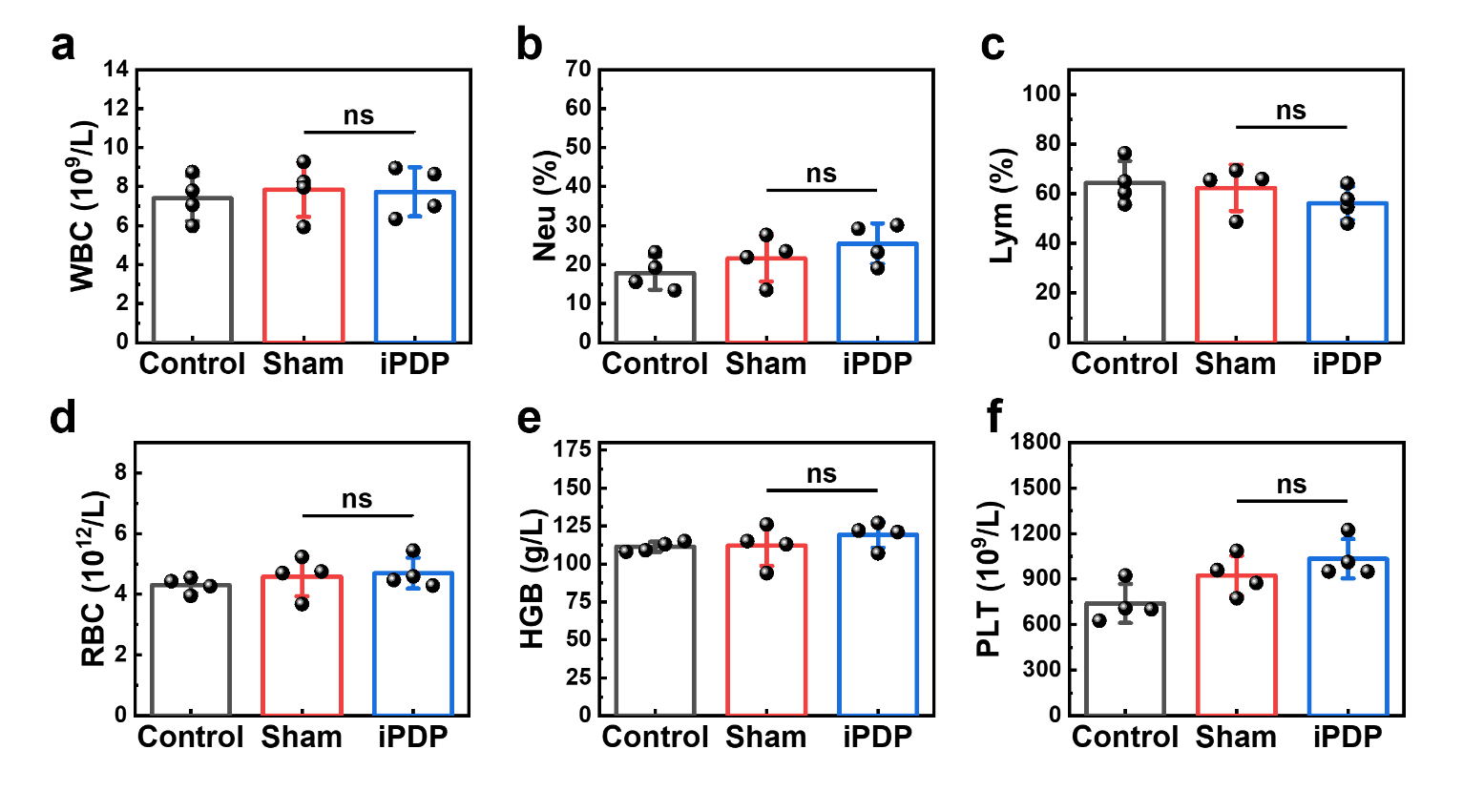


**Figure S36.** Complete blood count analysis on day 7 post-operation: (a) White blood cell (WBC) count, (b) Neutrophil (Neu) percentage, (c) Lymphocyte (Lym) percentage, (d) Red blood cell (RBC) count, (e) Hemoglobin (HGB) concentration, (f) Platelet (PLT) count. Data are presented as mean ± SD (*n* = 4), ns represents no significant difference.


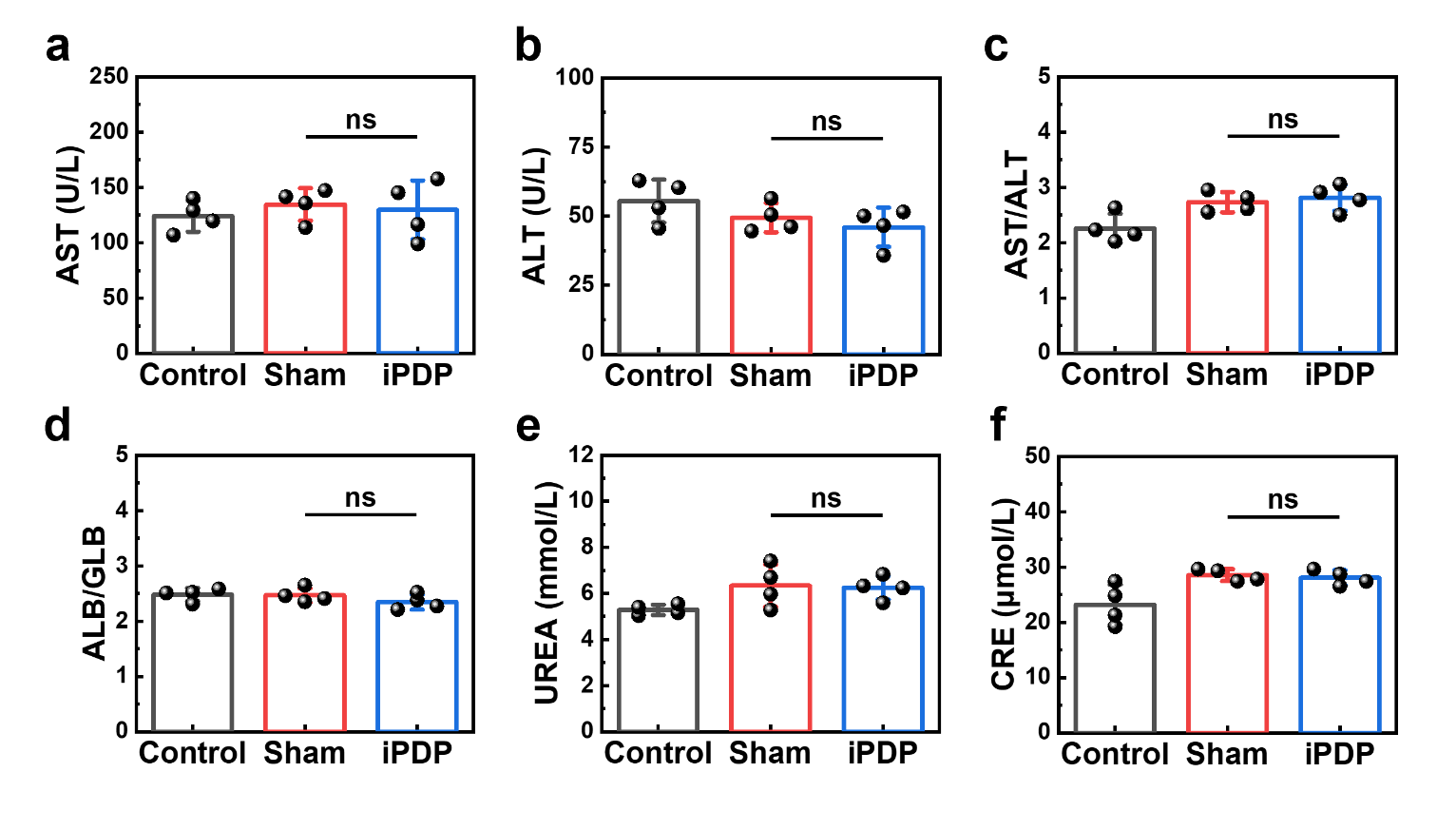


**Figure S37.** Serum biochemical analysis on day 7 post-operation: (a) AST, (b) ALT, (c) AST/ALT ratio, (d) ALB/GLB ratio, (e) UREA, (f) CRE. Data are presented as mean ± SD (*n* = 4), ns represents no significant difference.


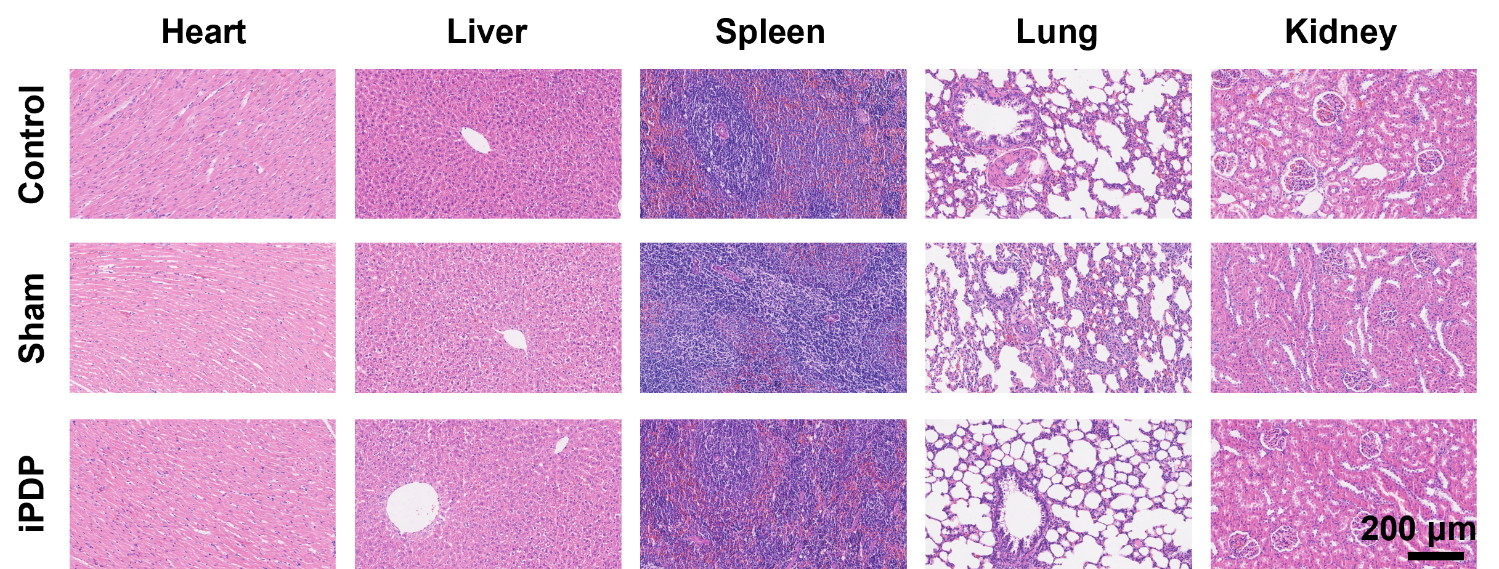


**Figure S38.** Representative H&E staining images of major organs (Heart, Liver, Spleen, Lung, Kidney) on day 7 post-operation.


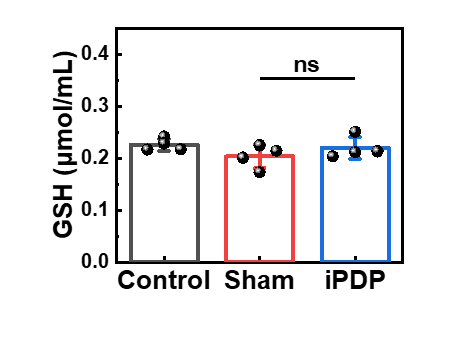


**Figure S39.** Serum glutathione (GSH) levels on day 7 post-operation. Data are presented as mean ± SD (*n* = 4), ns represents no significant difference.


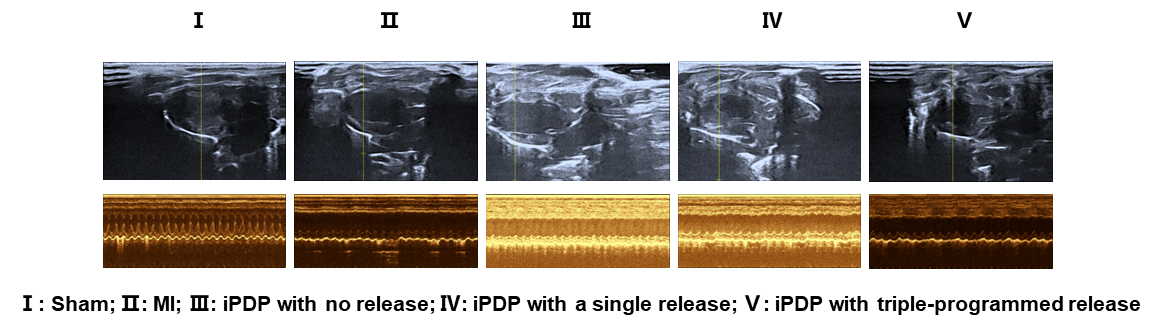


**Figure S40.** The representative echocardiographic images for various groups at 7 day.


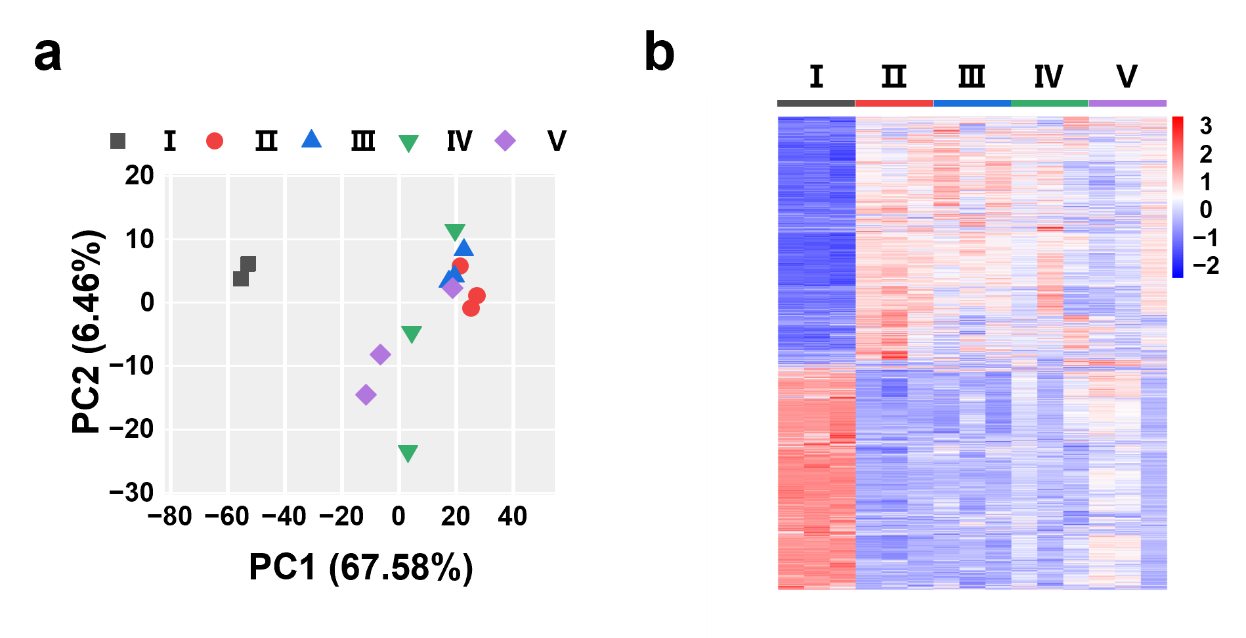


**Figure S41.** Transcriptomic profiling of infarcted heart tissues 7 days post-MI. (a) Principal component analysis of the transcriptomes across all samples. (b) Hierarchical clustering heatmap of all DEGs across the five groups. Ⅰ: Sham group; Ⅱ: MI group; Ⅲ: iPDP with no release group; Ⅳ: iPDP with a single release group; Ⅴ: iPDP with triple-programmed release group.


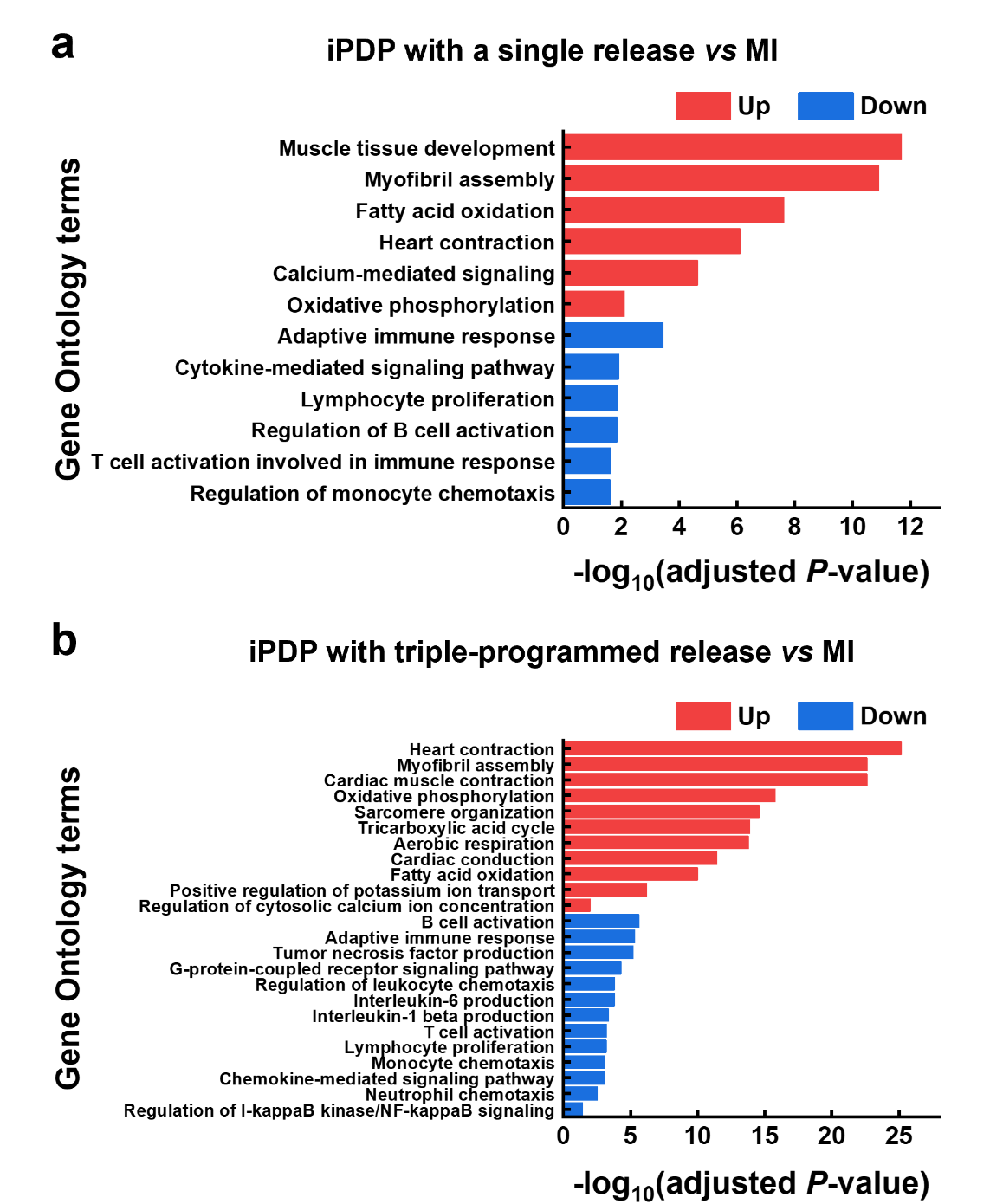


**Figure S42.** Gene Ontology (GO) enrichment analysis of heart tissues of the infarct regions from treatment groups versus the MI model. (a) Significantly enriched GO terms for genes differentially expressed between the iPDP with a single release group (Ⅳ) and the MI group (Ⅱ). (b) Significantly enriched GO terms (biological process) for genes differentially expressed between the iPDP with triple-programmed release group (Ⅴ) and the MI group (Ⅱ). Red/blue color denotes up/down regulation. Triple release (b) induced changes with greater statistical significance.


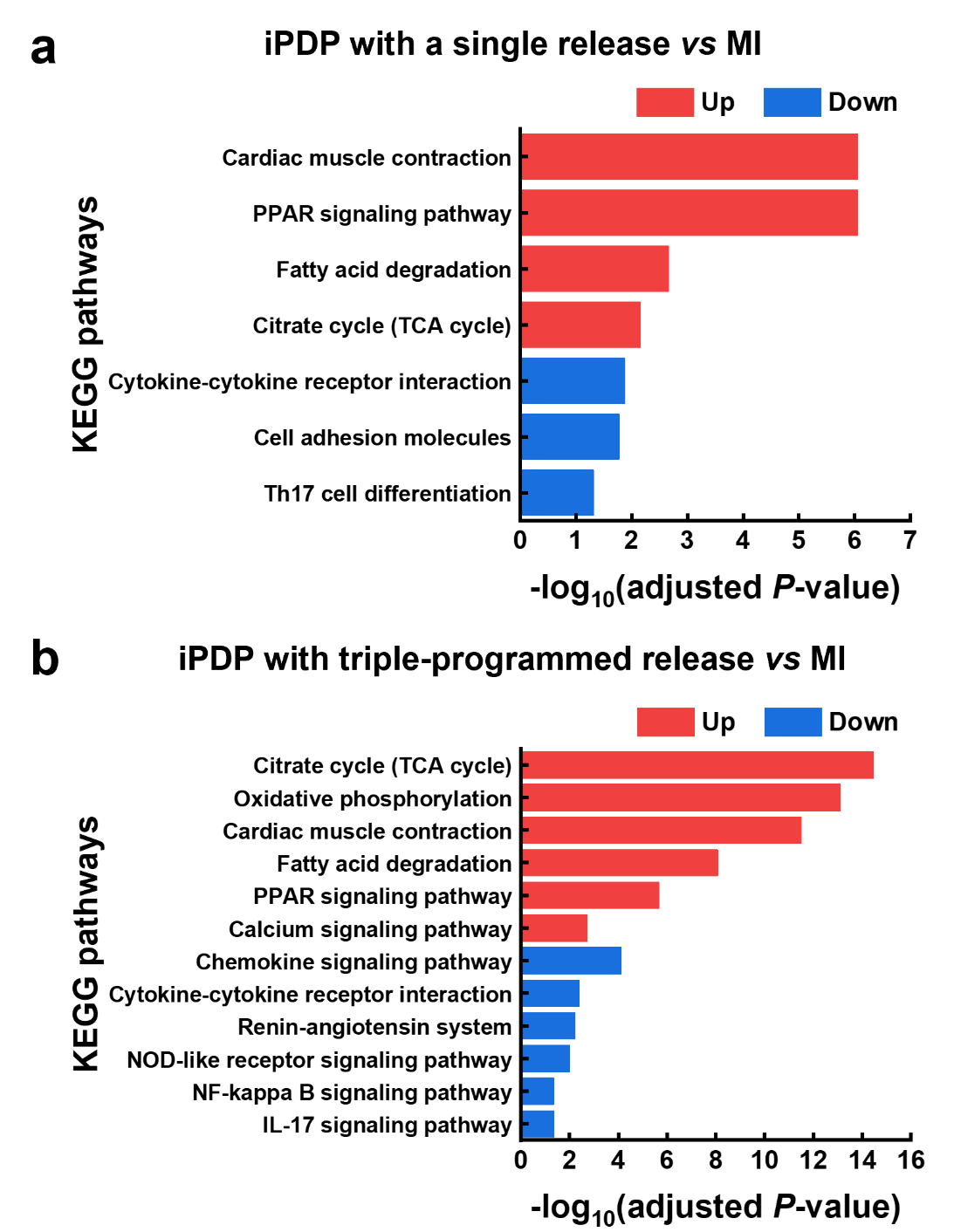


**Figure S43.** KEGG pathway enrichment analysis of heart tissues of the infarct regions from treatment groups versus the MI model. (a) Significantly enriched KEGG pathways for genes differentially expressed between the iPDP with a single release group (Ⅳ) and the MI group (Ⅱ). (b) Significantly enriched KEGG pathways for genes differentially expressed between the iPDP with triple-programmed release group (Ⅴ) and the MI group (Ⅱ). The triple-release regimen (b) activated a broader spectrum of protective metabolic pathways and more potently suppressed multiple inflammatory signaling axes compared to single release (a).


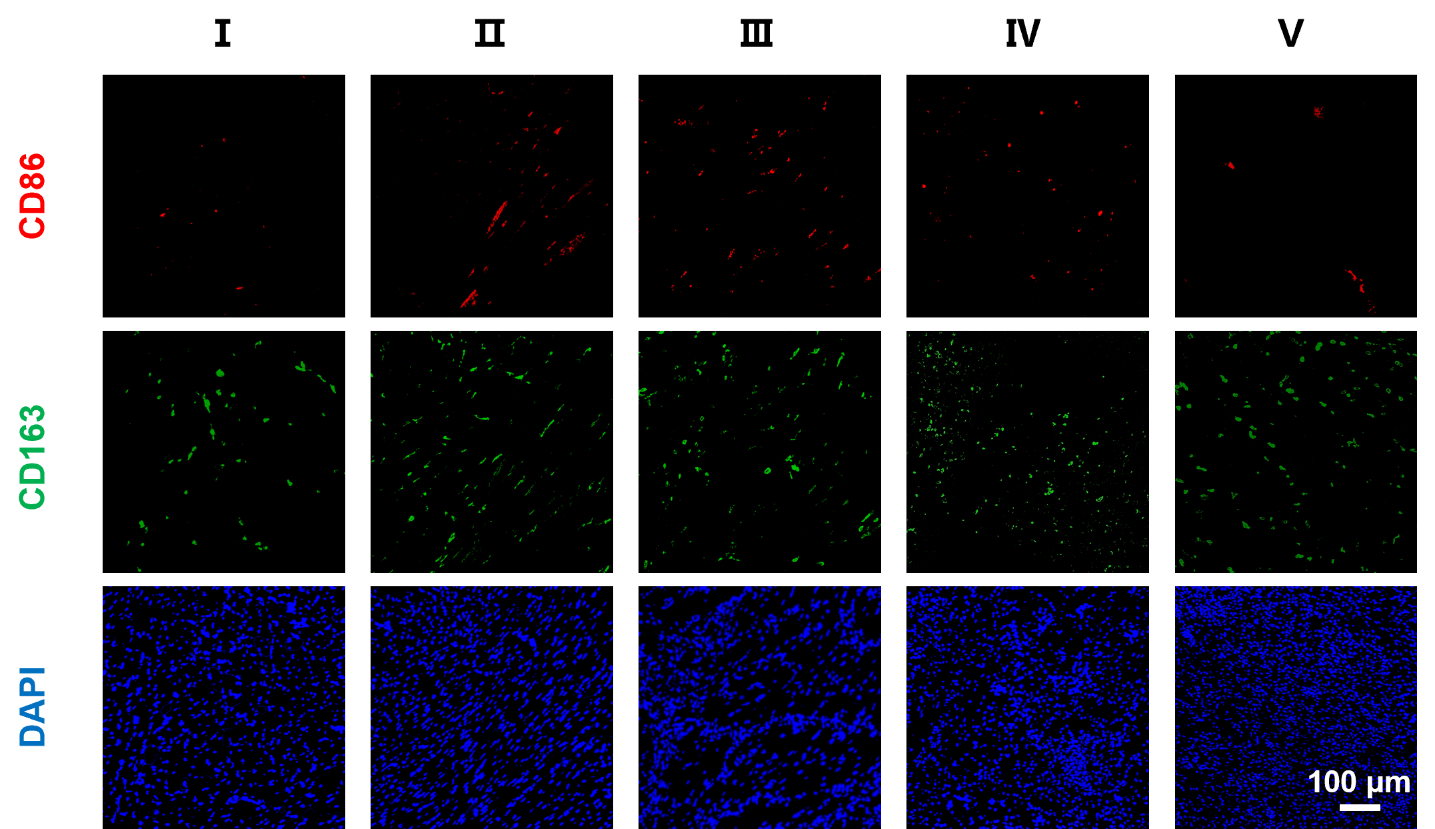


**Figure S44.** Supporting immunofluorescence images for macrophage phenotype analysis. Single-channel representative images corresponding to the merged immunofluorescence shown in **Figure 8c**. Images show CD86⁺ macrophages (M1 phenotype, red), CD163⁺ macrophages (M2 phenotype, green), and nuclei (DAPI, blue) within the infarction area. Scale bar: 100 μm. Ⅰ: Sham group; Ⅱ: MI group; Ⅲ: iPDP with no release group; Ⅳ: iPDP with a single release group; Ⅴ: iPDP with triple-programmed release group.


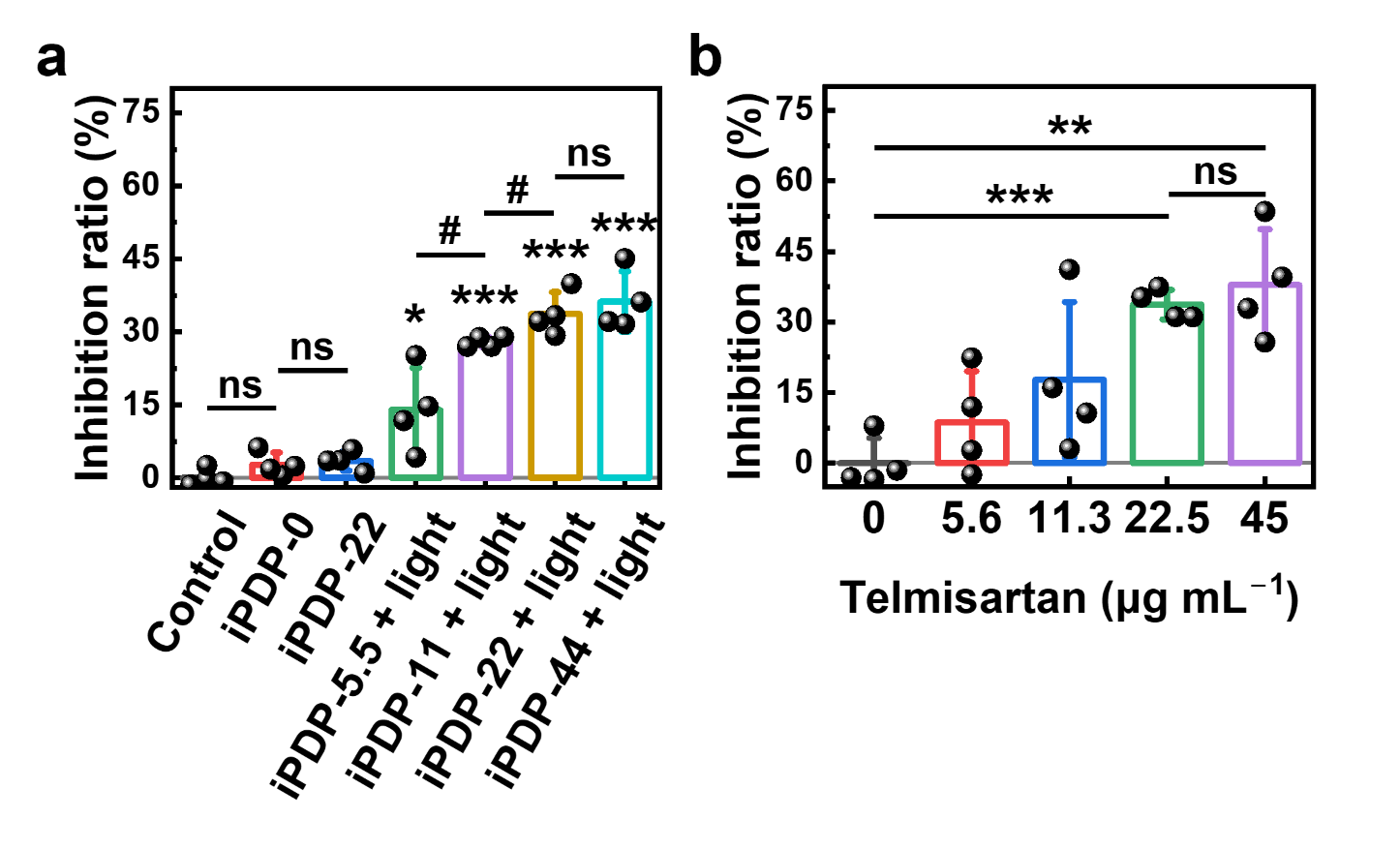


**Figure S45.** Inhibitory effect of the iPDP device on proliferation of L929 cells. (a) Proliferation inhibition rates of L929 cells after 72 h of co-culture with the iPDP device in a Transwell system. Groups are as follows: Control (cells only); iPDP-0 (device with a drug-free blank hydrogel, without irradiation); iPDP-22 (device with a prodrug hydrogel containing approximately 22 μg telmisartan, without irradiation); and devices with pre-irradiated hydrogels at graded drug loads (iPDP-5.5 + light, iPDP-11 + light, iPDP-22 + light, iPDP-44 + light, where the number indicates the approximate loaded telmisartan mass in μg). Data are presented as mean ± SD (*n* = 4). **P* < 0.05, ****P* < 0.001 versus Control group. # and ns represent *P* < 0.05 and no significant difference between the selected groups. (b) Dose-response of free telmisartan on L929 cell proliferation after 72 h of treatment. The tested concentrations (0, 5.6, 11.3, 22.5, and 45 μg mL^−1^) were set to approximate the total drug loads used in the device groups of (a). Data are presented as mean ± SD (*n* = 4). ***P* < 0.01, ****P* < 0.001, ns, not significant.


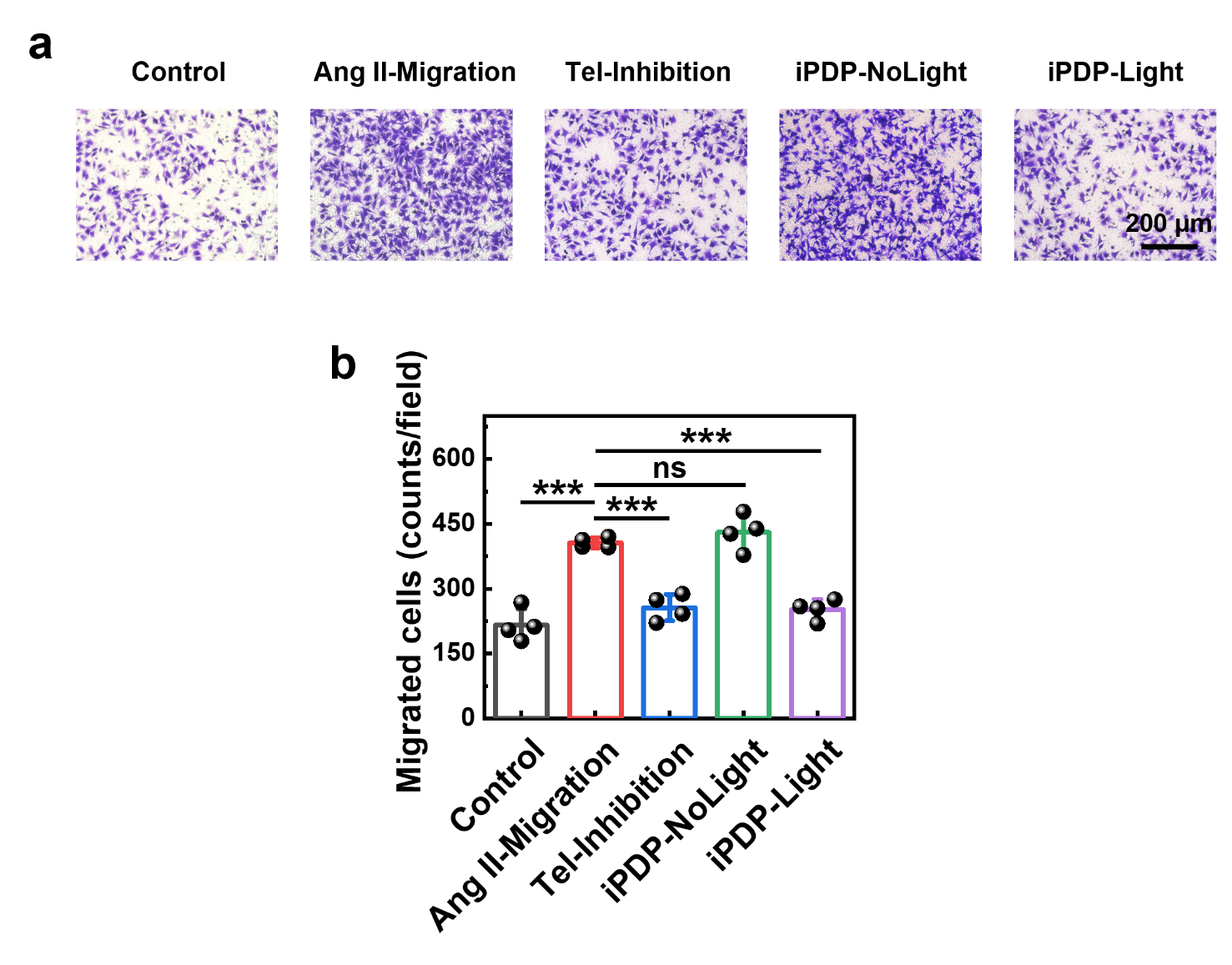


**Figure S46.** Evaluation of fibroblast migration inhibition by telmisartan released from the iPDP. (a) Representative images of transwell migration assay of L929. (b) Quantification histogram represented the number of migrated cells (*n* = 4). Scale bar: 200 μm. ****P* < 0.0001, ns, not significant.


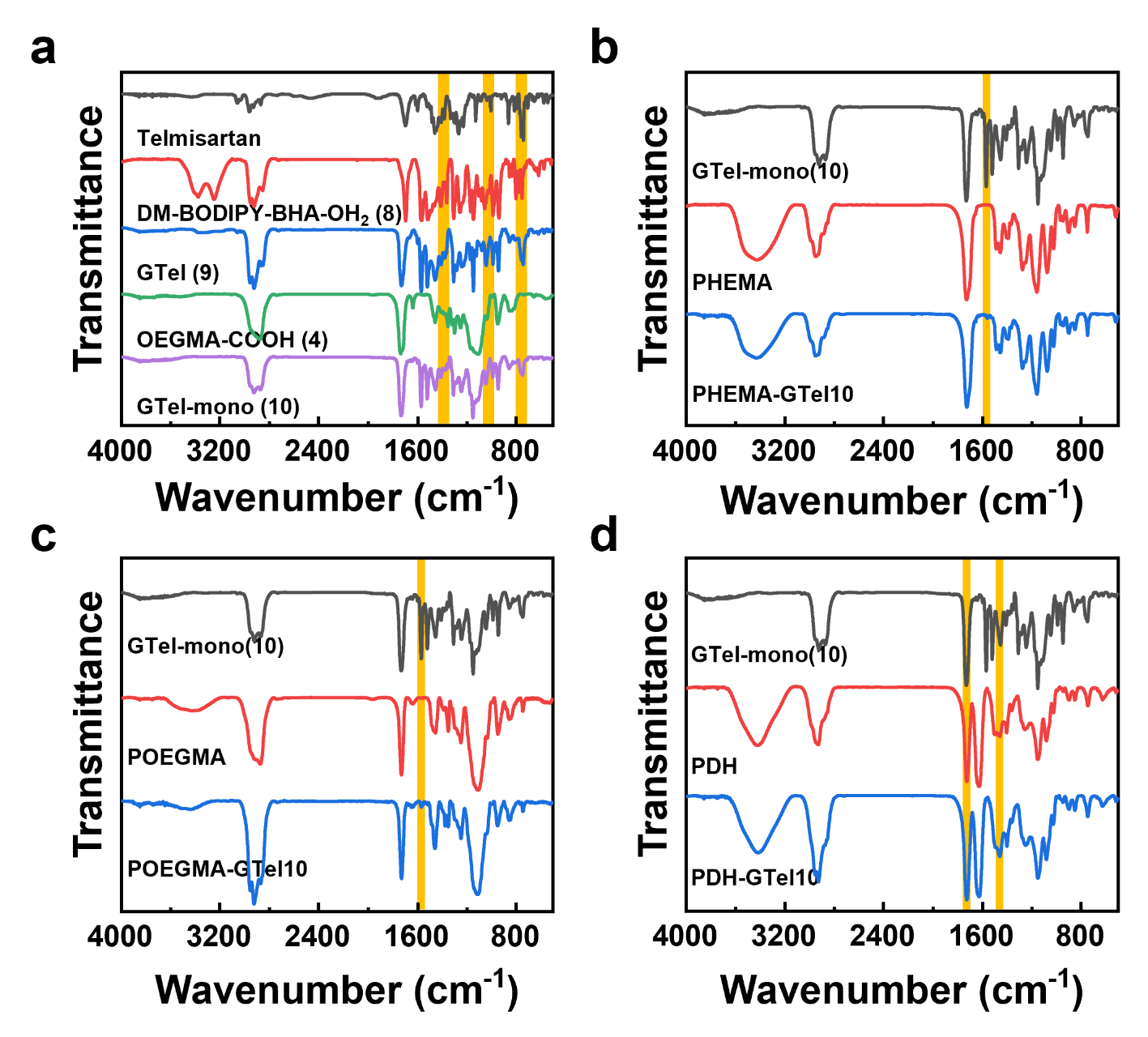


**Figure S47.** FTIR structural validation of photo-responsive prodrug monomer and polymer conjugates. (a) GTel-mono versus precursors (telmisartan/DM-BODIPY-BHA/GTel/OEGMA-COOH). (b) PHEMA-GTel10 versus PHEMA and GTel-mono. (c) POEGMA-GTel10 versus POEGMA and GTel-mono. (d) PDH-GTel10 versus PDH and GTel-mono. For PHEMA-GTel and POEGMA-GTel, a distinct new peak appears at 1568 cm^−1^ (indicated by yellow vertical lines) compared to their respective blank hydrogels, confirming the successful incorporation of GTel-mono. For PDH-GTel, enhanced absorptions are observed at 1730 cm^−1^ (ester carbonyl stretching) and 1458 cm^−1^ (yellow vertical lines) relative to the blank PDH hydrogel, providing clear evidence for the successful integration of the prodrug monomer into the copolymer network.


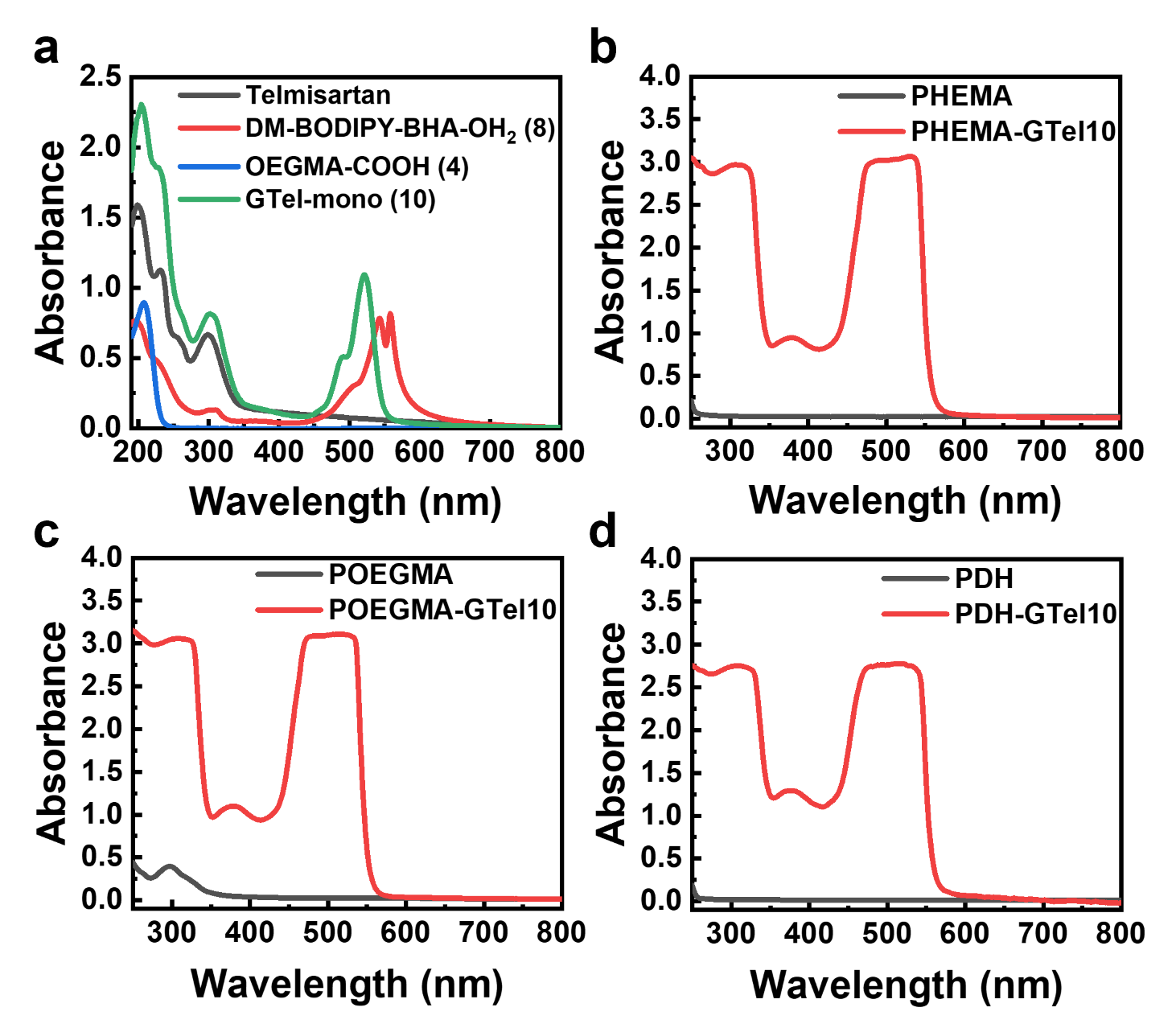


**Figure S48.** UV–vis absorption spectral analysis of photo-responsive prodrug monomer and polymer conjugates. (a) GTel-mono versus precursors (telmisartan/DM-BODIPY-BHA/OEGMA-COOH). (b) PHEMA-GTel10 versus PHEMA and GTel-mono. (c) POEGMA-GTel10 versus POEGMA and GTel-mono. (d) PDH-GTel10 versus PDH and GTel-mono. All three GTel-containing hydrogels exhibit the characteristic absorption profile of GTel-mono with distinct peaks at 299, 490, and 521 nm, corresponding to the DM-BODIPY and telmisartan chromophores. Their corresponding blank hydrogels show negligible absorption in the same region, confirming that the signals arise from the incorporated prodrug.


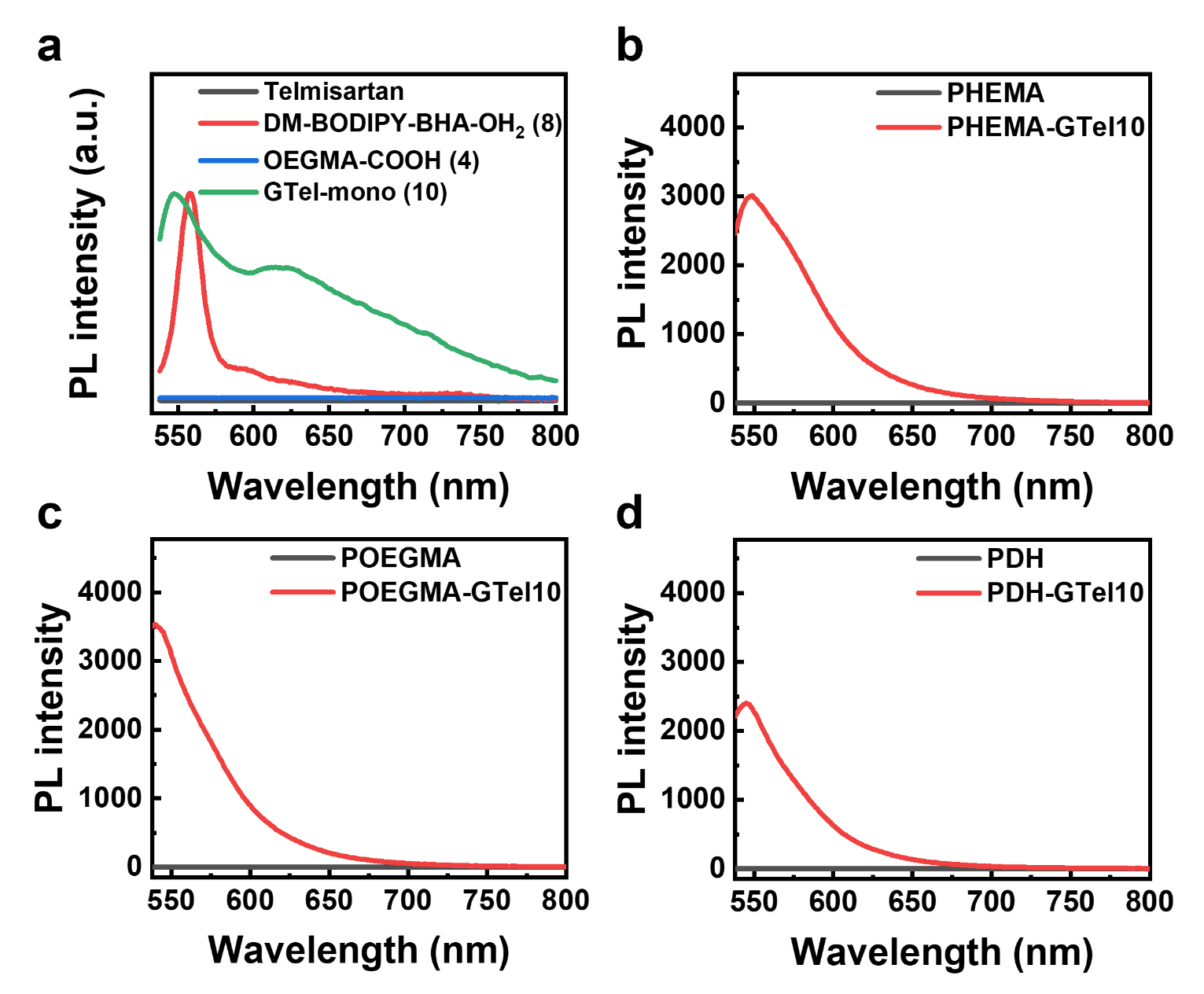


**Figure S49.** PL emission spectral analysis of photo-responsive prodrug monomer and polymer conjugates. (a) GTel-mono versus precursors (telmisartan/DM-BODIPY-BHA/OEGMA-COOH). (b) PHEMA-GTel10 versus PHEMA and GTel-mono. (c) POEGMA-GTel10 versus POEGMA and GTel-mono. (d) PDH-GTel10 versus PDH and GTel-mono. *λ*_ex_ = 520 nm. All three GTel-containing hydrogels display a broad emission band centered at ~550 nm. In contrast, free GTel-mono solution shows distinct dual peaks at 547 and 614 nm, corresponding to DM-BODIPY monomer and excimer emissions, respectively. The broadened profile in the hydrogels results from restricted molecular motion within the crosslinked network, which suppresses excimer formation, confirming that the prodrug is successfully incorporated and its photophysical behavior is consistently modulated by network confinement across all hydrogel compositions.


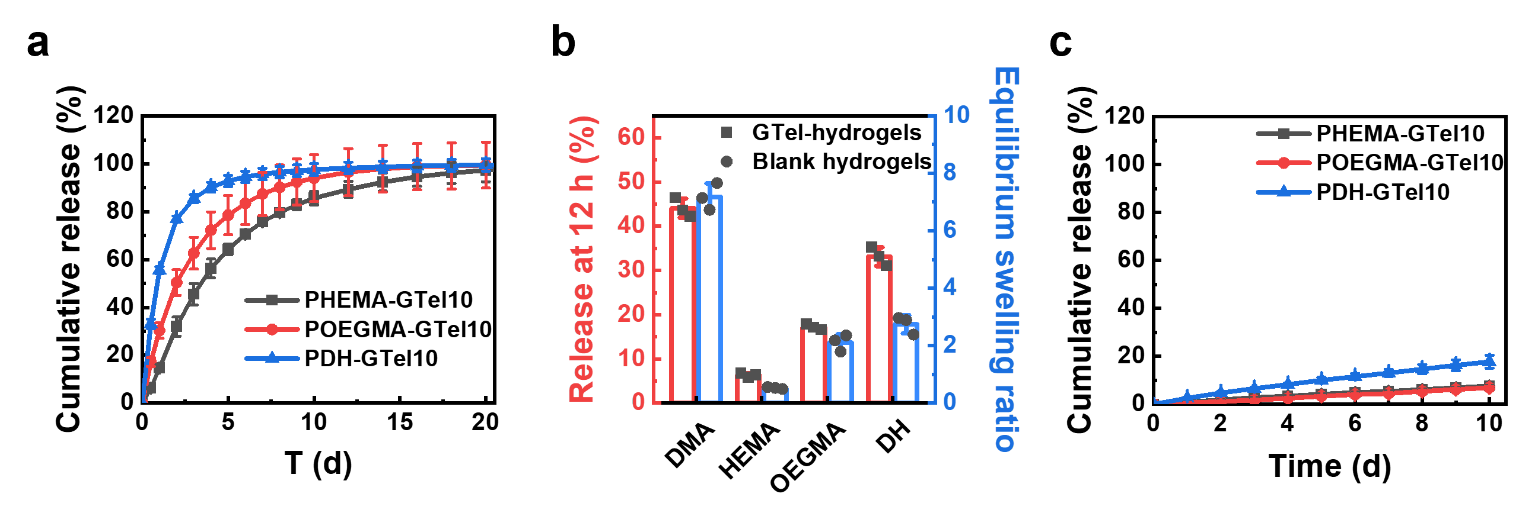


**Figure S50.** Characterization of release kinetics and stability of GTel-hydrogels with different monomer compositions. (a) Cumulative release profiles of three GTel-hydrogels (PHEMA-GTel10, POEGMA-GTel10 and PDH-GTel10) following a single illumination. (b) Correlation between the 12-hour drug release from the GTel-hydrogels (left axis) and the equilibrium swelling ratio of their corresponding blank hydrogels (right axis). (c) Drug leakage profiles of the GTel-hydrogels maintained under dark conditions without illumination. Data are presented as mean ± SD (n = 3).

The drug release rate exhibited a strong correlation with the hydrophilicity of the hydrogel network. This relationship was confirmed by the distinct release profiles of the three single‑monomer‑based hydrogels: PDMA‑GTel10 (fastest), POEGMA‑GTel10 (intermediate), and PHEMA‑GTel10 (slowest) (**Figure 2h** and Figure S50a). Quantitatively, PDMA‑GTel10 released 44.4 ± 1.2% within 12 h and 97.7 ± 2.3% over 10 days, whereas PHEMA‑GTel10 released only 6.3 ± 0.4% at 12 h and 85.6 ± 2.8% at 10 days. The hydrophilicity order of the hydrogel networks was independently confirmed by the equilibrium swelling ratios of their corresponding blank hydrogels (PDMA > POEGMA > PHEMA) (Figure S50b). To further validate this relationship, PDH‑GTel10 was prepared by copolymerizing the most hydrophilic (DMA) and the most hydrophobic (HEMA) monomers at an equal mass ratio. The hydrophilicity of the DH copolymer, as reflected by the intermediate swelling ratio of the PDH blank hydrogel (Figure S50b, was between that of the PDMA and PHEMA homopolymers. Correspondingly, the drug release rate of PDH‑GTel10 ranked between those of PDMA‑GTel10 and PHEMA‑GTel10. These findings demonstrate that the hydrophilicity of the primary monomer provides a straightforward handle for tuning the release kinetics of the prodrug‑loaded hydrogel. Minimal drug leakage was observed for all hydrogels under dark conditions, confirming their storage stability prior to photoactivation (Figure S50c).

**4. Supporting Tables**

**Table S1.** Feed composition of monomers for the GTel-hydrogels prepared in this study^a)^


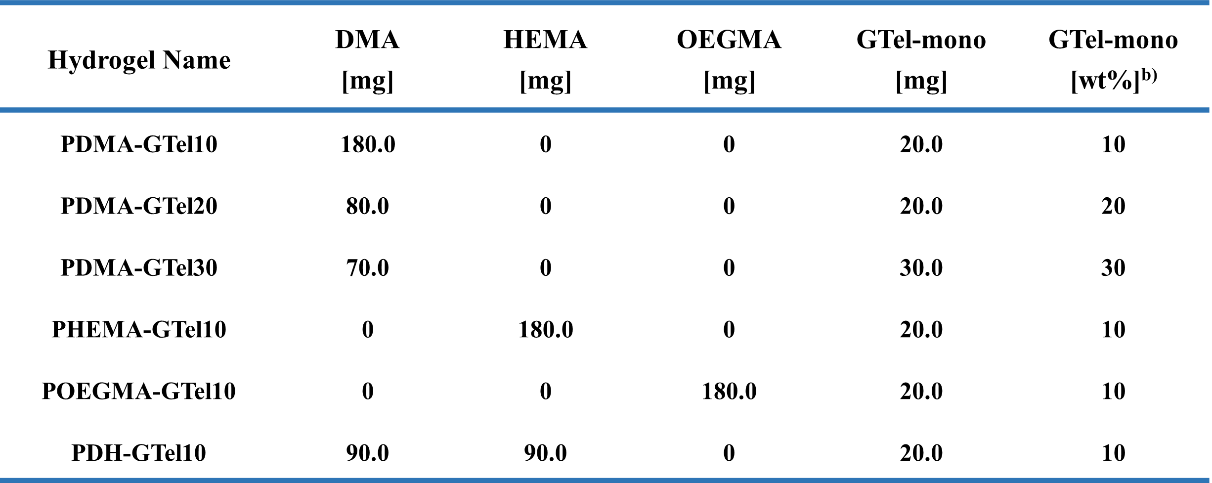


^a)^The crosslinker ethylene glycol dimethacrylate was used at a fixed ratio of 0.5 mg per 100 mg of total monomers. All polymerizations were conducted at 25 °C for 24 h using an initiation system of 13 μL of ammonium persulfate (24 mg mL^−1^ aqueous solution) and 2.5 μL of *N*,*N*,*N*′,*N*′-tetramethylethylenediamine per 100 mg of total monomers; ^b)^The wt% represents the mass percentage of GTel-mono relative to the total mass of monomers.

**Table S2.** Physical properties of the PDMA‑GTel20 hydrogel, PDMS, and the iPDP device.

^
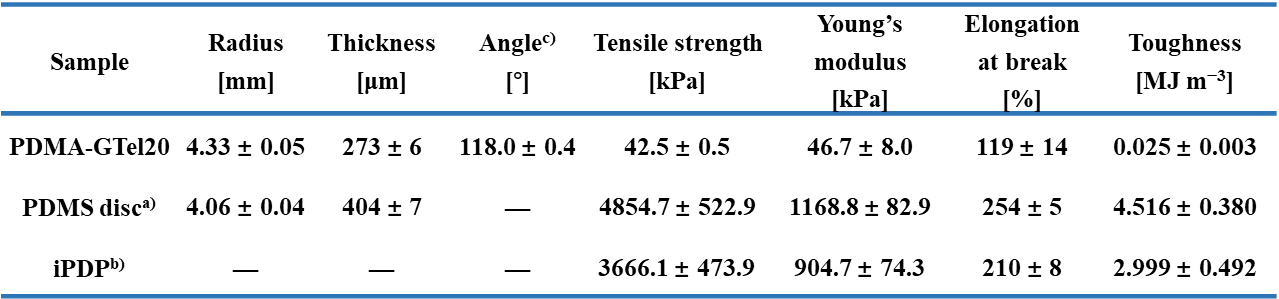
^

^a)^The optical fiber integrated within the PDMS disc has an outer diameter of 246 ± 10 µm and a length of approximately 40 cm; ^b)^ The iPDP device is a dumbbell‑shaped composite of PDMS and PDMA‑GTel20 hydrogel, with total thickness matching that of the actual implantable device (PDMS disc + swollen hydrogel layer). ^c)^The angle refers to the central angle of the hydrogel sector. Data are presented as mean ± SD (*n* = 3).
